# KRAS is vulnerable to reversible switch-II pocket engagement in cells

**DOI:** 10.1038/s41589-022-00985-w

**Published:** 2022-03-21

**Authors:** James D. Vasta, D. Matthew Peacock, Qinheng Zheng, Joel A. Walker, Ziyang Zhang, Chad A. Zimprich, Morgan R. Thomas, Michael T. Beck, Brock F. Binkowski, Cesear R. Corona, Matthew B. Robers, Kevan M. Shokat

**Affiliations:** 1 Promega Corporation, Madison, WI USA; 2 Department of Cellular and Molecular Pharmacology, Howard Hughes Medical Institute, University of California San Francisco, San Francisco, CA USA

**Keywords:** Small molecules, Chemical tools, Cancer therapy, Cell signalling, NMR spectroscopy

## Abstract

Current small-molecule inhibitors of KRAS(G12C) bind irreversibly in the switch-II pocket (SII-P), exploiting the strong nucleophilicity of the acquired cysteine as well as the preponderance of the GDP-bound form of this mutant. Nevertheless, many oncogenic KRAS mutants lack these two features, and it remains unknown whether targeting the SII-P is a practical therapeutic approach for KRAS mutants beyond G12C. Here we use NMR spectroscopy and a cellular KRAS engagement assay to address this question by examining a collection of SII-P ligands from the literature and from our own laboratory. We show that the SII-Ps of many KRAS hotspot (G12, G13, Q61) mutants are accessible using noncovalent ligands, and that this accessibility is not necessarily coupled to the GDP state of KRAS. The results we describe here emphasize the SII-P as a privileged drug-binding site on KRAS and unveil new therapeutic opportunities in RAS-driven cancer.

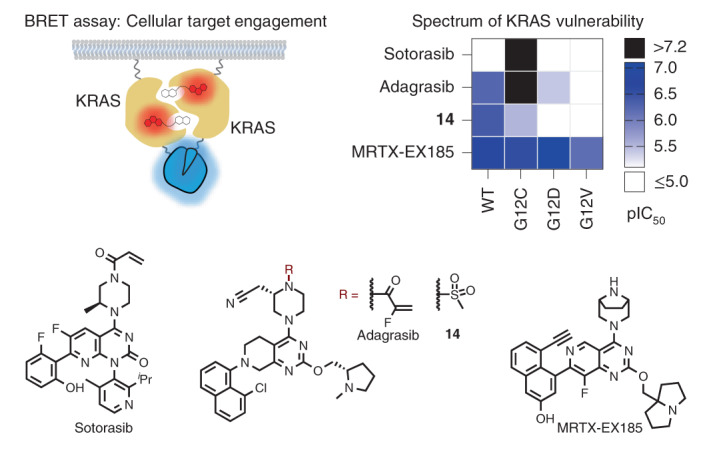

## Abstract (web summary)

The use of NMR spectroscopy and development of a cellular BRET KRAS engagement assay revealed that noncovalent ligands can access the switch-II pocket of KRAS hotspot mutants.

## Main

The *KRAS* proto-oncogene is the most frequently mutated oncogene in cancer^1^. Glycine 12 mutations are the most common, with *KRAS*(G12D) representing the most common substitution in pancreatic ductal adenocarcinoma and colorectal tumors^1^. KRAS proteins had long been considered “undruggable” until the identification of covalent drugs targeting KRAS(G12C)^2,3^. The drug sotorasib (AMG510, **1**) was recently approved for treatment of patients with the *KRAS*(G12C) mutation, and six additional drugs targeting this same mutant are currently under clinical investigation^4–6^. Several features to *KRAS*(G12C) enabled this allele to be the first *KRAS* mutant to be drugged. The somatic mutation of glycine 12 to cysteine provided the opportunity to exploit covalent drug-discovery methods that are not applicable to the other common *KRAS* alleles (for example, G12D/G12V/Q61H). Sotorasib and other known irreversible KRAS(G12C) drugs bind to the SII-P and only engage the inactive GDP state of KRAS(G12C)^2,5–10^. A rare example of a molecule reported to target the active GTP state was recently disclosed that relies on a “molecular glue” mechanism involving the recruitment of cyclophilin not widely applicable to other KRAS(G12C) inhibitors^11^. Although the KRAS(G12C) protein is insensitive to GTPase activating protien (GAP)-mediated hydrolysis, this allele is unique among the *KRAS* oncogenes in maintaining near wild-type (WT) intrinsic GTPase activity—thereby allowing for successful GDP-state targeting for this allele^12^. To effectively inhibit other oncogenic *KRAS* alleles that do not adequately sample the GDP state in cells, drugs that bind reversibly to the GTP state will probably be required.

Studies using engineered proteins and cyclic peptides to probe the SII-P of KRAS have revealed the dynamic nature of this pocket and support the possibility that KRAS−GTP may adopt conformations favorable to SII-P engagement^13–17^. However, proteins and most cyclic peptides are impermeable to cell membranes, making them difficult to use as drug leads. The recent flurry of drug discovery aimed at targeting KRAS reported in the literature and patent filings might provide suitable small-molecule leads for reversible KRAS inhibition. However, the nucleotide state requirements of these molecules are unknown. Although reporter-based methods to query downstream signaling from KRAS have been described^18^, robust methods to directly measure in-cell engagement of noncovalent ligands at KRAS are unknown.

In this study, we investigated the reversible binding of KRAS small-molecule inhibitors to determine which hotspot mutants are vulnerable to SII-P engagement in cells. We used HSQC NMR spectroscopy to directly observe reversible binding to the SII-P of KRAS in vitro and determine the nucleotide state dependency of binding. We developed a bifunctional cell-permeable fluorescent probe from the SI/II pocket inhibitor BI-2852 (**2**), and this probe was utilized in a competitive bioluminescence resonance energy transfer (BRET) format^19–21^ to quantify SII-P engagement to multimeric RAS complexes in live cells. These studies represent an observation and quantification of direct target engagement of non-G12C oncogenic KRAS mutants in cells by reversible binders. Our results expose a wide scope of vulnerability to SII-P engagement across hotspot mutants and should help guide the development of future inhibitors and therapeutics.

## Results

### Reversible KRAS SII-P binding is observed by NMR spectroscopy

**Fig. 1:**
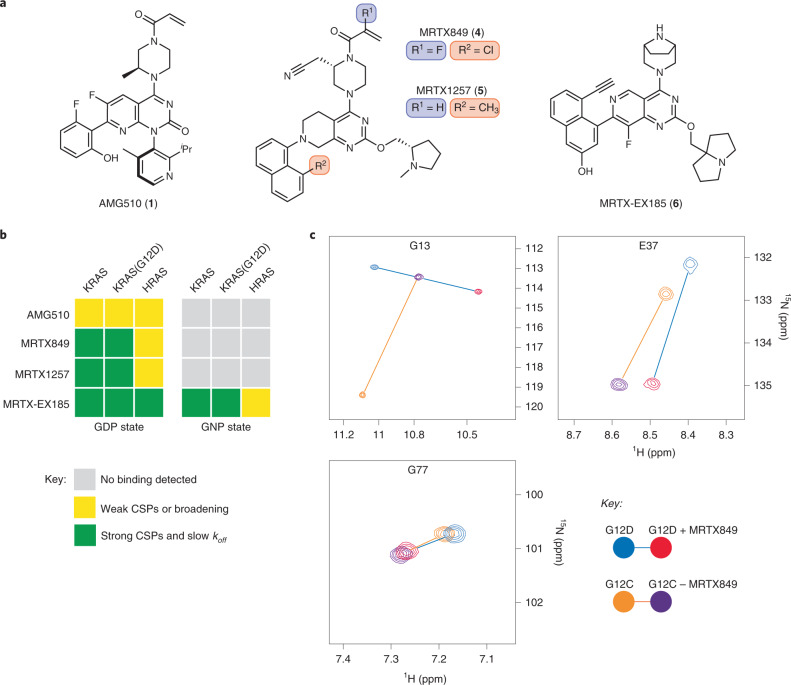
In vitro noncovalent binding to the KRAS SII-P determined by NMR spectroscopy. **a**, Chemical structures of AMG510 (**1**), MRTX849 (**4**), MRTX1257 (**5**) and MRTX-EX185 (**6**). **b**, Summary of the effects of SII-P binders on ^1^H−^15^N HSQC NMR spectra of RAS proteins. **c**, Examples of CSPs of GDP-loaded KRAS in the presence of MRTX849 and comparison of irreversible binding to KRAS(G12C) and reversible binding to KRAS(G12D). Spectra recorded at pH 7.4 and 298 K with 100 µM U-^15^N protein and 200 µM ligand.

The well-established and clinically validated inhibitors of KRAS(G12C) such as ARS-1620 (**3**), AMG510 and MRTX849 (**4**) (Fig. 1a) rely on a covalent reaction with the nucleophilic cysteine 12 in the GDP state. Although these molecules bind the same pocket and are similar chemotypes, MRTX849—and the closely related MRTX1257 (**5**)—possess unique structural elements proposed to increase the reversible component of their binding, resulting in a measurable *K*_i_ of 3.7 µM for the reaction of MRTX849 with KRAS(G12C)^7^. We sought to determine whether these molecules also bind RAS proteins lacking the G12C mutation and whether their reversible affinity is specific to the inactive GDP state.

Cell-free analysis of binding to RAS proteins was performed via protein-observed NMR spectroscopy (Fig. 1b,c). We expressed uniformly ^15^N-labeled KRAS 1–169, KRAS(G12C) 1–169, KRAS(G12D) 1–169 and HRAS 1–166 proteins and acquired a series of ^1^H–^15^N HSQC NMR spectra (Supplementary Note 1). The addition of either MRTX849 or MRTX1257 (200 µM) to the GDP-loaded state of either KRAS or KRAS(G12D) protein (100 µM) resulted in the formation of a new complex with strong chemical shift perturbations (CSPs) from the peaks of the unbound protein (Fig. 1b,c and Supplementary Note 1, spectra 2, 3, 6 and 7). The same CSPs were observed in samples containing substoichiometrically ligated proteins (100 µM protein and 30 µM ligand; Supplementary Note 1, spectrum 25), indicating that these ligands tightly bind the proteins with *k*_off_ values smaller than the frequency separation between peaks of bound and unbound proteins (<80 Hz). Although the lack of chemical exchange poses a challenge to assigning most peaks of the protein–ligand complexes to their respective residues, some key residues of the covalent KRAS(G12C)–MRTX849 and the noncovalent KRAS(G12D)–MRTX849 complexes could be reassigned from three-dimensional ^1^H−^15^N−^1^H NOESY-HSQC spectra. Similarities in perturbations of these residues (excepting glycine 13) support a similar binding conformation between the noncovalent (G12D) and covalent (G12C) complexes of MRTX849 (Fig. 1c, Supplementary Fig. 1 and Supplementary Note 1, spectra 26 and 27).

By contrast, no effects were observed on the spectra of KRAS or KRAS(G12D) proteins containing the nonhydrolyzable GTP analog GPPNHP (GNP) with 200 µM of either MRTX849 or MRTX1257 (Fig. 1b and Supplementary Note 1, spectra 14, 15, 18 and 19). Furthermore, only concentration-dependent peak broadening and weak CSPs were observed from the addition of either molecule to GDP-loaded HRAS 1–166 under the same conditions (Supplementary Note 1, spectra 10 and 11), suggesting only weak occupancy of the HRAS SII-P even at the highest concentration tested (100 µM protein and 300 µM ligand). The results of these HSQC NMR experiments show that MRTX849 and MRTX1257 bind KRAS proteins with high selectivity for the inactive GDP-loaded state and for the K-isoform over HRAS.

A series of similar ^1^H−^15^N HSQC NMR experiments provided some evidence for weak binding of AMG510 (Fig. 1a) to the SII-P of GDP-loaded KRAS and HRAS proteins (Fig. 1b and Supplementary Note 1, spectra 1, 5 and 9). Peaks corresponding to residues in the SII-P broadened and exhibited weak (generally less than line-widths) CSPs in the presence of 200 µM of AMG510. These experiments suggest that the reversible affinity of AMG510 to RAS proteins is probably too weak to be relevant to in-cell experiments conducted at lower concentrations, and that AMG510 must rely on the irreversible reaction at the mutant cysteine 12 for its inhibitory activity, which is consistent with previously published data^5^.

Recently, compounds reported to target KRAS(G12D) were disclosed in patent applications by multiple groups^22–25^. We selected and synthesized an example from Mirati Therapeutics patent filings^25^ (MRTX-EX185, **6**) with structural features similar to MRTX849/1257 (Fig. 1a). We found that MRTX-EX185 bound GDP-loaded KRAS and KRAS(G12D) by HSQC NMR spectroscopy (Fig. 1b and Supplementary Note 1, spectra 4, 8 and 28), but in stark contrast to MRTX849/1257, MRTX-EX185 also bound the active GNP state of these proteins (Supplementary Note 1, spectra 16 and 20). Furthermore, MRTX-EX185 also bound GDP-loaded HRAS (Supplementary Note 1, spectrum 12). In each of these five cases, identical CSPs were observed in samples containing either 100 µM protein and 200 µM ligand or containing 100 µM protein and 30 µM ligand (substoichiometric), indicating that the *k*_off_ values for these complexes are small (Supplementary Note 1, spectrum 25). When a substoichiometric amount of ligand (50 µM) was added to a sample containing both GDP- and GNP-loaded KRAS proteins (100 µM each), the GDP–KRAS–MRTX-EX185 complex formed exclusively, and the same experiment with KRAS(G12D) yielded the same result (Supplementary Fig. 2 and Supplementary Note 1, spectra 29 and 30). These results suggest that the relative affinity of MRTX-EX185 to the GDP state over the GNP state of KRAS proteins is greater than the noise limit of the spectra (more than ten for most peaks).

These cell-free NMR experiments show that MRTX849 and MRTX1257 engage KRAS proteins even in the absence of a nucleophilic mutant cysteine 12. However, this engagement is selective for the inactive GDP-loaded state of the protein. The more recently disclosed MRTX-EX185, by contrast, engages both nucleotide states—albeit with preference for the inactive GDP-loaded protein—and might present an opportunity to inhibit even constitutively active (GTP-loaded) KRAS hotspot mutants. However, these NMR experiments require high concentrations of proteins and do not quantify the potency of these tightly binding compounds. Furthermore, in vitro binding assays may not be representative of the in-cell vulnerability of a regulated, effector-bound and membrane-localized protein such as KRAS.

### Observing reversible KRAS SII-P occupancy in cells with BRET

With our NMR results supporting the potential of KRAS and its hotspot mutants to be vulnerable to noncovalent SII-P occupancy, we asked whether these SII-P ligands engage KRAS in cells. We first assessed the antiproliferative effects of MRTX849 in a number of G12C and non-G12C *KRAS* mutant and *KRAS* WT cell lines (Supplementary Fig. 3a). Although MRTX849 inhibited the growth of SW-1990 (*KRAS*(G12D)) and HCT-116 (*KRAS*(G13D)) at micromolar concentrations, it also had the same effect on HEK293 (*KRAS* WT, *RAS*-independent)^26^ and A375 (*BRAF* V600E, *RAS*-independent), suggesting the antiproliferative effects may originate from *RAS*-independent toxicity (Supplementary Fig. 3a). We also measured the ability of MRTX849 to inhibit extracellular signal-related kinase (ERK) phosphorylation in a similar panel of cell lines (Supplementary Fig. 3b). We corroborated the strong potency of MRTX849 in *KRAS*(G12C)-driven cell lineages. However, in non-G12C driven cell lineages, the nonspecific cytotoxic effects were observed over the same concentration range as the inhibitory effects on ERK phosphorylation (Supplementary Fig. 3b), thus preventing a clear confirmation of cellular target engagement.

**Fig. 2:**
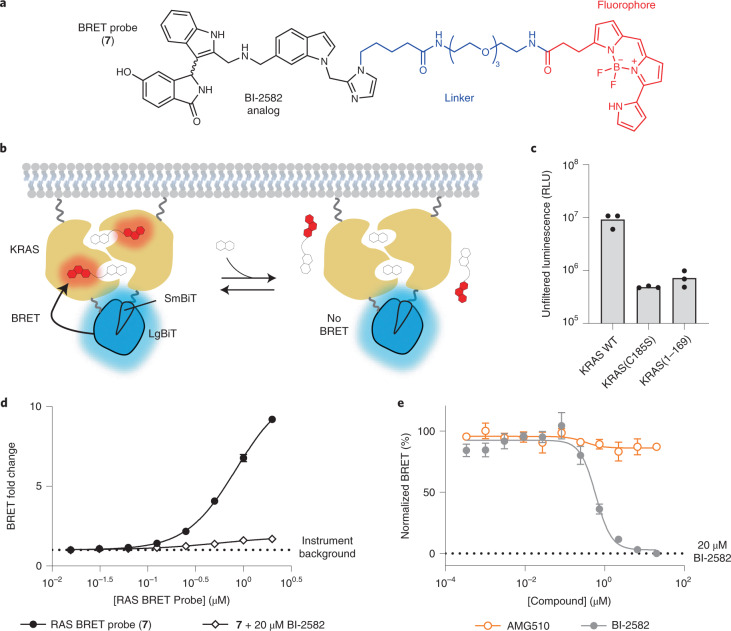
Target engagement assay for RAS. **a**, Chemical structure of SI/II-P RAS BRET probe (**7**). **b**, Illustration of the RAS cellular target engagement assay. A luminescent complex is formed between RAS multimers in live cells; competitive displacement of the BRET probe reduces BRET signal while leaving the NanoBiT complex intact. Target engagement leads to competitive displacement of the BRET probe at RAS dimers. **c**, Removal of the hyper variable region or mutation at C185S results in a loss of luminescence in HEK293 cells compared with full-length KRAS WT, supporting RAS lipidation signals as critical to assay signal (left). Results are the mean ± s.e.m. of three independent experiments (*n* = 3). **d**, RAS BRET probe dose response for KRAS WT. BRET fold change is calculated by normalizing the BRET ratio of each point by the BRET ratio in the absence of BRET probe (instrument background). The BRET signal was competed by 20 µM BI-2852 (**2**), demonstrating specificity. Data are the mean ± s.e.m. of three independent experiments (*n* = 3). **e**, Live cell target engagement is observed at WT KRAS with BI-2852, but not with AMG510 (**1**). BRET is normalized relative to a saturating (20 µM) dose of BI-2852, as marked. RLU stands for relative luminescence units. Source data

The interference from off-target toxic effects in these assays precluded the analysis of target engagement and prompted us to develop new approaches to determine ligand–RAS interaction in cells. To more directly query biophysical engagement of KRAS and HRAS with small-molecule target engagement in cells, a BRET reporter system was developed. We synthesized a pan-RAS BRET probe (**7**) by conjugating a fluorophore to a derivative of the reversible SI/II-P inhibitor BI-2852 (ref. ^27^) (Fig. 2a). Recognizing the multimeric and membrane-localized nature of RAS^28–31^, we sought to generate a BRET signal conditionally within membrane-associated RAS complexes^32^. We configured a luminescent complementation-based system (NanoBiT)^21^ that was dependent upon RAS lipidation as the BRET donor (Fig. 2b,c and Supplementary Figs. 4 and 5). Luminescent imaging confirmed the membrane localization of RAS dimers in live cells (Supplementary Fig. 4). Furthermore, homodimeric NanoBiT-RAS was functionally validated using an intracellular CRAF-Ras binding domain (RBD)-HaloTag interaction assay (Supplementary Fig. 5a−d) and was competent to activate phospho-ERK (p-ERK) in cells (Supplementary Fig. 5e). Removal of critical lipidation residues (C185S or removal of the hyper variable region) resulted in a dramatic decrease in luminescence, supporting the need for membrane anchoring for the RAS multimerization (Fig. 2c and Supplementary Fig. 5f). Titration of BI-2852 did not impact the RAS dimerization NanoBiT signal in live cells, supporting that RAS is constitutively oligomerized in this assay system (Supplementary Fig. 5g). When cells expressing the BRET donor complexes were treated with the SI/II-P RAS BRET probe **7**, we observed a strong BRET signal that was readily competed to instrument background by unmodified BI-2852 in cells (Fig. 2d,e and Supplementary Fig. 6). Moreover, BI-2852 had no effect on the BRET or luminescence in an irrelevant target engagement assay (Supplementary Fig. 6l), confirming specificity in the competitive effects observed in the RAS assays. Hill coefficients for BI-2852 across all RAS variants studied ranged from 1.3 to 3.3 with a mean of 1.9 ± 0.7, consistent with cooperative binding of two BI-2852 molecules to a dimeric RAS, as proposed previously^33^. Competitive engagement results with a *tert*-butyloxycarbonyl-protected precursor to the RAS BRET probe (**8**) indicated that the linker functionalization of the BI-2852 derivative resulted in a decrease in affinity to RAS (Supplementary Fig. 7), but that affinity was still sufficient to yield a strong BRET signal between dimeric RAS species and the RAS BRET probe in live cells.

To determine whether the RAS BRET probe binds with a mechanism similar to that of BI-2852, we introduced a mutation (D54R) that is expected to abolish probe engagement on the basis of structural analysis (Supplementary Fig. 8a). As expected, mutation of D54 to R in KRAS resulted in complete loss of BRET with the RAS BRET probe (Supplementary Fig. 8C) without effecting dimerization (Supplementary Fig. 8b). KRAS(D54R) dimers were still competent to engage CRAF-RBD effectors in cells, as evidenced by its interaction with CRAF-RBD-HaloTag (Supplementary Fig. 8d). Although CRAF-RBD was able to interact with KRAS(D54R) dimers, BI-2852 was unable to inhibit the interaction, further validating this mutation as a negative control (Supplementary Fig. 8d). Overexpression of full-length CRAF also attenuated the BRET observed between KRAS and the RAS BRET probe (Supplementary Fig. 8e), further supporting that the probe binds with a mechanism similar to BI-2852 and is competitive with effector interactions.

**Fig. 3:**
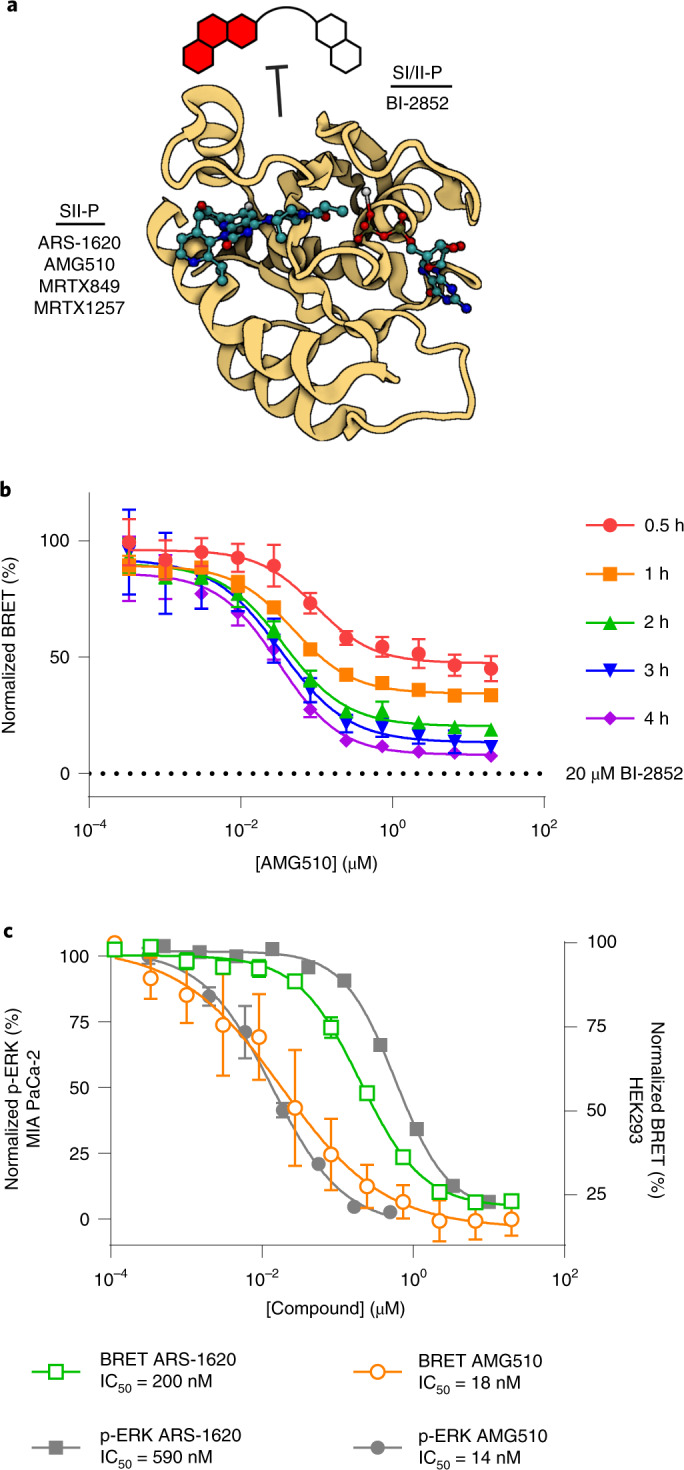
Measuring SII-P engagement of KRAS(G12C). **a**, The SI/II BRET probe is used to quantify binding of unmodified SII-P ligands to RAS in live cells. Image created with BioRender.com from PDB 6OIM (AMG510-KRAS(G12C)). **b**, Protracted KRAS(G12C) engagement is observed for AMG510 (**1**). Data are representative of two independent experiments (*n* = 2), and individual data points are the mean ± s.d. of four technical replicates. **c**, Comparison of BRET target engagement data after 2 h at KRAS(G12C) versus p-ERK from MIA PaCa-2 cells as reported^5^. Data are means of four independent experiments ± s.e.m. (*n* = 4). Source data

To evaluate the sensitivity of the SI/II-P BRET probe to allosteric target engagement within the SII-P, live HEK293 cells expressing NanoBiT–KRAS(G12C) were challenged with SII-P ligands AMG510 or ARS-1620 in the presence of the RAS BRET probe (Fig. 3a). Time- and dose-dependent competition was observed between AMG510 or ARS-1620 and the RAS BRET probe (Fig. 3b and Supplementary Fig. 9a,b). Unlike for BI-2852, AMG510 and ARS-1620 produced Hill coefficients closer to unity (0.8 and 1.1, respectively), consistent with a lack in cooperativity in the binding to the SII-P of KRAS(G12C). At a 2 h timepoint, BRET results with both AMG510 and ARS-1620 closely matched the potency of endogenous target engagement and p-ERK inhibition at identical timepoints in a number of G12C driven lineages (MIA PaCa-2, NCI-H358), corroborating the accuracy of the BRET method as a proxy for engagement in an endogenous cellular setting (Fig. 3C and Supplementary Fig. 9b). AMG510 demonstrated exquisite engagement selectivity for KRAS(G12C) compared with KRAS WT, other KRAS hotspot mutants, and HRAS WT (Supplementary Fig. 9c), consistent with previous reports for functional selectivity between KRAS(G12C) and non-G12C driven cancer cell lines. Like BI-2852, AMG510 did not impact the luminescence produced by NanoBiT–KRAS(G12C) (Supplementary Fig. 9d). Additional SII-P inhibitors were evaluated at KRAS(G12C) complexes, including MRTX849 and MRTX1257 (Supplementary Fig. 9e). Each produced BRET target engagement results that agreed closely with published cellular potency at KRAS(G12C) lineages^7^. MRTX849/1257 were the most potent KRAS(G12C) inhibitors in the analysis, in close agreement with previous studies^7^. Together the results for engagement of KRAS(G12C) with SII-P ligands support the potential of the BRET target engagement system to report on KRAS in its endogenous cellular setting, and that this system can be used to accurately query engagement across oncogenic KRAS mutants in live cells.

**Fig. 4:**
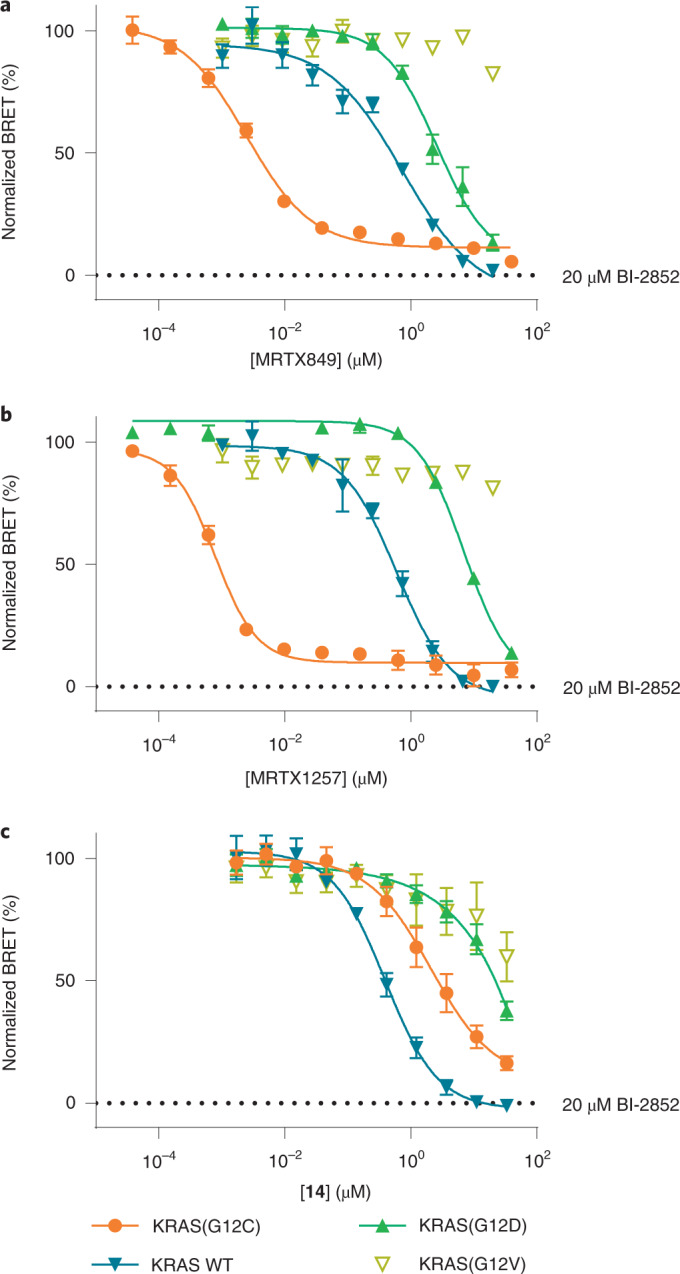
SII-P binders engage KRAS WT and G12 hotspot mutants. **a**−**c**, BRET target engagement profiles of MRTX849 (**4**) (**a**), MRTX1257 (**b**) and noncovalent methylsulfonamide derivative **14** (**c**). BRET is normalized relative to a saturating (20 µM) dose of BI-2852, as marked. **a**,**b**, Data are representative of three independent experiments, and each data point is the mean of three or four technical replicates ± s.d. (*n* = 3). **c**, Each data point is the mean of three independent experiments ± s.e.m. (*n* = 3). Source data

**Fig. 5:**
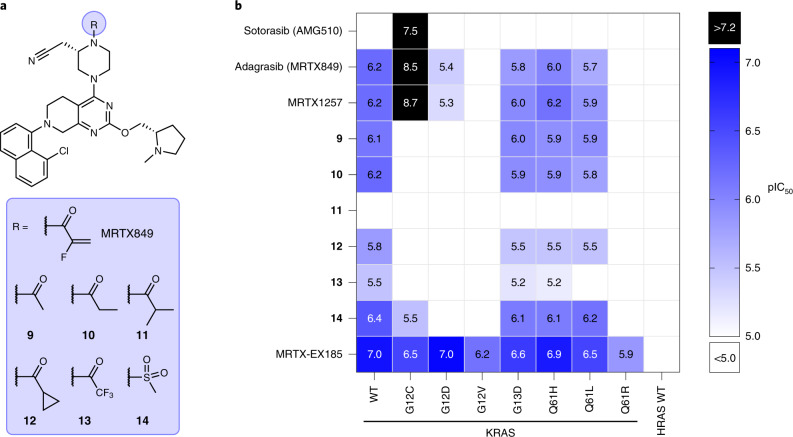
Profiling in-cell target engagement of SII-P binding molecules. **a**, Chemical structures of MRTX849 (**4**) and noncovalent derivatives **9**–**14**. **b**, Summary of BRET target engagement across KRAS hotspot mutants and HRAS. pIC_50_ values were calculated as –log_10_(IC_50_ [M]). Combinations that exhibited incomplete engagement at the highest concentration tested (10^−4^ M) were grouped as pIC_50_ < 5.0 (white cells). Source data

This BRET target engagement system enabled us to directly assess the engagement of WT KRAS and numerous critical hotspot mutants by SII-P ligands. For example, following 2 h incubation, engagement of both MRTX849 and MRTX1257 was observed for WT KRAS complexes in the submicromolar range (half-maximum inhibitory concentration (IC_50_) ~ 600 nM) (Fig. 4a,d, Supplementary Table 1 and Supplementary Fig. 10a). When KRAS hotspot mutants were evaluated, a wide spectrum of engagement was observed (Figs. 4a,b and 5b, Supplementary Table 1 and Supplementary Fig. 10b−g). Although no engagement of MRTX849/1257 was observed for KRAS G12V and Q61R, modest engagement was observed for the remaining KRAS hotspot mutants in the single-digit micromolar range (IC_50_ ranging from 1 to 5 µM). Among the KRAS hotspot mutants excluding KRAS(G12C), the most potent engagement was observed for G13D, Q61H and Q61L (Supplementary Fig. 10d−f).

WT HRAS as well as two oncogenic HRAS mutants (G12C and G12V) were also evaluated for SII-P vulnerability using the BRET assay. No engagement was observed for WT HRAS with AMG510, MRTX849 or MRTX1257 (Supplementary Figs. 9c and 10h). Although HRAS(G12V) was also not vulnerable to SII-P engagement (Supplementary Fig. 10i), HRAS(G12C) showed vulnerability to both AMG510 and MRTX849 (Supplementary Fig. 10j). AMG510 demonstrated similar intracellular affinity towards HRAS(G12C) compared with KRAS(G12C), but MRTX849 demonstrated affinity for HRAS(G12C) that was three orders of magnitude weaker than that observed for KRAS(G12C), suggesting that the MRTX849 scaffold preferentially engages the K-isoform of RAS, which is consistent with our NMR spectroscopy results.

We next sought to accurately assess the contribution to SII-P engagement from noncovalent ligand–protein interactions. Because of the potential differences in the steric and electrostatic environments for the SII-P among the RAS variants, we synthesized derivatives of MRTX849 lacking the covalent acrylamide warhead and positioning groups with varied steric and electronic properties proximal to residue 12 (**9**–**14**, Fig. 5a), and we evaluated these compounds with the BRET target engagement assay (Fig. 5b, Supplementary Table 1 and Supplementary Fig. 11a). For most RAS variants, the saturated amide and sulfonamide derivatives (**9**−**14**) demonstrated comparable rank order vulnerability with those of MRTX849 and MRTX1257. Among non-G12C variants, WT KRAS remained the most vulnerable of all RAS isoforms to reversible engagement, followed closely by hotspot KRAS mutants G13D, Q61H and Q61L. KRAS(G12V), KRAS(Q61R) and WT HRAS showed weak to no engagement across all saturated amides, similar to the results observed with MRTX849/1257. Engagement of KRAS(G12C) by most of the saturated amide derivatives was impaired in the absence of the covalent mechanism, with the exception of the sulfonamide (**14**), which demonstrated modest affinity (3.0 µM, Fig. 4c).

Within the saturated amide series, **9** and **10** containing acetamide and propionamide moieties, respectively, were generally well tolerated. The methylsulfonamide derivative **14** was also well tolerated, in most cases demonstrating comparable engagement potency with the **9** and **10**, except in the case of KRAS(G12C) where it was found to be moderately selective compared with other derivatives. The electron-deficient trifluoroacetamide **13** demonstrated right-shifted moderate to weak potency in all cases compared with **9** and **10**, suggesting the importance of polar interactions with the amide carbonyl^34^. The bulky *iso*-butyramide **11** demonstrated the weakest engagement potency among all of the amides across all RAS isoforms. Compound **11** also caused an increase in the BRET signal for some RAS variants (Supplementary Fig. 11a), which was probably related to cytotoxicity (Supplementary Fig. 11b,c). Posing a ring constraint to the branched isopropyl group (that is the cyclopropyl carboxamide presented in **12**) improved the potency compared with **11**, but still demonstrated only moderate to weak potency in most cases. These saturated amides elicited cytotoxic effects in a RAS-independent cell line at similar concentrations (Supplementary Fig. 11b,c); however, our BRET system still permitted the direct measurement of SII-P engagement without prohibitive interference from off-target toxicity.

### MRTX-EX185 engages KRAS mutants and drives antiproliferation

**Fig. 6:**
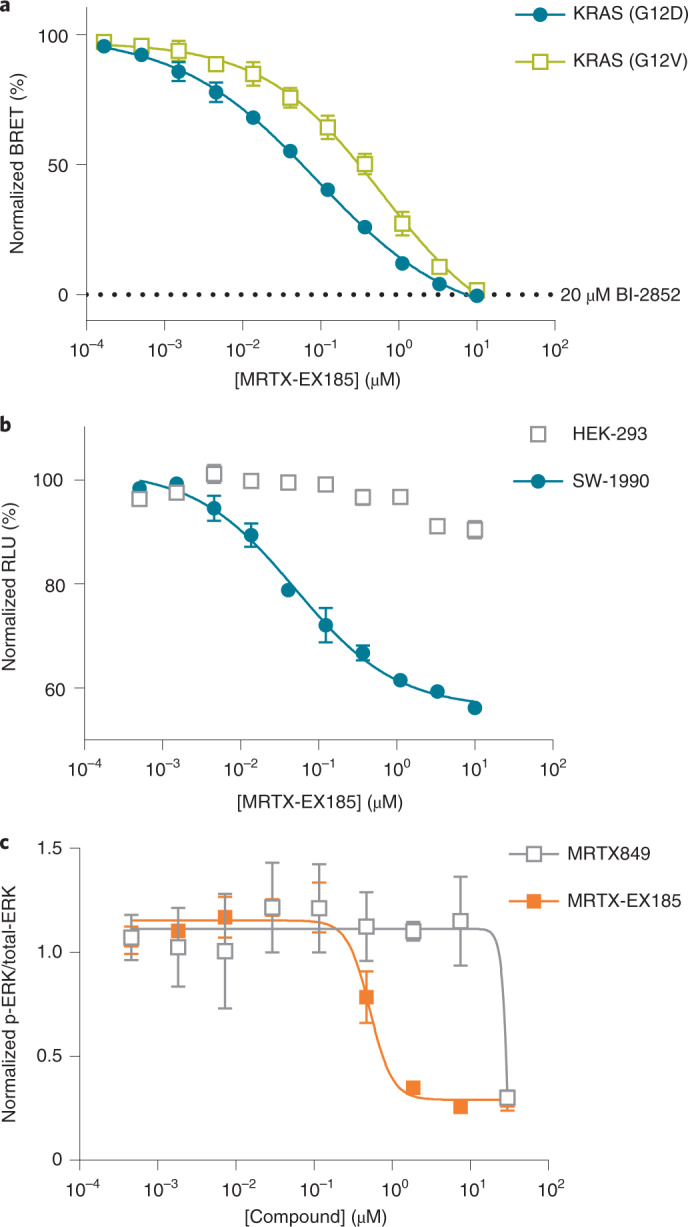
Characterization of engagement of KRAS with MRTX-EX185. **a**, BRET target engagement profiles for MRTX-EX185 (**6**) at KRAS(G12D) and KRAS(G12V). BRET is normalized relative to a saturating (20 µM) dose of BI-2852 (**2**), as marked. Data are means of three independent experiments ± s.e.m. (*n* = 3). **b**, EX185-driven antiproliferation (CellTiter-Glo) is observed in SW-1990 (*KRAS*(G12D)) but not in HEK293 (*KRAS*-independent) cells. Data are the means ± s.e.m. of three independent experiments, each performed with at least three technical replicates (*n* = 3). **c**, EX185 inhibits p-ERK. Data are means ± s.e.m. of three independent experiments (*n* = 3). Source data

Because our NMR results demonstrated the unique capability of MRTX-EX185 to bind to both the GDP state and the GTP state of KRAS(G12D) in a cell-free system, we next evaluated this compound in a cellular setting using our BRET assay. Potent target engagement (Figs. 5b and 6a and Supplementary Table 1; IC_50_ value of 90 nM) was observed for MRTX-EX185 with KRAS(G12D), greatly surpassing the engagement potency of the GDP-state-specific MRTX849 derivatives. Although MRTX-EX185 and MRTX849 have some similar structural features, it is unknown whether both molecules engage KRAS in a similar pose within the SII-P. To provide support for engagement of MRTX-EX185 within the KRAS SII-P, we also assayed engagement of KRAS(Y96D), which contains a previously reported mutation conferring resistance to described SII-P inhibitors including MRTX849 (ref. ^11^) (Supplementary Fig. 12a). KRAS(Y96D) engagement was not observed with MRTX849, **10** or MRTX-EX185 by the BRET-based assay. This finding, in conjunction with the finding that all of the MRTX chemotypes in this study show weak to no binding to HRAS variants (in which residue 95 is a glutamine), is consistent with binding to the SII-P in a similar pose.

MRTX-EX185 was also evaluated for inhibition of KRAS(G12D):effector interactions in cells using a NanoBiT protein–protein interaction assay. MRTX-EX185 demonstrated time- and dose-dependent inhibition of the KRAS(G12D):CRAF(RBD) interaction (Supplementary Fig. 12b), providing support for functional disruption of MAPK signaling. The protracted inhibition of CRAF-RBD interactions may be due to the need for overexpression of the RBD, which would be expected to stabilize KRAS–GTP. Thus, evaluation of KRAS(G12D):CRAF interactions with endogenous proteins may be warranted to more accurately query the kinetics of pathway inhibition. p-ERK and cell viability analysis in SW-1990 cells confirmed that engagement with MRTX-EX185 translated into inhibition of mitogenic signaling and an antiproliferative effect in a G12D-driven lineage (Fig. 6b,c), with antiproliferative potency (70 nM) in close agreement with the BRET readout. Unlike the MRTX849 derivatives, nonspecific cytotoxicity did not confound the antiproliferative results, because MRTX-EX185 did not inhibit proliferation in a panel of control cell lines (Supplementary Fig. 12c). Taken together, these results along with the NMR findings indicate that GTP-state compatibility may support the superior SII-P engagement for KRAS(G12D) in cells. We therefore attempted to extend the utility of MRTX-EX185 to additional KRAS hotspot mutants. MRTX-EX185 engaged numerous KRAS Q61, G12 and G13 mutant proteins (Figs. 5b and 6a, Supplementary Table 1 and Supplementary Fig. 12d). Notably, MRTX-EX185 engaged KRAS(G12V) in cells, which is the most GTP-biased G12 allele described. Although the potency against individual mutants may require tailored chemical optimization, and engagement of WT KRAS may constrain the therapeutic window, our observation that a SII-P ligand can engage several GTP hydrolysis-deficient KRAS mutants signifies exciting opportunities to drug these KRAS mutants through this pocket.

## Discussion

Here we report subfamily-wide engagement of KRAS hotspot mutants with the preclinical inhibitor MRTX849 and structurally related molecules. This presents evidence of intracellular SII-P vulnerability across the prevalent oncogenic KRAS mutants including KRAS(G12D). To characterize target engagement across RAS species, we combined in vitro and intracellular biophysical approaches. NMR spectroscopy provided a defined system to observe reversible, noncovalent binding and to determine the impact of nucleotide status on KRAS vulnerability. However, cell-free methods are incapable of simulating the intracellular architecture where target engagement would naturally occur. To query engagement in cells, we developed a SI/II-P BRET probe that was competent to detect a variety of intracellular engagement mechanisms including ligands selective for either SI/II-P or SII-P. Mutually exclusive binding between the BRET probe and the SII-P ligands enabled a systematic evaluation of SII-P engagement across KRAS hotspot mutants. This mutual exclusivity is consistent with the dynamic nature of RAS effector occupancy in cells. It is probable that RAS occupancy in cells is conformationally selective, and on the basis of its small size, RAS may disfavor co-occupancy of multiple ligands or effector proteins. The BRET method reported here conditionally measures engagement at membrane-localized RAS complexes in cells. Target engagement results with known SII-P covalent inhibitors matched both engagement and MAPK inhibition within endogenous G12C driven lineages, supporting the accuracy of the engineered BRET method.

Expanding beyond inhibition of KRAS(G12C), the BRET system enabled us to observe engagement of WT KRAS and of the majority of KRAS hotspot mutants including G12D. As measured in the BRET system, the rank order vulnerability of KRAS hotspot mutants to SII-P engagement with MRTX849/1257 and related noncovalent inhibitors (**9**−**14**) did not fully correlate with reported rates of intrinsic hydrolysis using purified RAS proteins^12^. Specifically, the G13D, Q61H and Q61L mutants reportedly have among the lowest intrinsic hydrolysis rates of the hotspot mutants evaluated here, as determined in cell-free systems. Accordingly, these alleles should be among the least vulnerable to SII-P target engagement by GDP-state-specific inhibitors, even when considering the potential for steric and conformational effects to confer differential affinity. However, MRTX849/1257 and the noncovalent inhibitors **9**, **10** and **14** engaged these mutants nearly as potently as they did WT protein, and more potently than they engaged the G12D and G12V mutants. In the case of G13D, this result may be explained by the high nucleotide exchange rate measured for this mutant^12,35,36^. In the cases of the Q61 mutants, earlier reports have noted similar discrepancies; a GDP-state-specific degrader was able to target KRAS(Q61H) in cells^26^, and KRAS(Q61L) was observed to possess a higher hydrolysis rate in a cellular context than in cell-free systems^36^. These earlier reports and our in-cell BRET data suggest that the nucleotide states of RAS proteins in a cellular setting may deviate from those quantified in a biochemically defined system, emphasizing the need for direct measurements of target engagement in cells when evaluating RAS-targeted inhibitors. Another factor that we cannot rule out is the potential contribution of RAS proteins in extracellular fractions to the behavior of the cellular BRET assay. RAS proteins in extracellular fractions could potentially contribute to this cellular BRET assay, which may have different properties compared with intracellular RAS proteins.

Because MRTX849/1257 demonstrate SII-P engagement across KRAS hotspot mutants, this chemotype may serve as the basis for development of allele-specific KRAS inhibitors beyond G12C. However, the GDP-state bias might limit the efficacy of this chemotype against a wider array of KRAS hotspot mutants that may predominantly reside in a GTP state in vivo. For example, KRAS(G12D) was less vulnerable than WT, and KRAS(G12V) and Q61R were largely inaccessible to MRTX849/1257. Thus, less subtle chemical modifications will probably be necessary to target these oncogenes, and engaging both nucleotide states of KRAS may be required. At the time of preparing this manuscript, structures of new KRAS(G12D) inhibitors were disclosed that were structurally similar the MRTX849 chemotype^22–25^. We found that one such example, MRTX-EX185 (ref. ^25^), can bind GPPNHP-loaded KRAS and KRAS(G12D) 1−169 by NMR and engage KRAS(G12D) in cells by our BRET-based assay with <100 nM affinity. The increased affinity to KRAS(G12D) translated into potent inhibition of RAF effector interactions as well as potent antiproliferative effect. Although detailed analyses of this new chemotype’s binding mode have not yet been published, its ability to also access the active nucleotide state of KRAS SII-P is probably a key contributor to its increased engagement potency against KRAS(G12D) in cells. An expanded evaluation of MRTX-EX185 is warranted to determine the kinetics of inhibition and pathway inactivation in a KRAS G12D setting. Among the KRAS hotspot mutants, KRAS(G12V) is expected to be even more heavily biased towards the GTP state compared with G12D^12^. Consistent with GTP-state accessibility, MRTX-EX185 engaged KRAS(G12V) in cells with submicromolar affinity. Together our target engagement and NMR spectroscopy results support a broad opportunity to target KRAS SII-P in a manner decoupled from nucleotide status.

We have shown that KRAS hotspot mutants offer wider opportunities for SII-P engagement than previously understood; in particular, some proteins bearing activating mutations may be more accessible to GDP-state inhibition in some cellular contexts than predicted solely on the basis of biochemical GTP hydrolysis rates. Furthermore, recently disclosed chemotypes capable of directly binding the active GTP-loaded state present even wider opportunities for SII-P engagement across KRAS hotspot mutants. Thus, our work highlights the importance of methods to directly assay target engagement in cells to compliment phenotypic assays and in vitro biochemical assays. Methods to query target occupancy independent of target function may serve to characterize the broadest variety of engagement mechanisms and inhibitor chemotypes. Accordingly, the BRET target engagement method should serve as a complement to other functional assays that detect effector interactions in cells^18^. The BRET-based and NMR assays reported in this work provide a reliable workflow to rapidly profile direct target engagement across a variety of RAS hotspot mutants, which should be broadly enabling for SII-P inhibitor discovery. Similarly, these assays may also become important tools to assess KRAS secondary mutations that are already emerging in clinical settings^11,37,38^. These capabilities should aid in the evaluation and optimization of new and improved medicines for RAS-driven cancers and prevalent RASopathies.

## Methods

### Cell transfections and BRET measurements

HEK293 cells (ATCC), HeLa Cells (ATCC), A375 cells (ATCC), HCT-116 cells (ATCC), NCI-H358 cells (ATCC), NCI-H647 cells (ATCC), MIA PaCa-2 Cells (ATCC) and SW-1990 cells (ATCC) were cultured in DMEM (Gibco) + 10% FBS (Sradigm), with incubation in a humidified, 37 °C/5% CO_2_ incubator. H1975 cells (ATCC) were cultured in RPMI 1640 (Gibco) + 10% FBS, with incubation in a humidified, 37 °C/5% CO_2_ incubator.

For RAS cellular BRET measurements, a luciferase donor signal was produced at multimeric RAS using the NanoBiT approach. Amino-terminal (N-terminal) large BiT (LgBiT) or small BiT (SmBiT) RAS fusions were encoded in pNB3K and pNB4K (respectively) expression vectors (Promega), including flexible 15-residue linkers (GSSGGGGSGGGGSSG) between the tag and each RAS isoform. All *KRAS* open reading frames (ORFs) were based upon KRAS4B (UniProt P01116-2) and all *HRAS* ORFs were based upon UniProt isoform 1 (P01112-1). All RAS ORFs were full-length unless otherwise noted. HEK293 cells were transfected with SmBiT–RAS and LgBiT–RAS fusion constructs using FuGENE HD (Promega) according to the manufacturer’s protocol. Briefly, SmBiT–RAS and LgBiT–RAS constructs were diluted together into Transfection Carrier DNA (Promega) at a mass ratio of 1:1:8 in Opti-MEM (Gibco), after which FuGENE HD was added at a ratio of 1:3 (µg of DNA: µl of FuGENE HD). For example, for a 1-ml size transfection complex, 1 µg each of the SmBiT–RAS and LgBiT–RAS DNAs were combined with 8 µg of Transfection Carrier DNA in 1 ml of Opti-MEM. One part (vol) of FuGENE HD complexes thus formed were combined with 20 parts (vol) of HEK293 cells suspended at a density of 2 ×10^5^ per ml in Opti-MEM containing 1% (v/v) FBS, followed by incubation in a humidified, 37 °C/5% CO_2_ incubator for 18−24 h. The total concentrations for each plasmid were 5 ng per well for each RAS plasmid, and the total concentration of DNA was 50 ng per well. Following transfection, cells were washed with PBS solution, harvested by trypsinization and resuspended in Opti-MEM containing 1% (v/v) FBS. BRET assays were performed in white, 96-well non-binding surface plates (Corning) at a density of 2 × 10^4^ cells per well. All chemical inhibitors were prepared as concentrated stock solutions in DMSO (Sigma-Aldrich) and diluted in Opti-MEM (unless otherwise noted) to prepare working stocks. Cells were equilibrated with the RAS BRET probe and test compound before BRET measurements, with an equilibration time of 2 h unless otherwise noted. RAS BRET probe was prepared first at a stock concentration of 100× in DMSO, after which the 100× stock was diluted to a working concentration of 20× in BRET probe dilution buffer (12.5 mM HEPES, 31.25% PEG-400, pH 7.5). For RAS BRET probe dose response measurements, the RAS BRET probe was added to the cells in an eight-point, two-fold dilution series starting at a final concentration of 2 µM. For target engagement analysis, the RAS BRET probe was added to the cells at a final concentration of 1 µM. To measure BRET with the RAS BRET probe, NanoBRET Target Engagement substrate (Promega) was added according to the manufacturer’s recommended protocol, and filtered luminescence was measured on a GloMax Discover luminometer equipped with 450 nm BP filter (donor) and 600 nm LP filter (acceptor), using 0.5 s integration time. Unlike the full-length NanoLuc protein, the signal of which can be quenched in extracellular environments using an impermeable inhibitor of NanoLuc, the NanoBiT luciferase in its current form is not amenable to extracellular quenching using the same approach. Raw BRET ratios were calculated by dividing the acceptor counts by the donor counts. Milli-BRET units (mBU) were calculated by multiplying the raw BRET values by 1000. When normalized BRET was used, mBRET values were normalized using equation (1);

1 $${\rm{Normalized}}\;{\rm{BRET}}\;\left( \% \right) = \left[ {\left( {A - C} \right)/\left( {B - C} \right)} \right] \times 100$$

Where *A* = mBRET in the presence of test compound and BRET probe, *B* = mBRET in the presence of vehicle and BRET probe and *C* = mBRET in the presence of a saturating 20-µM dose of BI-2852. Apparent BRET probe affinity values (half-maximum effective concentration (EC_50_)) were determined using the sigmoidal dose response (variable slope) equation available in GraphPad Prism (equation 2);

2 $$Y\,=\,{\rm{Bottom}} + ({\rm{Top}} - {\rm{Bottom}})/\left( {1 + 10^{\left( {\left( {{\rm{logEC}_{50}} - X} \right) \times \rm{HillSlope}} \right)}} \right).$$

In some cases, the RAS BRET probe was not saturable up to the solubility limit of the BRET probe, so the EC_50_ value of the BRET probe is reported as >1 µM. For determination of test compound potency, competitive displacement data were plotted with GraphPad Prism software and data were fit to equation (2) to determine the IC_50_ value.

To measure the impact of full-length CRAF coexpression of target engagement assay behavior, experiments were performed as described above, except that DNA encoding untagged full-length CRAF (UniProt P04049-1) was substituted for the Transfection Carrier DNA.

To measure the interaction of KRAS NanoBiT dimers with CRAF-CRD-RBD-HaloTag, cells were transfected as described above, except that Transfection Carrier DNA was replaced with CRAF-CRD-RBD (residues 51−133, UniProt P04049-1)-HaloTag plasmid and cells were treated with 4% FBS overnight. During incubation with test compound (2 h for BI-2852 or 6 h for MRTX849), cells were treated with 1× NanoBRET-618 ligand and BRET was detected per the manufacturer’s instructions.

To measure target engagement and luminescence for ABL1 kinase, the NanoBRET Target Engagement Intracellular Kinase Assay, K-4 (Promega) was used according to the manufacturer’s instructions. Briefly, experiments were conducted as described above for the RAS target engagement assays, except that NanoLuc-ABL1 fusion DNA (Promega) was transfected with Transfection Carrier DNA (Promega) at a mass ratio of 1:9, tracer K-4 was used at a final concentration of 0.33 µM, and the extracellular NanoLuc inhibitor was included in the BRET detection step at a final concentration of 20 µM.

### Measurements of total LgBiT–RAS levels

Total LgBiT expression level was determined in the presence of a saturating concentration (100 nM) of high-affinity HiBiT peptide (Peptide 2.0), 1× NanoBRET Target Engagement substrate and 50 mg ml^−1^ digitonin (as a permeabilization agent). Unfiltered luminescence was measured using the Nano-Glo protocol on a GloMax Discover luminometer with a 0.3 s integration time.

### Bioluminescent Imaging of NanoBiT–KRAS fusions in live cells

All imaging experiments were performed using the LV200 bioluminescence imaging system (Olympus) equipped with an ImagEM ×2 EM-CCD camera (Hamamatsu) and a ×40 oil, 1.4 NA objective. HEK293 cells and HeLa cells were transiently transfected as described above for the RAS cellular target engagement assays. Control plasmids encoding MAPK14 and HDAC–NanoLuc fusions were from Promega. Cells were plated onto Nunc Lab-Tek II eight-well chambered coverslips (ThermoFisher Scientific) coated with 0.1% gelatin in 200 µl of growth medium (DMEM + 10% FBS) at a density of 4 × 10^5^ cells per well. After 24 h of incubation at 37 °C, 100 µl of Nano-Glo Live Cell Reagent (Promega) was added. All images were acquired with cellSens software (Olympus) using an electron multiplying gain of 600 and an exposure time of 5 s. Each image was generated using an average projection of ten images. Generation of average projections and linear adjustments of dynamic range were performed using Image J image-processing software (Fiji package).

### Measurements of disruption for KRAS(G12D):CRAF(RBD) interactions in cells using NanoBiT

For the KRAS(G12D):CRAF-RBD NanoBiT interaction assay, cytomegalovirus-based expression constructs were made encoding fusions of LgBiT to KRAS 4B (UniProt P01116-2) with the G12D mutation and SmBiT to residues 51−133 of CRAF (UniProt P04049-1, CRAF(RBD)). HEK293 cells (~4 × 10^6^) were transiently transfected in T75 flasks with plasmids encoding a LgBiT-KRAS(G12D) and SmBiT-CRAF(RBD). Plasmids were transfected at 500 ng per construct per flask together with 9 µg of Transfection Carrier DNA (Promega) at a 3:1 lipid:DNA ratio using FuGENE HD (10 ml total volume). Following expression for 24 h, cells were plated at 20,000 cells per well in Opti-MEM I (Thermo) containing 4% FBS and allowed to attach overnight. Serial dilutions of MRTX-EX185 were made in Opti-MEM I containing 4% FBS and 1× Vivazine substrate (Promega N2581) to generate 1× solutions containing varying concentrations of MRTX-EX185. Existing medium was removed by plate inversion and blotting, and 1× solutions were added to respective wells. Luminescence was measured every 5 min in a GloMax Discover luminometer at 37 °C for 16 h using a 1 s integration time.

### Measurements of antiproliferative activity in cells

Antiproliferative activity was measured as a decrease in cellular ATP levels using the CellTiter-Glo 2.0 assay (Promega) according to the manufacturer’s protocols. HEK293 were seeded at 2,500 cells per well and all other cell lines were seeded at 5,000 cells per well. Clear monophasic behavior was fitted to equation (2) to interpolate the antiproliferative potency (IC_50_). To fit the biphasic antiproliferative behavior of MRTX849 in H358 and MIA PaCa-2 cells, the data were fit to a biphasic inhibitor model with variable Hill slope (equation 3) below

3 $$\begin{array}{l}Y = {\rm{Bottom}} + ({\rm{Top}} - {\rm{Bottom}}) \times Frac/\left( {1 + ({\rm{IC}}50_{1/X})^{nH1}} \right)\\\qquad + ({\rm{Top}} - {\rm{Bottom}}) \times (1 - Frac)/\left( {1 + ({\rm{IC}}50_{2/X})^{nH2}} \right)\end{array}$$

### Inhibition of ERK phosphorylation

Cellular ERK phosphorylation (Thr202/Tyr204) was quantified using a p-ERK (Thr202/Tyr204) cellular kit (Cisbio). SW-1990 cells (1 × 10^6^ cells per ml, 50 µl per well) were plated in cell culture medium (DMEM (Gibco) + 10% FBS (Seradigm)) 12 h before the experiments. On the day of treatment, serially diluted 2× solutions were prepared in cell culture medium (9 + 1 points, 3:1 dilution starting from 60 µM, DMSO 0.6%). Cells in each well were treated with 2× small-molecule solutions (50 µl), after which they were incubated for 4 h at ambient temperature. Media were removed by aspiration. Cells were lysed with 50 µl of supplemented lysis buffer 1× (Cisbio) for 30 min at ambient temperature. Lysates were homogenized and transferred (16 µl) to a low-volume 384-well detection plate (Corning 4513). Premixed p-ERK antibody solutions (4 µl, Advanced p-ERK1/2 d2 Ab 20× (19×) + Advanced p-ERK1/2 Eu Cryptate Ab 20× (19×) + detection buffer; Cisbio) were added to each well of the plates. The mixtures were incubated at ambient temperature for 4 h before reading on a TECAN plate reader using the TR FRET mode with 60-µs lag time and 500-µs integration time. The ratio of the acceptor and donor emission signals for each individual well was calculated by

$${{{\mathrm{TR}}}}\;{{{\mathrm{FRET}}}}\;{\rm{ratio}} = \left[ {{\rm{Signal}}\;665\;{\rm{nm}}} \right]/\left[ {{\rm{Signal}}\;620\;{\rm{nm}}} \right] \times 10,000$$

Total ERK data were acquired via the same procedure using the Total ERK cellular kit (Cisbio) on the same cell lysates. For each marker (p-ERK or total-ERK), the TR FRET ratio was normalized to the respective DMSO control. The ratio of p-ERK over total-ERK was calculated and fit to equation (2) above.

### Induction of ERK phosphorylation by expression of KRAS constructs

The impact of expressing untagged or NanoBit-tagged KRAS variants on ERK phosphorylation was evaluated in HEK293 cells overexpressing an ERK1 substrate protein using AlphaLisa SureFire Ultra p-ERK1/2 (Thr202/Tyr204) and AlphaLisa SureFire Total ERK 1/2 assays (PerkinElmer). The ERK1 substrate protein was NanoLuc-ERK1 (NanoLuc-MAPK3; Promega), which was coexpressed with the RAS vectors to create a uniform total-ERK level across various transfection conditions and minimize the potential impact of overexpressing KRAS on endogenous ERK levels. Moreover, the NanoLuc tag on the ERK1 substrate allowed total luminescence to be used as an independent method to ensure uniform expression across samples. HEK293 cells were transfected as described above for cellular RAS target engagement assays, except that the KRAS constructs, NanoLuc-ERK1 and Transfection Carrier DNA were combined at a mass ratio of 2:1:7 or as indicated in the figures. Cells were plated directly into white 96-well tissue culture-treated assay plates (Corning) at 2 × 10^5^ cells per ml (100 µl total volume) and allowed to express overnight.

For p-ERK and total-ERK measurements, the medium was aspirated from the assay wells, after which 62 µl of AlphaLisa lysis buffer was added to the cells and the plates were agitated for 10 min at 350 r.p.m. Cell lysates thus prepared were then analyzed using the AlphaLisa SureFire Ultra assays above according to the manufacturer’s instructions. Fluorescence emission measurements at 615 nm were recorded on a BMG CLARIOstar instrument using the AlphaLisa protocol. Raw fluorescence values from p-ERK measurements were normalized to the raw fluorescence values from the total-ERK measurements from the same lysate samples to produce the p-ERK/total-ERK ratio that was used for comparison. The p-ERK or total-ERK positive control lysates provided with the AlphaLisa SureFire Ultra kits were diluted 1:1 with lysis buffer before analysis.

Total luminescence was measured in the NanoLuc-ERK1 expressing cell samples as described above for cellular RAS target engagement measurements, except that the Nano-Glo protocol (unfiltered luminescence) was used on the GloMax Discover instrument.

### Preparation of U-^15^N Ras proteins

The plasmids for bacterial expression of HRAS 1−166 (WT; His-TEV-N; pProEx; ampicillin resistance) and KRAS 1−169 (WT, G12C and G12D; His-TEV-N; pJ411; kanamycin resistance) have been previously published^2^. BL21(DE3) competent cells were transformed with 1–2 ng of plasmid, and cultures were grown at 37 °C in M9 minimal media containing 1 g L^−1^ of ^15^N ammonium chloride (99%; Cambridge Isotope Laboratories) and the appropriate antibiotic (0.1 mg ml^−1^ of carbenicillin or 0.1 mM kanamycin). Protein expression was induced after cooling the cultures to 18 °C (*A*_600_ = 0.4–0.6) by adding 1.0 mM IPTG, and the flasks were shaken at 200 r.p.m. overnight. Procedures for lysis and purification were followed as previously published^2^. Nucleotide exchange from GDP to GPPNHP (Jena Biosciences) was performed before the final gel-filtration purification and according to a published procedure comprising EDTA-mediated exchange, desalting and cleavage of residual GDP with an alkaline phosphatase (CIP or Quick CIP; New England Biolabs)^2,39–41^. Final gel-filtration purification was performed on a Superdex 75 column (GE) with storage buffer. Proteins were concentrated to 0.5–1 mM (Amicon Ultra-4, 10k MWCO, EMD), concentrations were determined by UV absorbance (ε = 13,410 M^−1^cm^−1^ for HRAS 1−166 and 11,920 M^−1^cm^−1^ for KRAS 1−169), and aliquots were flash frozen with liquid N_2_ and stored at −80 °C.

Storage buffer: 40 mM HEPES, 150 mM NaCl, 4 mM MgCl_2_, 5% glycerol, 7% D_2_O. Titrated to pH 7.4 with NaOH.

### Preparation of ^15^N-labeled KRAS(G12C)-MRTX849

A 4-ml 0.10 mM sample of U-^15^N KRAS(G12C)−GDP 1−169 (*M*_r_ = 19,579) was reserved before the final gel-filtration purification step. The concentration was determined by a bicinchoninic acid assay (Pierce, ThermoFisher Scientific) relative to a BSA standard. A solution of MRTX849 (120 µl, 10 mM in DMSO, 3.0 eq.) was added, and the mixture was rotated at ambient temperature for 15 min, then concentrated (Amicon Ultra-4, 10k MWCO, EMD) and purified by gel filtration (SD75, GE) as described above. The purified protein was analyzed by LC/MS to ensure complete conversion to the 1:1 protein–ligand adduct (*M*_r_ = 20182) and the concentration determined by a BCA assay (Pierce, ThermoFisher Scientific).

### ^1^H−^15^N HSQC NMR sample preparation and acquisition

General procedure: a 0.030-µmol aliquot of U-^15^N protein in storage buffer was diluted to 255 µl with HSQC NMR sample buffer on ice. Sodium trimethylsilylpropanesulfonate (DSS) (15 µl, 20 mM in buffer) and the small-molecule ligand (30 µl, 2.0 mM in dmso-*d*_6_) were added, the sample was mixed by vortex, and the resulting solution was transferred to a 5-mm D_2_O-matched Shigemi NMR tube (BMS-3). The final concentration of protein and ligand were 100 and 200 µM, respectively. One-dimensional (1D) ^1^H (ZGESGP) and two-dimensional (2D) ^1^H−^15^N fast HSQC (FHSQCCF3GPPH, ns = 8, tdf1 = 256, GARP decoupling) spectra were recorded on an 800 MHz Bruker Avance spectrometer at 298 K. For any case in which strong CSPs were observed, a second sample containing the same protein−ligand combination at 100 and 30 µM, respectively was also prepared, and spectra were acquired under the same conditions. For the mixed samples containing both nucleotide states, the scan number was doubled (ns = 16) to improve the signal-to-noise ratio. Some spectra were also acquired with 0, 5 and 10% dmso-*d*_6_ and/or with minor adjustments to pH and temperature for comparison to previously published assignments.

HSQC NMR buffer: 40 mM HEPES, 150 mM NaCl, 4 mM MgCl_2_, 7% D_2_O. Titrated to pH 7.4 with NaOH.

### ^1^H−^15^N−^1^H NOESY-HSQC NMR sample preparation and acquisition

A 0.150-µmol sample of U-^15^N KRAS(G12C)−GDP−MRTX849 protein in storage buffer was diluted to 400 µl with NOESY NMR buffer on ice. The buffer was exchanged to the NOESY NMR buffer with a desalting column (5 ml HiTrap Desalting, Cytiva and AKTA FPLC, GE). The protein-containing fractions were combined (1.5 ml), concentrated to 0.3 ml (10k MWCO Amicon Ultra-4, EMD), transferred to a 5-mm Shigemi NMR tube (BMS-3) and gently sparged with Ar before sealing with parafilm (final concentration ~0.5 mM). 1D ^1^H (ZGESGP), 2D ^1^H−^15^N fast HSQC (FHSQCCF3GPPH, ns=8, tdf1 = 256, GARP decoupling), and three-dimensional (3D) ^1^H−^15^N−^1^H NOESY-HSQC (NOESYHSQCF3GPWG3D, ns = 16, tdf1 = 128, tdf2 = 40, GARP decoupling, 120 ms mixing time) NMR spectra were recorded on an 800 MHz Bruker Avance spectrometer at 298 K. A second 2D HSQC spectrum was acquired after the 3D NOESY-HSQC experiment with identical parameters to confirm sample stability during the 28 h acquisition.

A 0.374-µmol sample of U-^15^N KRAS(G12D)−GDP in protein buffer was diluted with NOESY NMR buffer, and the buffer was exchanged as described above. The concentration of the resulting 1.5-ml solution was determined to be 0.20 ± 0.03 mM by a BCA assay (Pierce, ThermoFisher Scientific); and this solution was concentrated to 0.50 ml (0.60 mM). A 200-µl aliquot (0.12 µmol) of this solution was diluted to 294 µl with the same buffer, and MRTX849 or EX185 (6 µl, 20 mM in dmso-*d*_6_) was added. The samples were prepared as described above, and the same series of spectra were acquired. The remainder of the protein solution (100 µl, 0.060 µmol) was diluted to 294 µl with the same buffer, dmso-*d*_6_ (6 µl) was added, and 1D ^1^H and 2D fast ^1^H−^15^N HSQC spectra were acquired from this sample for comparison.

NOESY NMR buffer: 20 mM sodium phosphate, 140 mM NaCl, 15 mM MgCl_2_, 10 mM EDTA, 3 mM NaN_3_, 1 mM GDP, 1 mM DSS, 10% D_2_O. Titrated to pH 7.4 with NaOH.

### NMR data analysis

Spectra were analyzed with Bruker Topspin 4.0, CCPNMR Analysis v.3 (ref. ^42^) and/or MestReNova v14. ^1^H chemical shifts were referenced to 1 mM DSS at 0 ppm; ^15^N chemical shifts were referenced indirectly with Ξ = 0.101329118. The spectra images were created with CCPNMR Analysis v.3. Full spectra of each protein–ligand combination (red) superimposed with the dmso-*d*_6_ control (blue) are shown in Supplementary Note 1. Well-resolved peaks of the unbound proteins were assigned by comparison with data imported from the BMRB. For the protein–ligand combinations identified as strongly binding, many peaks of the protein–ligand complexes exhibited large CSPs, and no chemical exchange between bound and unbound proteins was observed; therefore, very few peaks in the HSQC spectra of these protein−ligand complexes could be confidently assigned based solely on this data. 3D NOESY-HSQC data were analyzed with CCPNMR Analysis v.3. Sequential backbone NH ^1^H and ^15^N shifts were identified by mutual NOESY crosspeaks. Segments belonging to the P-loop (13−20), SI (35−38), SII (73−77) and α4 (128−135) were assigned from this data, and CSPs were calculated by comparison of this data with data from spectra of the unbound protein acquired under the same conditions.

BMRB data used for reference:

HRAS−GDP 1−166: entry 18479 (ref. ^43^).

HRAS−GPPNHP 1−166: entry 17678 (ref. ^43^).

KRAS−GDP 1−169: entry 27720 (ref. ^44^).

KRAS(G12C)−GDP 1−169: entry 27646 (ref. ^44^).

KRAS(G12D)−GDP 1−169: entry 27719 (ref. ^44^).

### Quantification and statistical analysis

Data from multiple independent experiments (*n*) are presented as mean values ± s.e.m. and data involving technical replicates are presented as mean ± s.d. as indicated in the figure captions. The number of experimental or technical replicates for each experiment is also described in each individual figure caption. Apparent affinity values were determined using the sigmoidal dose−response (variable slope) equation available in GraphPad Prism (v.8).

### Reporting Summary

Further information on research design is available in the Nature Research Reporting Summary linked to this article.

## Online content

Any methods, additional references, Nature Research reporting summaries, source data, extended data, supplementary information, acknowledgements, peer review information; details of author contributions and competing interests; and statements of data and code availability are available at 10.1038/s41589-022-00985-w.

## Supplementary information

Supplementary Information Supplementary Table 1, Figs. 1–12, and Notes 1 and 2.

Reporting Summary

Supplementary Data 1 Source Data for Supplementary Figures

## Data Availability

Source data are provided with this paper. The authors declare that the data supporting the findings of this study are available within the article, the accompanying Source Data, the Supplementary Information and the Supplementary Data. Additional information, resources and reagents will be made available upon reasonable request; requests should be directed to and will be fulfilled by the lead contact M.B.R.

## Source data

Source Data Fig. 2 Statistical source data.
Source Data Fig. 3 Statistical source data.
Source Data Fig. 4 Statistical source data.
Source Data Fig. 5 Statistical source data.
Source Data Fig. 6 Statistical source data.
