# Supplementary material for: KRAS is vulnerable to reversible switch-II pocket engagement in cells
Source: Nat Chem Biol. 2022 Mar 21;18(6):596–604. doi: 10.1038/s41589-022-00985-w (PMC9135634; doi:10.1038/s41589-022-00985-w)
Supplement: Supplementary file 1 — Supplementary Table 1, Figs. 1–12, and Notes 1 and 2. [file 41589_2022_985_MOESM1_ESM.pdf]

nature portfolio

https://doi.org/10.1038/s41589-022-00985-w

Supplementary information

KRAS is vulnerable to reversible switch-II pocket engagement in cells

In the format provided by the authors and unedited

Supporting Information for: KRAS is vulnerable to reversible switch-II pocket engagement in cells

James D. Vasta[1,3], D. Matthew Peacock[2,3], Qinheng Zheng[2,3], Joel A. Walker[1], Ziyang Zhang[2], Chad A. Zimprich[1], Morgan R. Thomas[1], Michael T. Beck[1], Brock F. Binkowski[1], Cesear R. Corona[1], Matthew B. Robers[1]*, Kevan M. Shokat[2]*

1. Promega Corporation. 2800 Woods Hollow Road, Madison, WI, 53719, USA

2. Department of Cellular and Molecular Pharmacology, Howard Hughes Medical Institute, University of California San Francisco, San Francisco, California 94158, USA

3. These authors contributed equally

* Corresponding Authors

Table of Contents:

Supplementary Tables and Figures 2-20

Supplementary Table 1 2

Supplementary Figure 1 3

Supplementary Figure 2 4

Supplementary Figure 3 5

Supplementary Figure 4 6

Supplementary Figure 5 7

Supplementary Figure 6 9

Supplementary Figure 7 11

Supplementary Figure 8 12

Supplementary Figure 9 14

Supplementary Figure 10 16

Supplementary Figure 11 18

Supplementary Figure 12 20

Supplementary Note 1 – HSQC NMR spectra 21-50

Effect of ligands on HSQC spectra 21-44

Slow k_{off} of ligand binding determined from HSQC spectra of sub-stoichiometrically ligated proteins 45

Partial reassignments of HSQC spectra on the basis of 3D NOESY-HSQC correlations 46-48

Nucleotide state selectivity of MRTX-EX185 49-50

Supplementary Note 2 – Chemical synthesis and characterization 51-81

Synthesis of pan RAS BRET probe (**7**) 52

Preparation of piperazine precursor of MRTX849 59

Preparation of saturated amide analogs of MRTX849 (**9-14**) 65

Preparation of MRTX-EX185 (**6**) 71

References 81

Compound	KRAS WT	KRAS (G12C)	KRAS (G12D)	KRAS (G12V)	KRAS (G13D)	KRAS (Q61H)	KRAS (Q61L)	KRAS (Q61R)	HRAS WT
ARS-1620	N/D[b]	210 ± 20	N/D[b]	N/D[b]	N/D[b]	N/D[b]	N/D[b]	N/D[b]	N/D[b]
AMG510	No Binding[c]	30 ± 10	No Binding[c]	No Binding[c]	No Binding[c]	No Binding[c]	No Binding[c]	No Binding[c]	No Binding[c]
MRTX849	600 ± 100	3.3 ± 0.9	3900 ± 600	No Binding[c]	1500 ± 300	1000 ± 200	2000 ± 400	No Binding[c]	No Binding[c]
MRTX1257	620 ± 30	1.9 ± 0.3	5000 ± 1000	No Binding[c]	1100 ± 200	700 ± 100	1370 ± 90	No Binding[c]	No Binding[c]
**9**	750 ± 60	Weak[d]	Weak[d]	Weak[d]	1060 ± 50	1200 ± 100	1200 ± 200	No Binding[c]	No Binding[c]
**10**	590 ± 80	Weak[d]	Weak[d]	Weak[d]	1130 ± 20	1200 ± 200	1700 ± 100	No Binding[c]	No Binding[c]
**11**	Weak[d]	No Binding[c]	No Binding[c]	No Binding[c]	Weak[d]	Weak[d]	Weak[d]	No Binding[c]	No Binding[c]
**12**	1500 ± 300	Weak[d]	Weak[d]	Weak[d]	3300 ± 400	3300 ± 600	3200 ± 100	Weak[d]	No Binding[c]
**13**	3200 ± 1000	No Binding[c]	No Binding[c]	No Binding[c]	6000 ± 2000	7000 ± 3000	Weak[d]	No Binding[c]	No Binding[c]
**14**	410 ± 90	3000 ± 800	Weak[d]	Weak[d]	820 ± 40	840 ± 30	690 ± 90	Weak[d]	No Binding[c]
MRTX-EX185	110 ± 50	290[e]	90 ± 20	700 ± 200	240[e]	130[e]	290[e]	1400[e]	Weak[d]

Supplementary Table 1. Engagement potency values (IC_{50}, nM)[a] for SII-P binders across RAS variants in HEK-293 cells using the BRET assay. [a]IC_{50} values are the mean ± S.E. of at least three independent experiments, collected after a 2 h incubation. [b]Not determined. [c]No Binding indicates that the normalized BRET at the top dose was > 75%. [d]Weak binding indicates that the normalized BRET at the top dose was incomplete but ≤ 75%. [e]IC_{50} values are from a single biological replicate with 4 technical replicates.

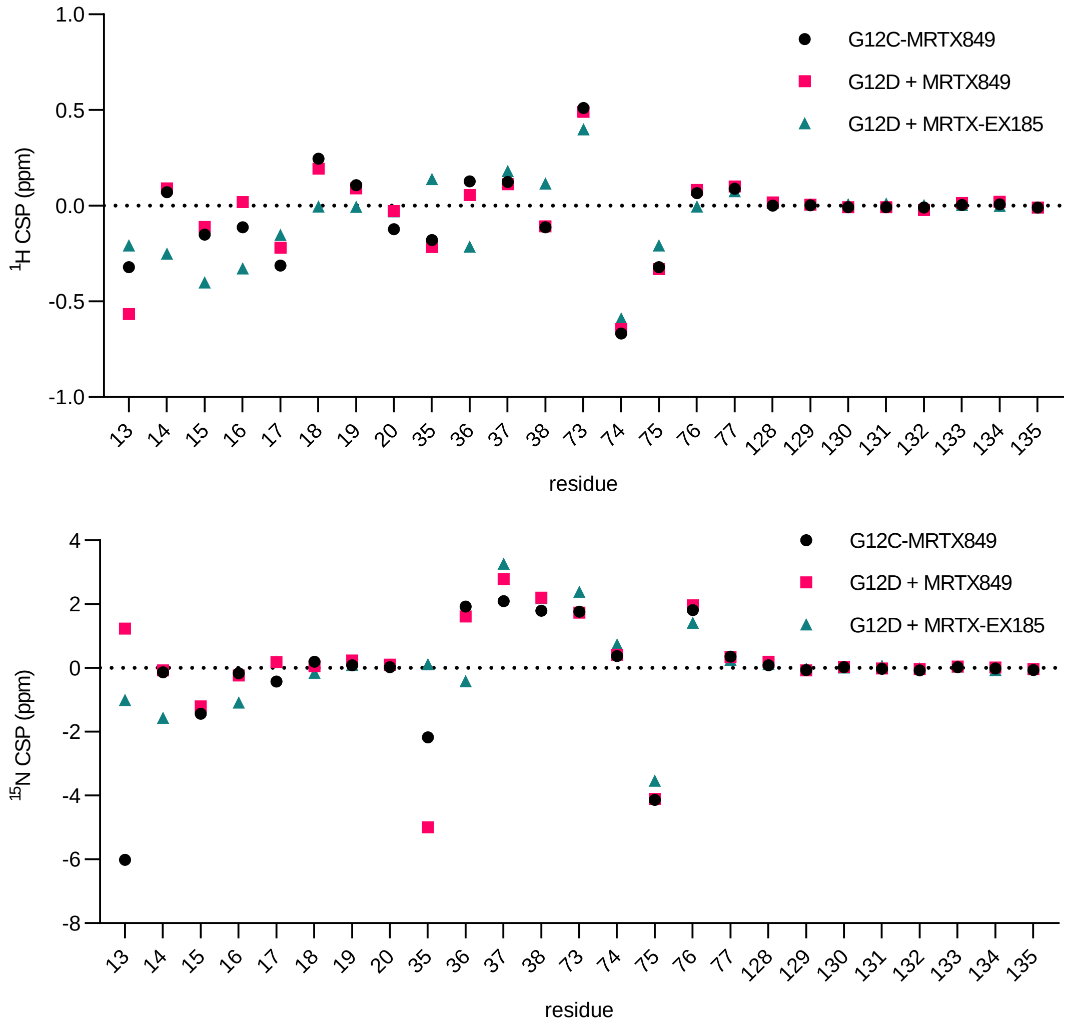

Supplementary Figure 1. Effects of MRTX849 and MRTX-EX185 on the HSQC NMR spectra of U-^{15}N KRAS(G12C)-GDP and KRAS(G12D)-GDP. The HSQC NMR spectra of protein-ligand complexes were partially assigned on the basis of mutual ^{1}H NOEs. Segments from the P-loop, SI, and SII show significant CSPs in both ^{1}H (top) and ^{15}N (bottom). An unperturbed segment (α4) is included for comparison. Spectra recorded at pH 7.4 and 298 K with 0% (G12C) or 2% (G12D) dmso-d_6.

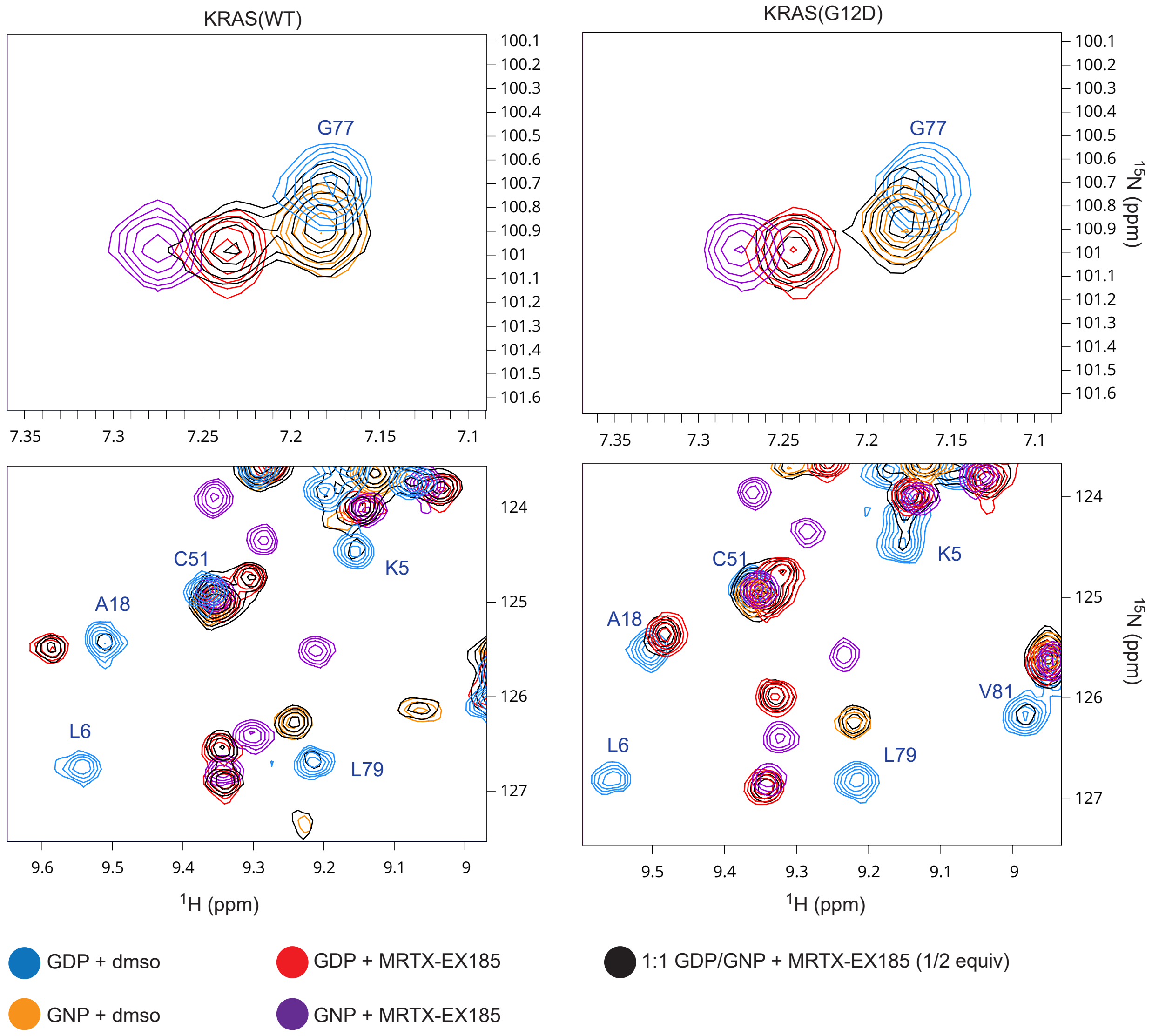

Supplementary Figure 2. KRAS nucleotide state preference of MRTX-EX185. Superimposed HSQC NMR spectra of 100 μM WT (left) and G12D (right) U-^{15}N KRAS-GDP 1-169 with (red) and without (blue) 200 μM MRTX-EX185, 100 μM U-^{15}N KRAS-GNP 1-169 with (purple) and without (orange) 200 μM MRTX-EX185, and a 1:1 mixture of the two nucleotide states (100 μM each) with 50 μM MRTX-EX185 (black). Spectra recorded at pH 7.4 and 298 K with 10% dmso-d_6. Regions chosen to show examples of peaks that are well-resolved between the four possible complexes. No well-resolved peaks of MRTX-EX185-KRAS-GNP in the mixed samples could be detected above the noise limit of the spectra (noise <10% of peak heights for most peaks). Assignments of KRAS-GDP are noted in blue text (BMRB 27720 for WT and 27719 for G12D).

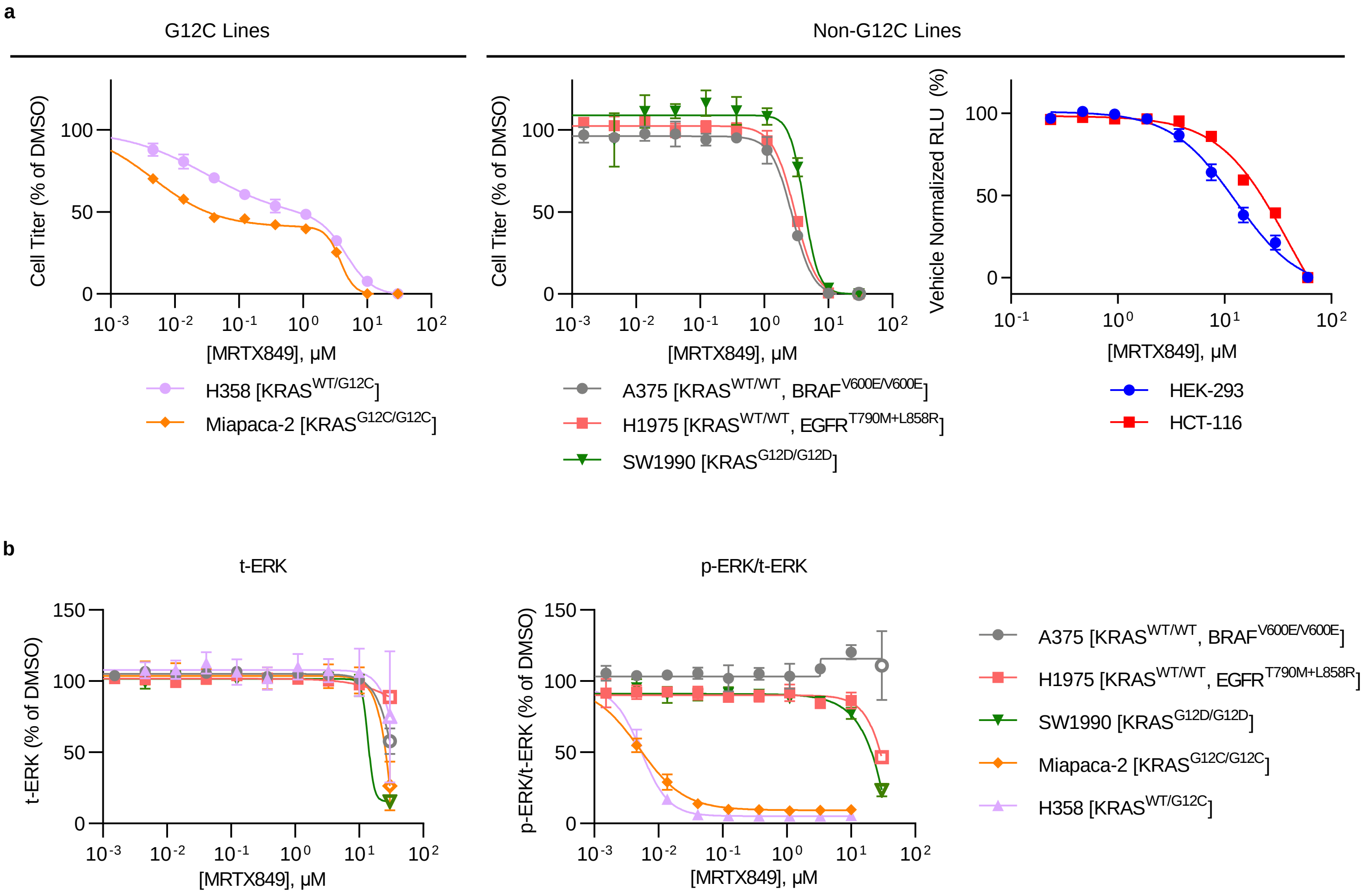

Supplementary Figure 3. Characterization of the antiproliferative effects of MRTX849. a. Cell growth of KRAS(G12C) (left) and non-KRAS(G12C) cell lines (right) in the presence of MRTX849 evaluated using CellTiter-Glo. For KRAS(G12C) lines, the curve is fitted to a biphasic inhibitory model with variable hill slopes; for non-KRAS(G12C) lines, the curve is fitted to a monophasic inhibitory model with a variable hill slope. For H358, Miapaca-2, A375, H1975, and SW1990 cells, data are mean of at least 3 technical replicates ± S.D. (n=1). For HEK-293 and HCT-116 cells, data are the mean of technical duplicates, (n=1). **b.** Effect of MRTX849 on phosphorylation of ERK. Phospho-ERK (p-ERK) normalized to total ERK (t-ERK) data across 5 different cell lines (right). Hollowed data points represent concentrations over which cytotoxic effects are observed as a reduction in t-ERK levels (left). Data are the mean of technical triplicates (n=1).

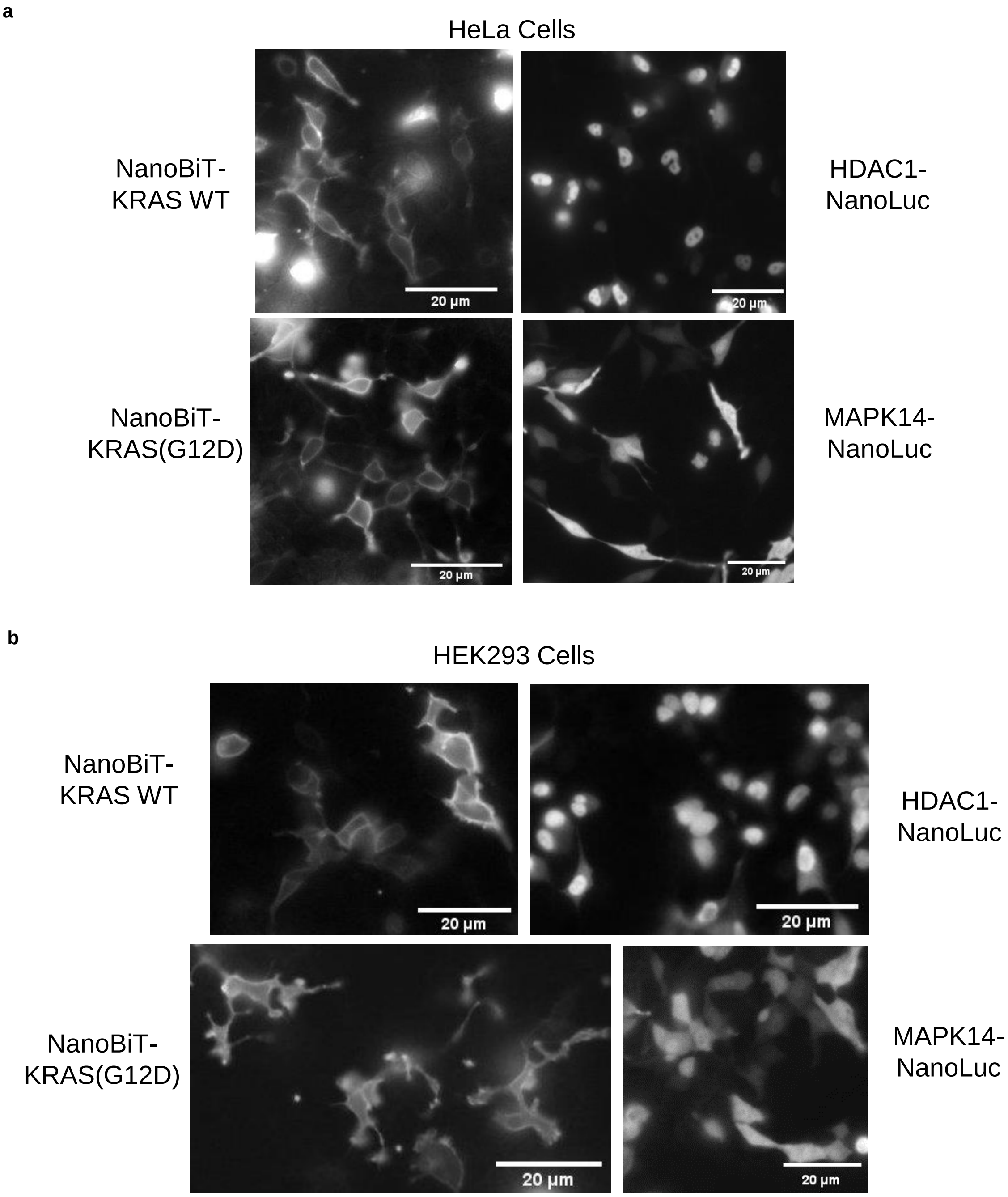

Supplementary Figure 4. Bioluminescence imaging of homodimeric NanoBiT-RAS complexes in cells. The N-terminus of each KRAS4B variant was tagged with complementation-based NanoBiT (SmBiT and LgBiT) reporters (Wells et al.[20], Dixon et al.[21]). Full-length RAS constructs with intact C-termini were utilized to preserve lipidation via the native membrane localization domain. Co-expression of full length LgBiT-RAS with SmBiT-RAS generated a luminescent signal selectively at homo-multimeric RAS compexes in cells. Bioluminescence imaging was performed as described in the materials and methods section. Scale bars represent 20 µM. **a.** Imaging of HeLa cells expressing membrane localized NanoBiT-KRAS WT and NanoBiT-KRAS(G12D), as well as nuclear localized HDAC1-NanoLuc, and cytosol localized MAPK14-NanoLuc. **b.** Imaging of HEK293 cells expressing membrane localized NanoBiT-KRAS WT and NanoBiT-KRAS(G12D), as well as nuclear localized HDAC1-NanoLuc, and cytosol localized MAPK14-NanoLuc. Images are representative of experiments performed twice (n=2).

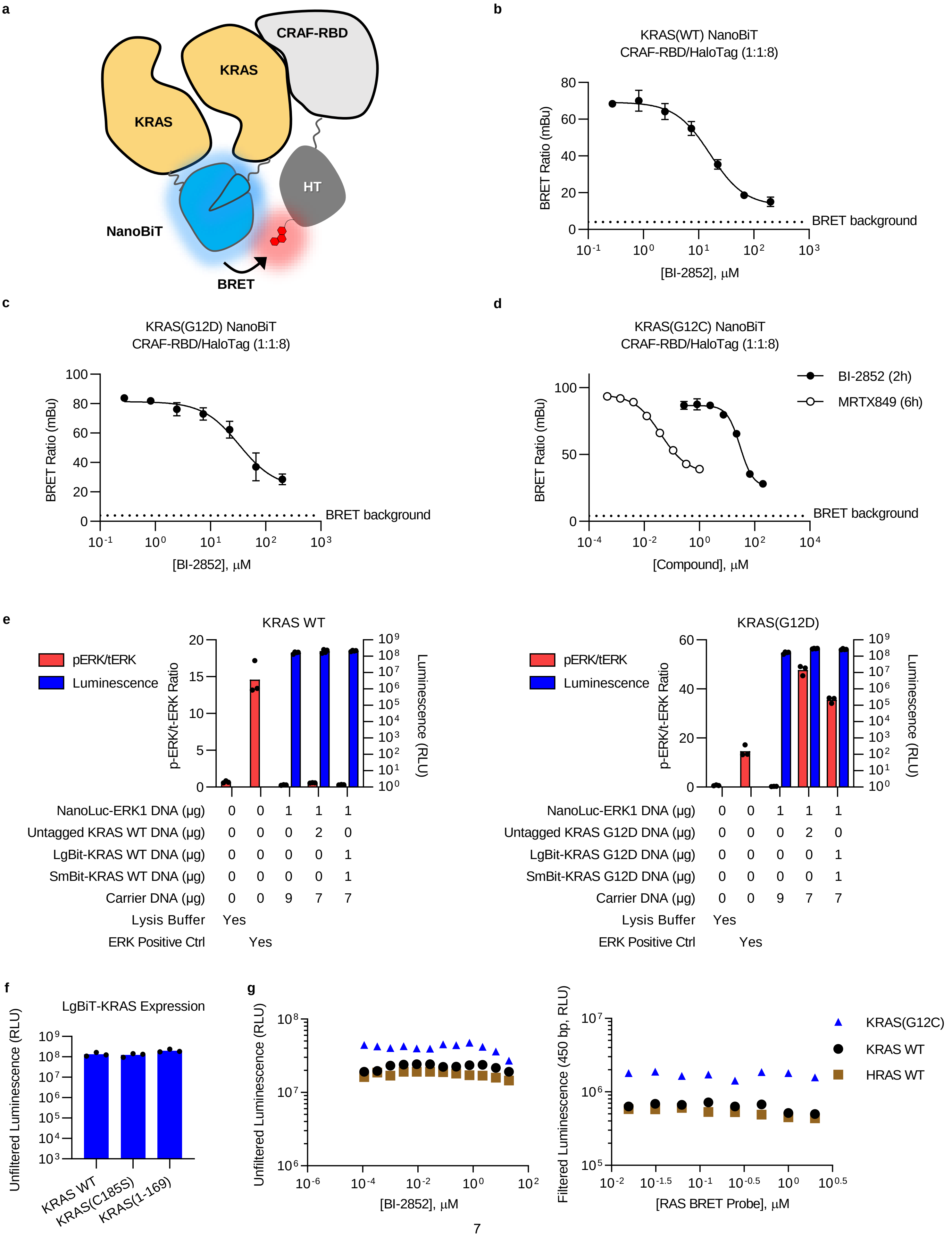
a
b
c
d
e
f
g
CRAF-RBD
KRAS
KRAS
HT
NanoBiT
BRET
KRAS(WT) NanoBiT
CRAF-RBD/HaloTag (1:1:8)
BRET Ratio (mBu)
BRET background
[BI-2852], µM
KRAS(G12D) NanoBiT
CRAF-RBD/HaloTag (1:1:8)
KRAS(G12C) NanoBiT
CRAF-RBD/HaloTag (1:1:8)
BI-2852 (2h)
MRTX849 (6h)
[Compound], µM
KRAS WT
KRAS(G12D)
pERK/tERK
Luminescence
p-ERK/t-ERK Ratio
Luminescence (RLU)
NanoLuc-ERK1 DNA (µg) 0 0 1 1 1
Untagged KRAS WT DNA (µg) 0 0 0 2 0
LgBit-KRAS WT DNA (µg) 0 0 0 0 1
SmBit-KRAS WT DNA (µg) 0 0 0 0 1
Carrier DNA (µg) 0 0 9 7 7
Lysis Buffer Yes
ERK Positive Ctrl Yes
NanoLuc-ERK1 DNA (µg) 0 0 1 1 1
Untagged KRAS G12D DNA (µg) 0 0 0 2 0
LgBit-KRAS G12D DNA (µg) 0 0 0 0 1
SmBit-KRAS G12D DNA (µg) 0 0 0 0 1
Carrier DNA (µg) 0 0 9 7 7
Lysis Buffer Yes
ERK Positive Ctrl Yes
LgBiT-KRAS Expression
Unfiltered Luminescence (RLU)
KRAS WT
KRAS(C185S)
KRAS(1-169)
Filtered Luminescence (450 bp, RLU)
[RAS BRET Probe], µM
KRAS(G12C)
KRAS WT
HRAS WT

Supplementary Figure 5. Characterization of homodimeric NanoBiT-RAS complexes in cells. a. Schematic representation of the NanoBiT-KRAS : CRAF(RBD)-HaloTag NanoBRET protein-protein interaction assay approach. Co-expression of SmBiT-KRAS and LgBiT-KRAS constructs in cells produces luminescence via the NanoBiT reporter. Interaction of the CRAF(RBD)-HaloTag construct with the NanoBiT-KRAS complex produces BRET, and compounds that inhibit the KRAS : CRAF(RBD) interaction compete the BRET signal. **b−d.** Dose-dependent inhibition of the NanoBiT-KRAS : CRAF(RBD) interaction in live HEK293 cells for KRAS WT, KRAS(G12D), and KRAS(G12C). Individual data points are the mean ± S.D. of 4 technical replicates (n=1). **e.** NanoLuc-ERK1 phosphorylation assays in HEK293 cells grown in low serum (1% FBS) expressing KRAS WT (left) or KRAS(G12D) (right) constructs. KRAS WT constructs did not induce phosphorylation of ERK, whereas NanoBiT-KRAS(G12D) induced ERK phosphorylation similar to that of untagged KRAS(G12D). ERK1 phosphorylation datapoints are the mean ± S.D. of 3 technical replicates (n=1). Total Luminescence control datapoints are the mean ± S.D. of 4 technical replicates (n=1). **f.** Removal of the HVR or mutation at C185S does not affect the expression level of the LgBiT-KRAS fusions in HEK293 cells as measured by detection with HiBiT peptide after lysis. Results are mean ± S.E. of 3 independent experiments (n=3). **g.** Effect of BI-2852 (left) and the BRET probe (right) on luminescent signal for RAS variants in HEK293 cells. Results are technical singlicates (n=1).

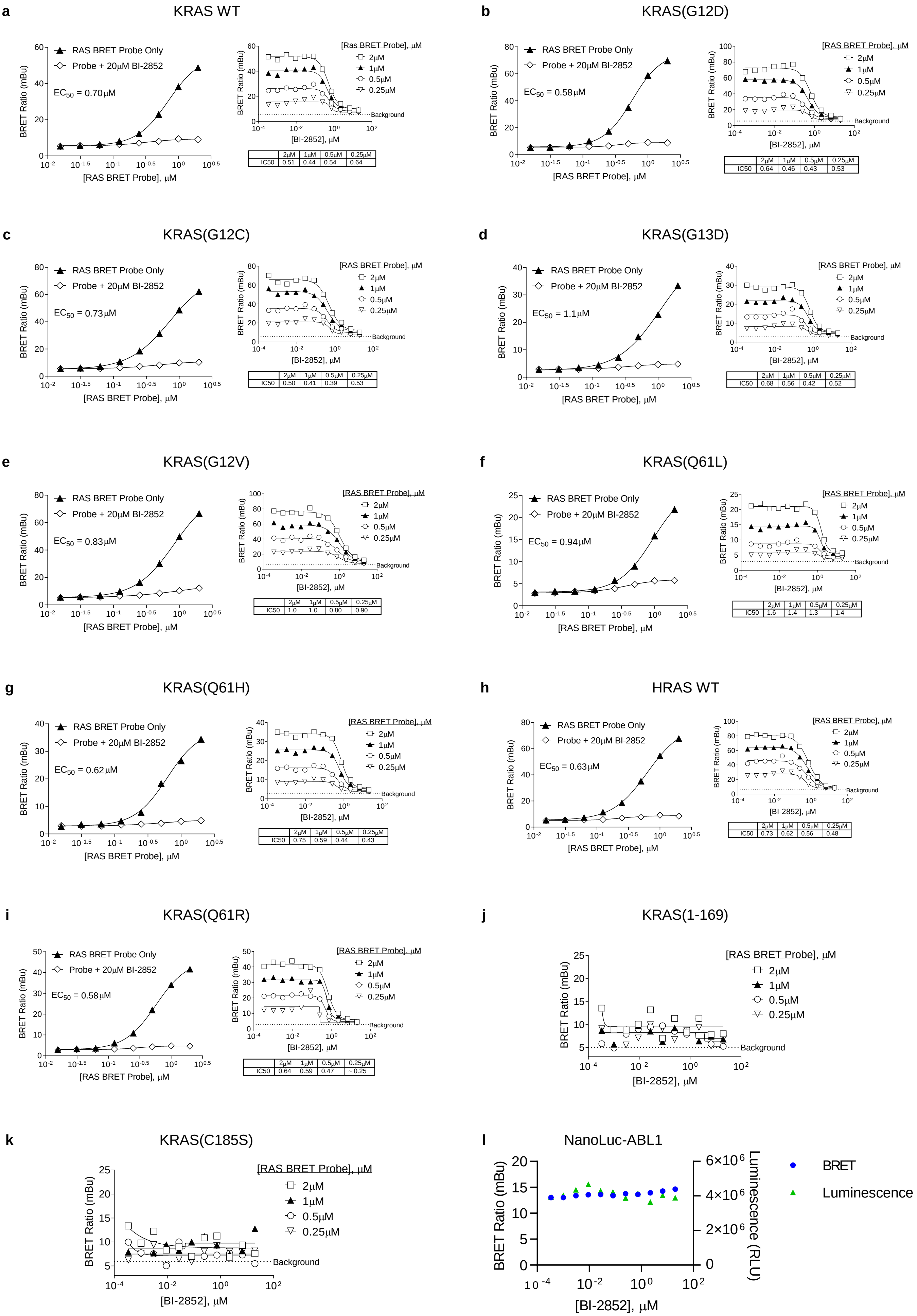

a) KRAS WT

	2μM	1μM	0.5μM	0.25μM
IC50	0.51	0.44	0.54	0.64

b) KRAS(G12D)

	2μM	1μM	0.5μM	0.25μM
IC50	0.64	0.46	0.43	0.53

c) KRAS(G12C)

	2μM	1μM	0.5μM	0.25μM
IC50	0.50	0.41	0.39	0.53

d) KRAS(G13D)

	2μM	1μM	0.5μM	0.25μM
IC50	0.68	0.56	0.42	0.52

e) KRAS(G12V)

	2μM	1μM	0.5μM	0.25μM
IC50	1.0	1.0	0.80	0.90

f) KRAS(Q61L)

	2μM	1μM	0.5μM	0.25μM
IC50	1.6	1.4	1.3	1.4

g) KRAS(Q61H)

	2μM	1μM	0.5μM	0.25μM
IC50	0.75	0.59	0.44	0.43

h) HRAS WT

	2μM	1μM	0.5μM	0.25μM
IC50	0.73	0.62	0.56	0.48

i) KRAS(Q61R)

	2μM	1μM	0.5μM	0.25μM
IC50	0.64	0.59	0.47	~ 0.25

Supplementary Figure 6. Characterization of SI/II-P BRET assay for RAS in live cells. The N-terminus of each HRAS or KRAS4B variant was tagged with complementation-based NanoBiT (SmBiT and LgBiT) reporters (Wells et al.[20], Dixon et al.[21]) Full-length RAS constructs with intact C-termini were utilized to preserve lipidation via the native membrane localization domain. Co-expression of full length LgBiT-RAS with SmBiT-RAS generated a luminescent signal selectively at homo-multimeric RAS compexes in cells. **a-i.** Characterization of BRET between RAS dimers and the RAS BRET probe for wildtype KRAS and HRAS, as well as KRAS hotspot mutants. Titration of BI-2852 confirms specific BRET in live cells (n=1). **j,k.** Mutations to critical lipidation sites on KRAS are essential for the BRET signal with the SI/II-P BRET probe (n=1). Instrument background BRET values are indicated by the dashed line marked “background”. **l.** BI-2852 had no effect on the BRET or luminescence in a NanoBRET target engagement assay for NanoLuc-ABL1, further confirming the specificity of the competitive effects observed in the RAS assays. Data points are the mean of technical duplicates (n=1).

a

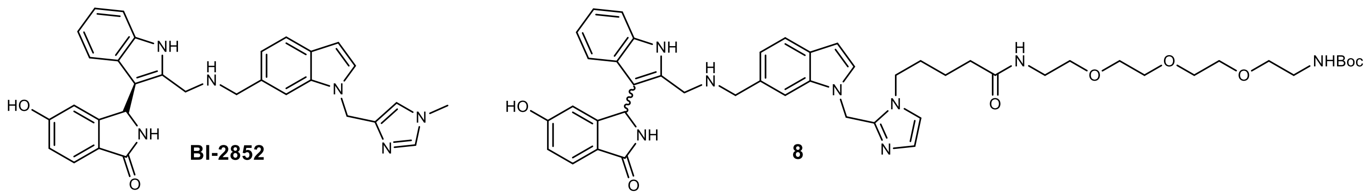

b

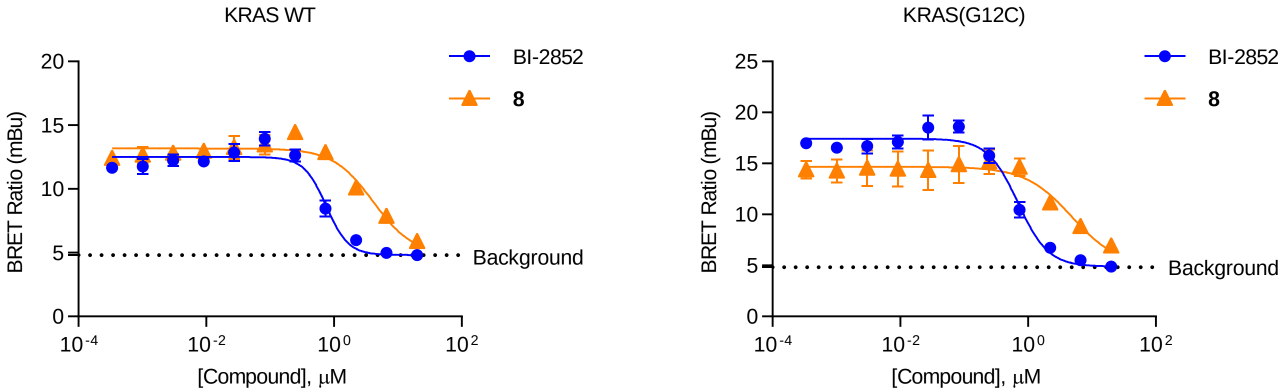

	BI-2852	**8**
IC50	0.76	4.1

	BI-2852	**8**
IC50	0.70	4.8

Supplementary Figure 7. Potency of linker-derivatized BI-2852 analog (8). a. Structures of BI-2852 vs a linker-derivatized RAS BRET probe precursor (**8**). **b. 8** shows weaker potency of live cell target engagement compared to underivatized BI-2852 across both KRAS WT and KRAS(G12C). Data are the mean ± S.D. of 4 technical replicates (n=1).

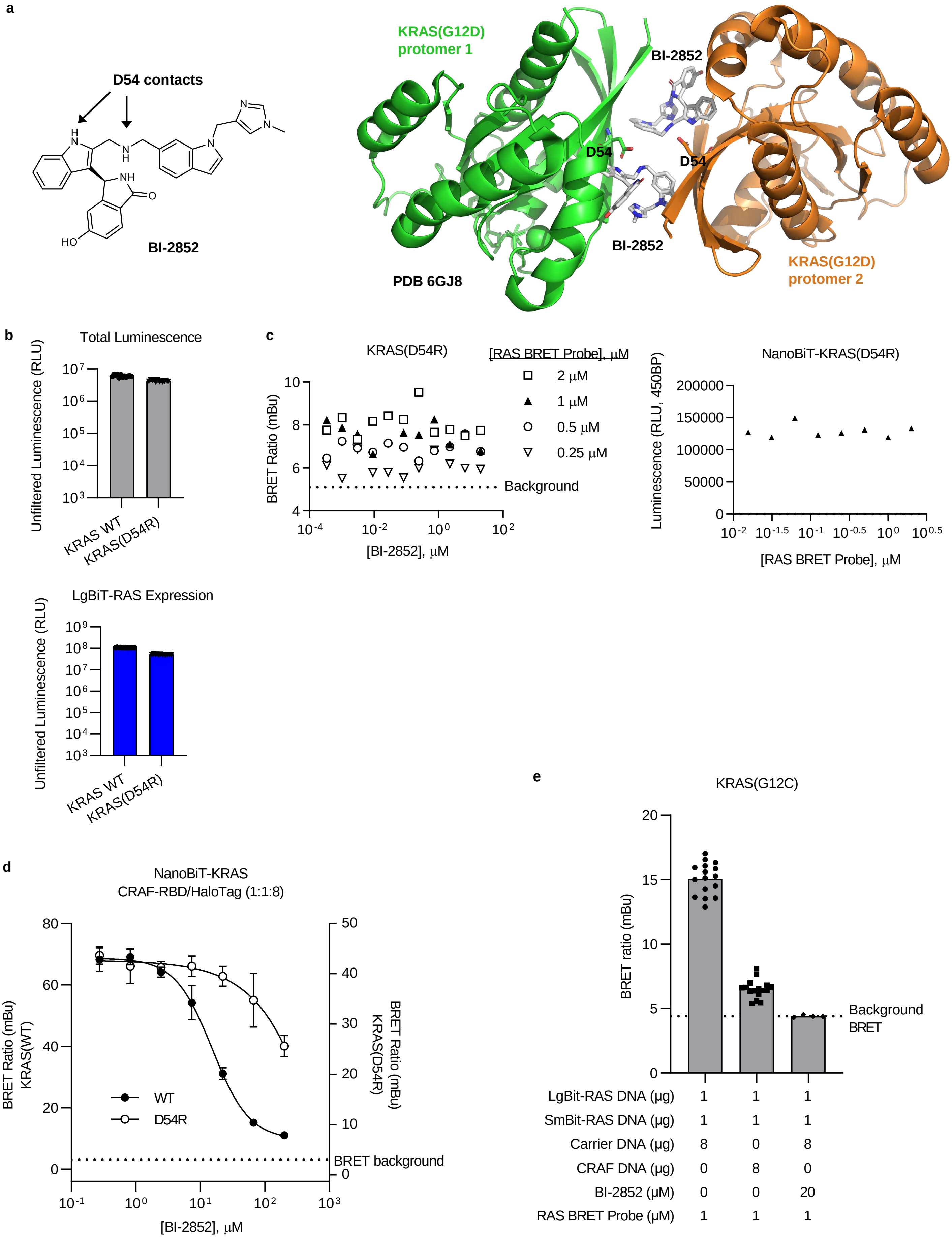
a
D54 contacts
BI-2852
HO
KRAS(G12D)
protomer 1
BI-2852
D54
D54
BI-2852
PDB 6GJ8
KRAS(G12D)
protomer 2
b
Total Luminescence
Unfiltered Luminescence (RLU)
KRAS WT
KRAS(D54R)
c
KRAS(D54R)
[RAS BRET Probe], µM
2 µM
1 µM
0.5 µM
0.25 µM
BRET Ratio (mBu)
Background
[BI-2852], µM
NanoBiT-KRAS(D54R)
Luminescence (RLU, 450BP)
[RAS BRET Probe], µM
LgBiT-RAS Expression
Unfiltered Luminescence (RLU)
KRAS WT
KRAS(D54R)
d
NanoBiT-KRAS
CRAF-RBD/HaloTag (1:1:8)
BRET Ratio (mBu) KRAS(WT)
BRET Ratio (mBu) KRAS(D54R)
WT
D54R
BRET background
[BI-2852], µM
e
KRAS(G12C)
BRET ratio (mBu)
Background BRET
LgBit-RAS DNA (µg) 1 1 1
SmBit-RAS DNA (µg) 1 1 1
Carrier DNA (µg) 8 0 8
CRAF DNA (µg) 0 8 0
BI-2852 (µM) 0 0 20
RAS BRET Probe (µM) 1 1 1

Supplementary Figure 8. The RAS BRET Probe binds with a mechanism consistent with that of BI-2852. **a.** Binding of BI-2852 in the SI/II pocket of KRAS is mediated in part by residue D54 as depicted from the structure of KRAS(G12D) in complex with BI-2852 (PDB 6GJ8, Tran et al.[33]). Mutation of D54 to R should disrupt binding of both BI-2852 and the RAS BRET probe. **b.** Negative control mutant KRAS(D54R) produces similar luminescence (left) and expression (right) compared to KRAS WT. Results are mean $\pm$ S.D. of 12 technical replicates (n=1). **c.** KRAS(D54R) does not produce BRET when titrated with the RAS BRET probe (left) and the RAS BRET probe does not affect the luminescence (right). Results are technical singlicates (n=1). **d.** The NanoBiT/NanoBRET protein-protein interaction assay confirms that NanoBiT-KRAS(D54R) dimers are competent to interact with CRAF-RBD, but with significantly attenuated displacement of the interaction by BI-2852. Results are mean $\pm$ S.D. of 4 technical replicates (n=1). **e.** Co-expression of full length CRAF attenuates the BRET between KRAS(G12C) and the RAS BRET probe, supporting that the RAS BRET probe is competitive with effector interactions. Results are the mean $\pm$ S.D. of 4 or 16 technical replicates as indicated (n=1).

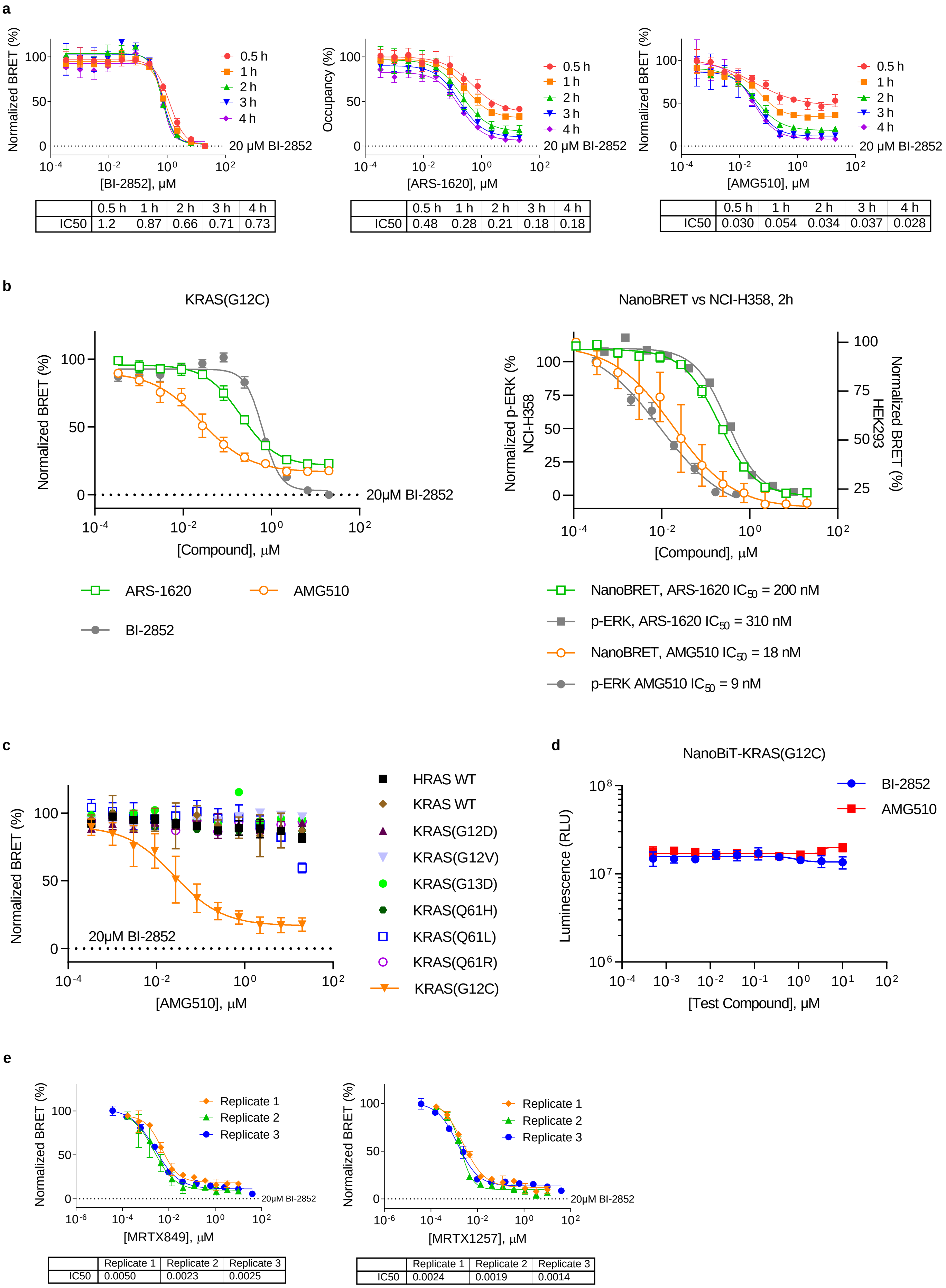

	0.5 h	1 h	2 h	3 h	4 h
IC50	1.2	0.87	0.66	0.71	0.73

	0.5 h	1 h	2 h	3 h	4 h
IC50	0.48	0.28	0.21	0.18	0.18

	0.5 h	1 h	2 h	3 h	4 h
IC50	0.030	0.054	0.034	0.037	0.028

	Replicate 1	Replicate 2	Replicate 3
IC50	0.0050	0.0023	0.0025

	Replicate 1	Replicate 2	Replicate 3
IC50	0.0024	0.0019	0.0014

Supplementary Figure 9. Characterization of SII-P engagement at KRAS(G12C). a. Protracted KRAS(G12C) engagement is observed for SII-P ligands, while rapid engagement is observed for BI-2852. Individual data points are the mean ± S.D. of 4 technical replicates (n=2). Occupancy is calculated relative to a saturating (20 µM) dose of BI-2852, as marked. **b.** Left: BI-2852 as well as SII-P ligands ARS-1620 and AMG510 engage KRAS(G12C) in live cells. BRET is normalized relative to a saturating (20 µM) dose of BI-2852, as marked. Right: Comparison of target engagement versus phospho-ERK as reported by Canon et al.[5] Data are mean of 4 independent experiments ± S.E.M. (n=4). **c.** Live cell BRET target engagement analysis with AMG510 confirming selectivity for KRAS(G12C) versus other RAS variants. BRET is normalized relative to a saturating (20 µM) dose of BI-2852, as marked. Results are the mean ± S.E. of 1−4 independent experiments (n=4 for KRAS WT and KRAS(G12C); n=3 for KRAS(G12D), KRAS(Q61L), and HRAS WT; n=1 for remaining KRAS hotspot mutants). **d.** Like SI/II-P ligand BI-2852, SII-P ligand AMG510 did not affect the luminescence produced by expression of NanoBiT-KRAS(G12C). Results are the mean ± S.D. of 3 technical replicates (n=1). **e.** Target engagement is observed with MRTX849 and MRTX1257 at KRAS(G12C) in live cells. BRET is normalized relative to a saturating (20 µM) dose of BI-2852, as marked. Individual data points are mean ± S.D of 4 technical replicates (n=3).

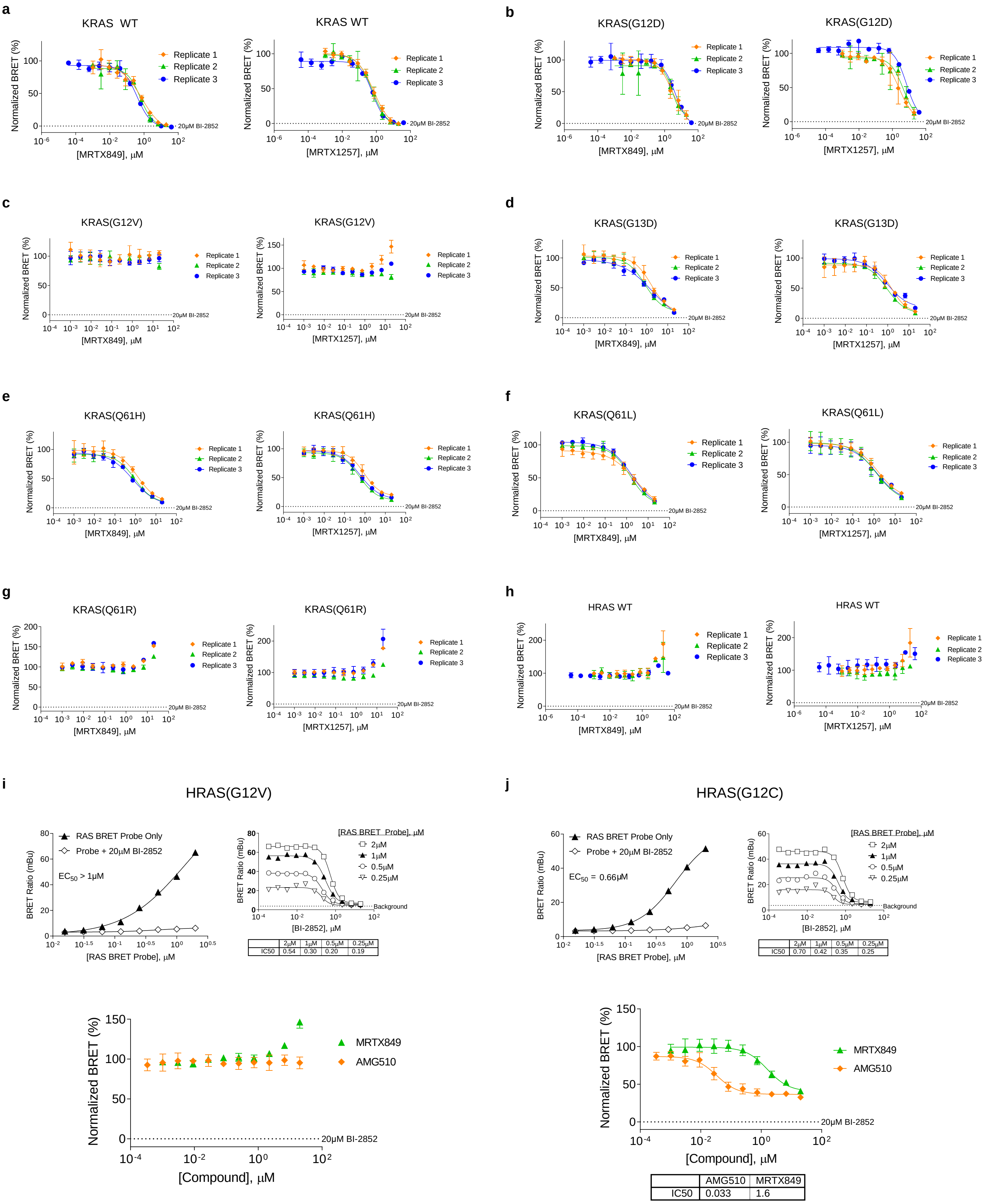
a
KRAS WT
Normalized BRET (%)
[MRTX849], μM
[MRTX1257], μM
Replicate 1
Replicate 2
Replicate 3
20μM BI-2852
b
KRAS(G12D)
c
KRAS(G12V)
d
KRAS(G13D)
e
KRAS(Q61H)
f
KRAS(Q61L)
g
KRAS(Q61R)
h
HRAS WT
i
HRAS(G12V)
RAS BRET Probe Only
Probe + 20μM BI-2852
EC50 > 1μM
BRET Ratio (mBu)
[RAS BRET Probe], μM
[RAS BRET Probe], μM
2μM
1μM
0.5μM
0.25μM
Background
[BI-2852], μM
2μM 1μM 0.5μM 0.25μM
IC50 0.54 0.30 0.20 0.19
j
HRAS(G12C)
EC50 = 0.66μM
IC50 0.70 0.42 0.35 0.25
MRTX849
AMG510
[Compound], μM
AMG510 MRTX849
IC50 0.033 1.6

Supplementary Figure 10. Observation of engagement of KRAS and key hotspot mutants with MRTX849 and MRTX1257. a−h. Reproducibility of MRTX849 and MRTX1257 engagement across RAS isoforms. Data are mean ± S.D. of 4 technical replicates (n=3). BRET is normalized relative to a saturating (20 µM) dose of BI-2852, as marked. **i, j.** Characterization of BRET assay (n=1) and SII-P engagement for HRAS(G12V) and HRAS(G12C). Data for RAS BRET probe binding and BI-2852 engagement are technical singlicates (n=1), and data for MRTX849 and AMG510 engagement are the mean ± S.D. of 4 technical replicates (n=1). Instrument background BRET values are indicated by the dashed line marked "background". For competition with AMG510 and MRTX849, BRET is normalized relative to a saturating (20 µM) dose of BI-2852, as marked.

a

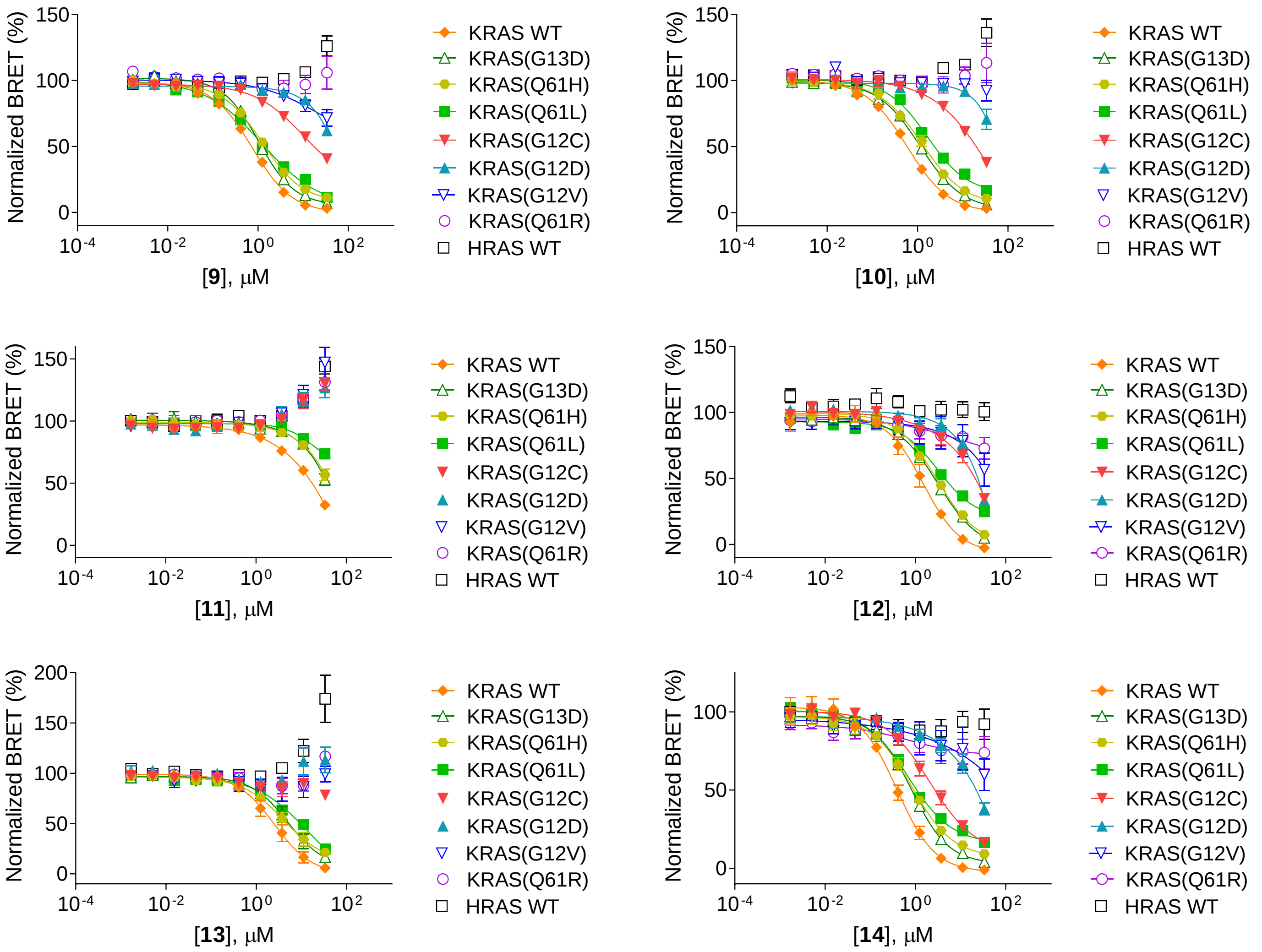

b

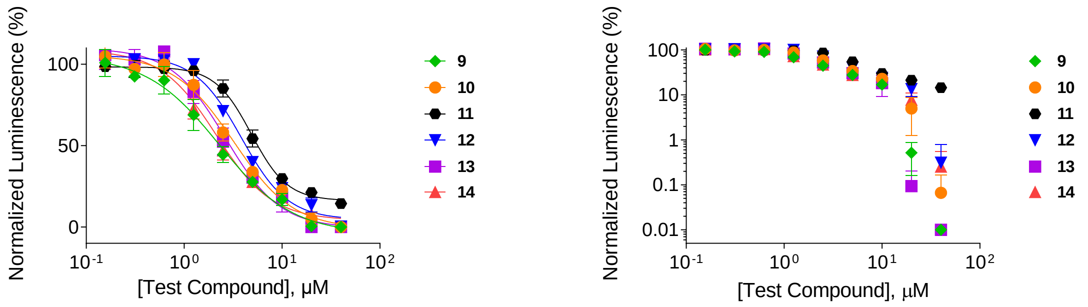

	9	10	11	12	13	14
IC50	2.3	3.2	4.9	3.8	2.7	2.1

	9	10	11	12	13	14
IC50	2.3	3.2	4.9	3.8	2.7	2.1

c

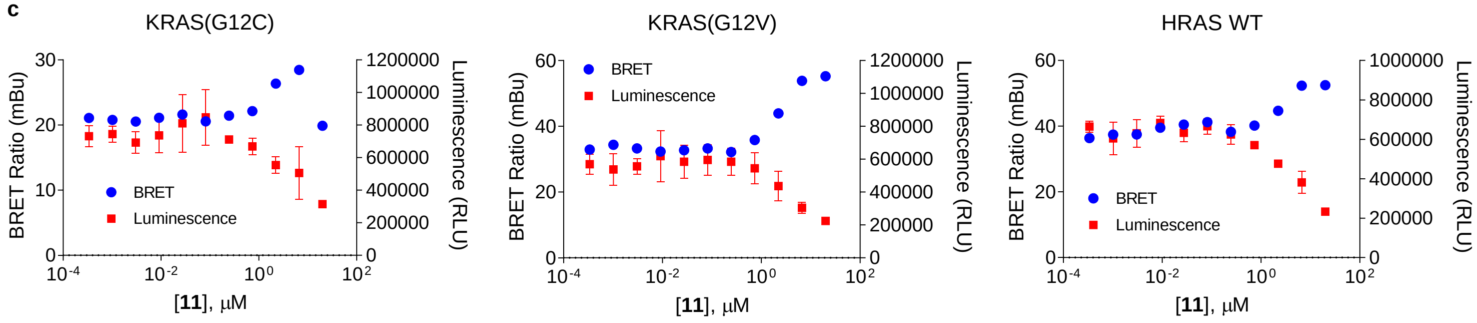

Supplementary Figure 11. Characterization of RAS engagement for reversible saturated amides of MRTX849. a. BRET target engagement profiles of **9-14** for KRAS and HRAS, as well as KRAS hotspot mutants. Data are mean of 3 independent experiments, each performed with 4 technical replicates ± S.E.M. (n=3). **b.** Antiproliferative/cytotoxic effect of **9-14** in RAS-independent HEK293 cells using CellTiter-Glo. Data are depicted on a linear (left) and a log (right) scale to better visualize the biphasic activity of **9-14**. Data are mean of technical triplicates (n=1). **c.** Spurious increases in BRET as exemplified by **11** for KRAS(G12C), KRAS(G12V), and HRAS WT are associated with a decrease in donor signal and likely associated with the observed cytotoxicity. Individual data points are the mean ± S.D. of 3-4 technical replicates (n=1).

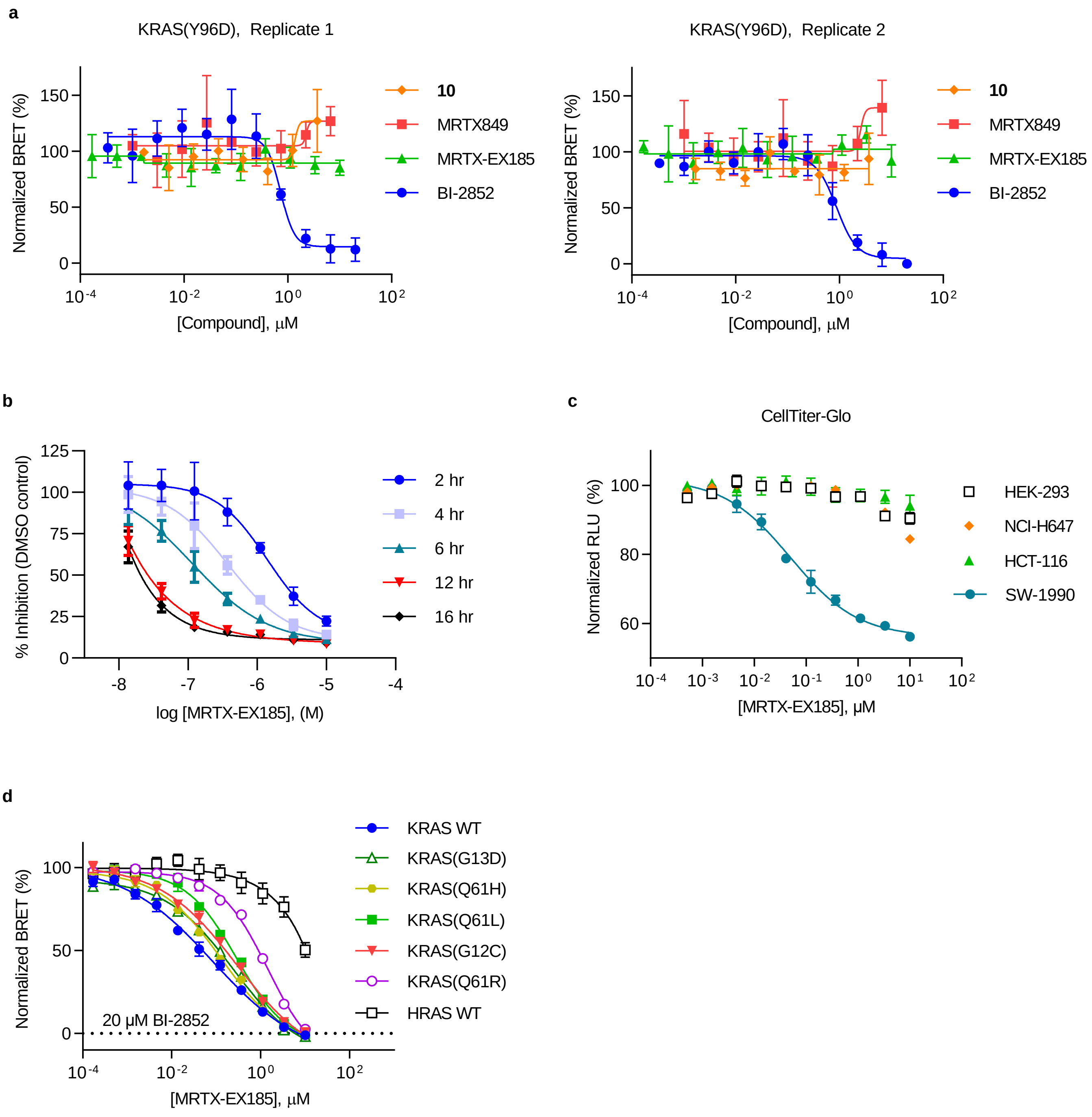

Supplementary Figure 12. Characterization of MRTX-EX185. a. Characterization of engagement of compounds to KRAS(Y96D). Individual data are mean of 4 technical replicates ± S.D. (n=2). **b.** Inhibition of KRAS(G12D) : CRAF(RBD) in live cells using NanoBiT. Individual data points are the mean ± S.D. of 6 technical replicates (n=1). **c.** Anti-proliferative effects of MRTX-EX185 in various cell lines. Data are mean of 3 independent experiments, each performed with at least 3 technical replicates ± S.E.M. (n=3). **d.** Engagement of MRTX-EX185 to KRAS hotspot mutants. For KRAS WT, individual data points are the mean ± S.E. of 4 independent experiments (n=4). For all other RAS variants, individual data points are the mean of 4 technical replicates ± S.D. (n=1). BRET is normalized relative to a saturating (20 µM) dose of BI-2852, as marked.

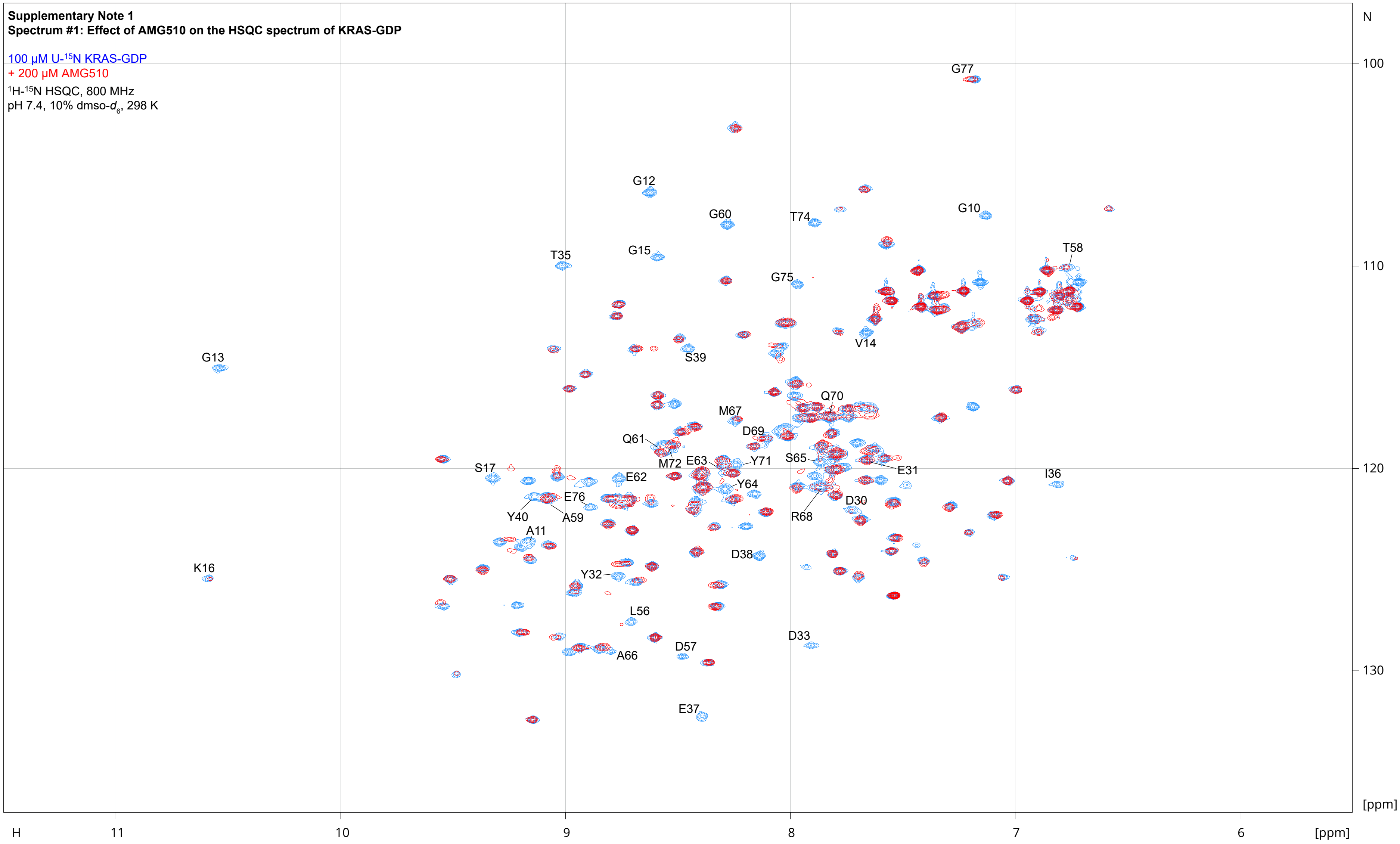
Supplementary Note 1
Spectrum #1: Effect of AMG510 on the HSQC spectrum of KRAS-GDP
100 μM U-15N KRAS-GDP
+ 200 μM AMG510
1H-15N HSQC, 800 MHz
pH 7.4, 10% dmso-d6, 298 K
G77
G12
G60
T74
G10
T35
G15
T58
G75
G13
S39
V14
Q70
M67
D69
Q61
M72
E63
Y71
S65
E31
S17
E62
I36
Y64
E76
D30
Y40
A59
R68
A11
D38
K16
Y32
L56
D33
D57
A66
E37
N
100
110
120
130
[ppm]
H
11
10
9
8
7
6
[ppm]

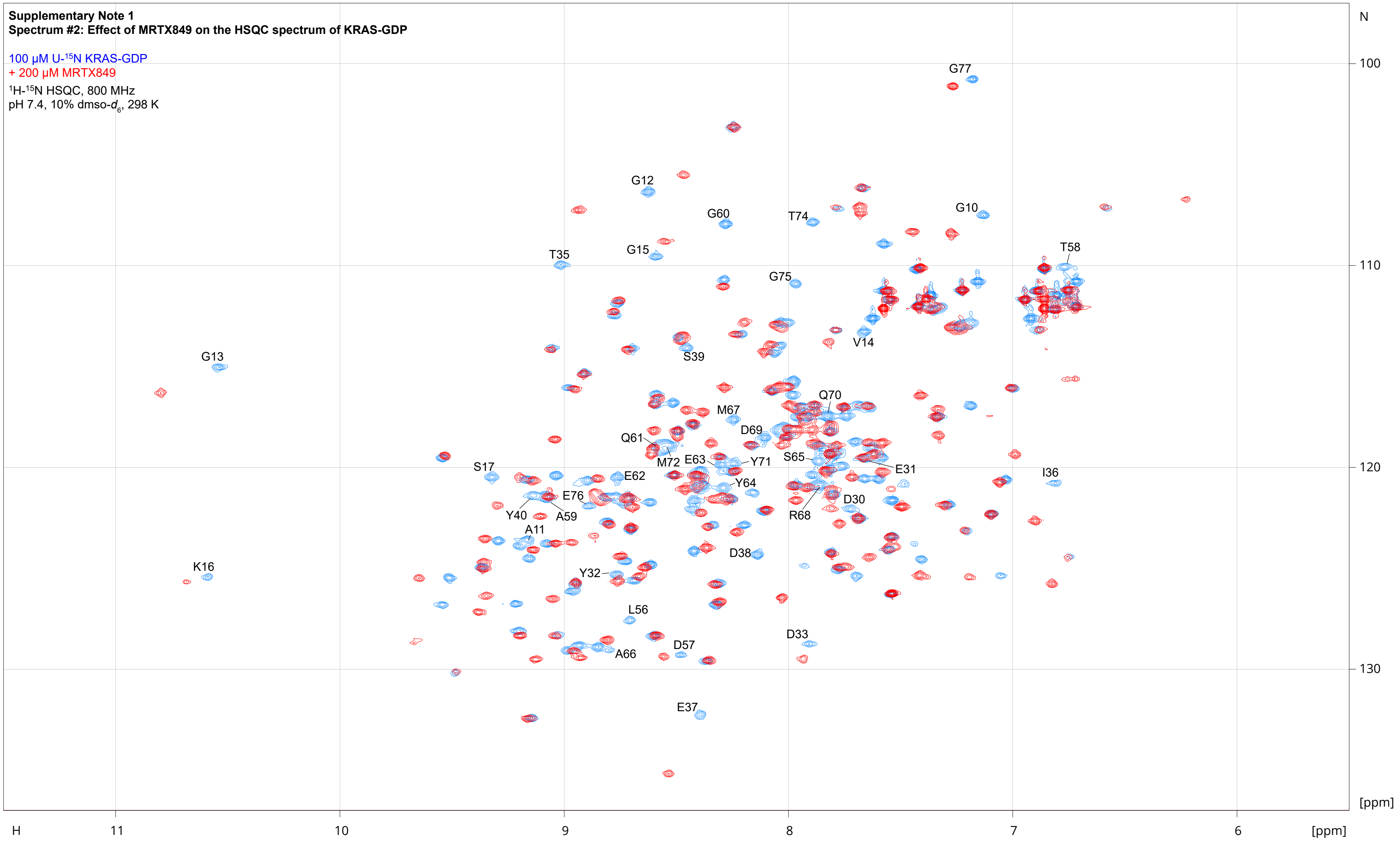
Supplementary Note 1
Spectrum #2: Effect of MRTX849 on the HSQC spectrum of KRAS-GDP
100 μM U-15N KRAS-GDP
+ 200 μM MRTX849
1H-15N HSQC, 800 MHz
pH 7.4, 10% dmso-d6, 298 K
G77
G12
G60
T74
G10
T35
G15
G75
T58
V14
G13
S39
Q70
M67
D69
Q61
M72
E63
Y71
S65
E31
S17
E62
Y64
I36
E76
D30
Y40
A59
R68
A11
D38
K16
Y32
L56
D33
D57
A66
E37
N
100
110
120
130
[ppm]
H
11
10
9
8
7
6
[ppm]

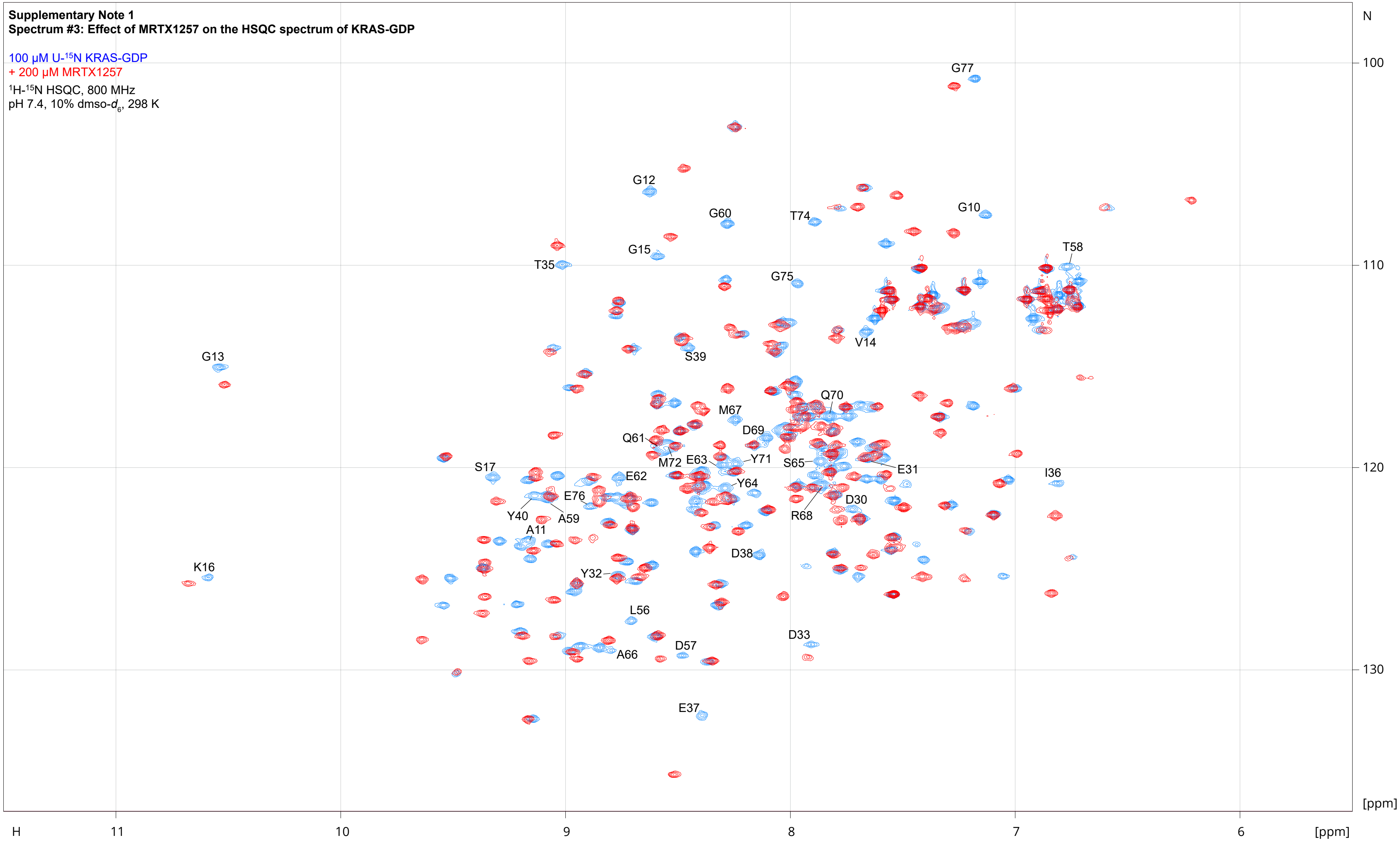

Supplementary Note 1
Spectrum #3: Effect of MRTX1257 on the HSQC spectrum of KRAS-GDP
100 µM U-15N KRAS-GDP
+ 200 µM MRTX1257
1H-15N HSQC, 800 MHz
pH 7.4, 10% dmso-d6, 298 K
N
100
110
120
130
[ppm]
H
11
10
9
8
7
6
[ppm]
G77
G12
G60
T74
G10
G15
T58
T35
G75
V14
G13
S39
Q70
M67
D69
Q61
M72
E63
Y71
S65
E31
S17
E62
I36
Y64
E76
D30
Y40
A59
R68
A11
D38
K16
Y32
L56
D33
D57
A66
E37

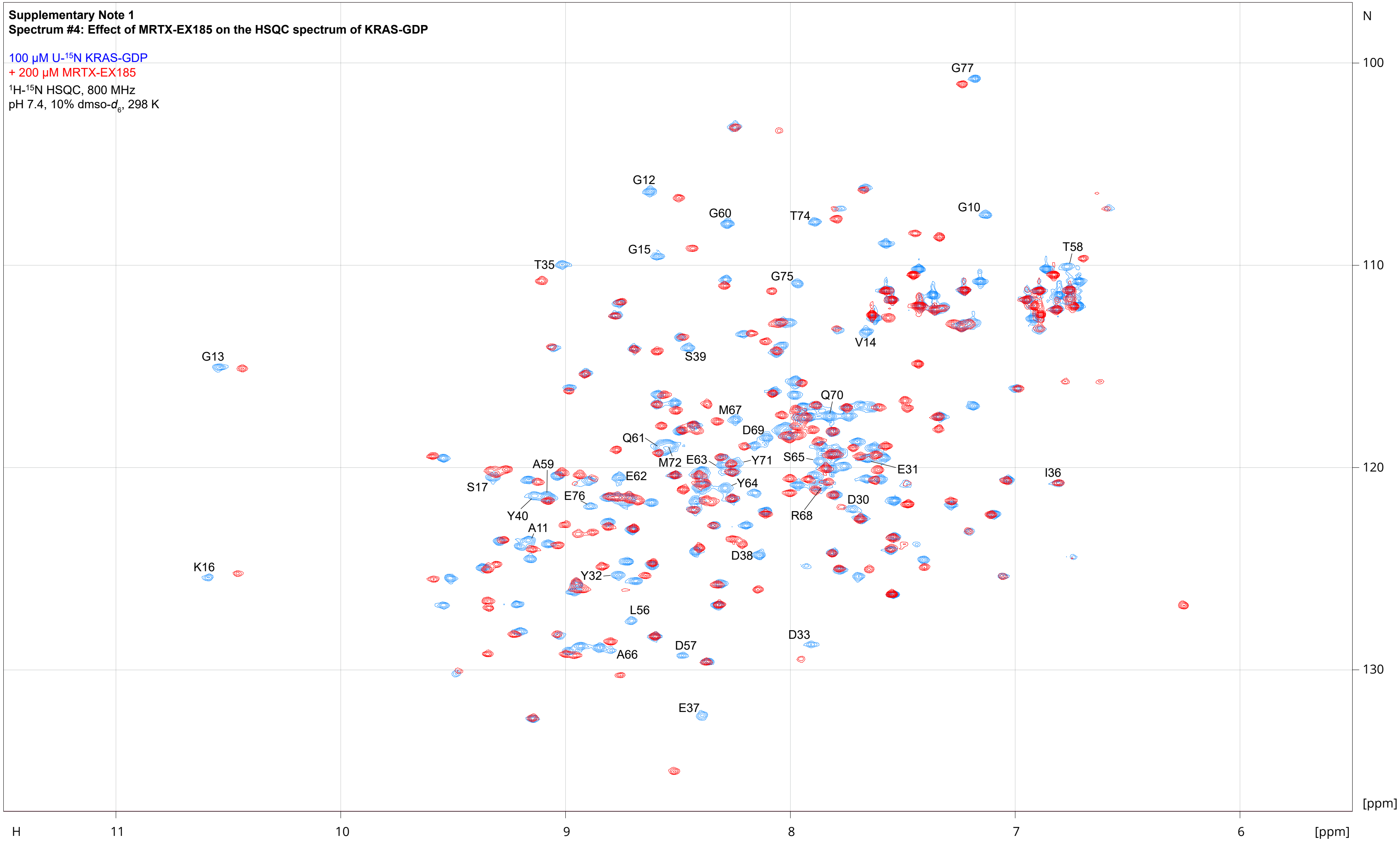
Supplementary Note 1
Spectrum #4: Effect of MRTX-EX185 on the HSQC spectrum of KRAS-GDP
100 μM U-15N KRAS-GDP
+ 200 μM MRTX-EX185
1H-15N HSQC, 800 MHz
pH 7.4, 10% dmso-d6, 298 K
G77
G12
G60
T74
G10
G15
T58
T35
G75
V14
G13
S39
Q70
M67
D69
Q61
M72
E63
Y71
S65
E31
A59
E62
I36
S17
Y64
E76
D30
Y40
R68
A11
D38
K16
Y32
L56
D33
D57
A66
E37
N
100
110
120
130
[ppm]
H
11
10
9
8
7
6
[ppm]

Supplementary Note 1

Spectrum #5: Effect of AMG510 on the HSQC spectrum of KRAS(G12D)-GDP

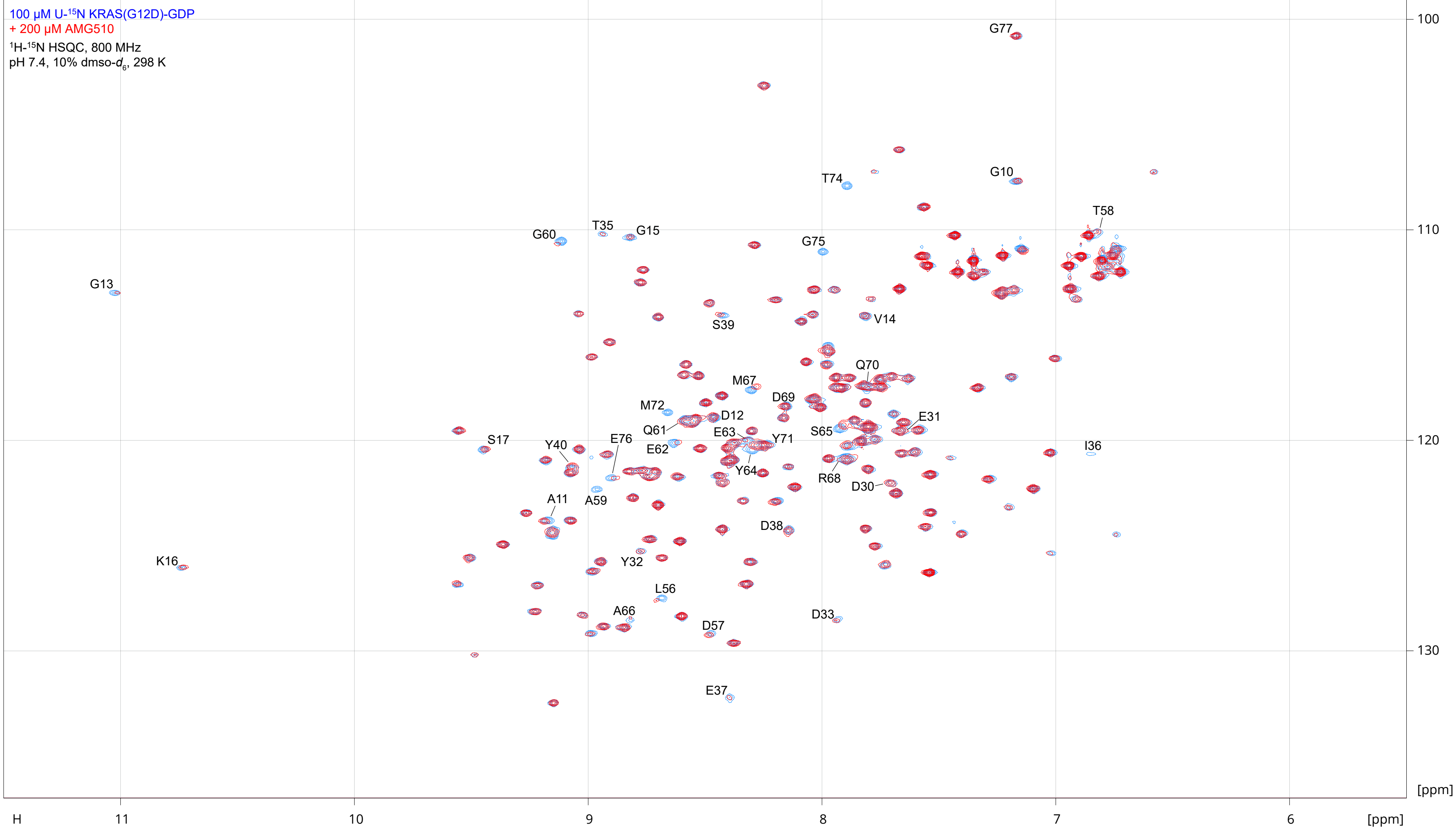

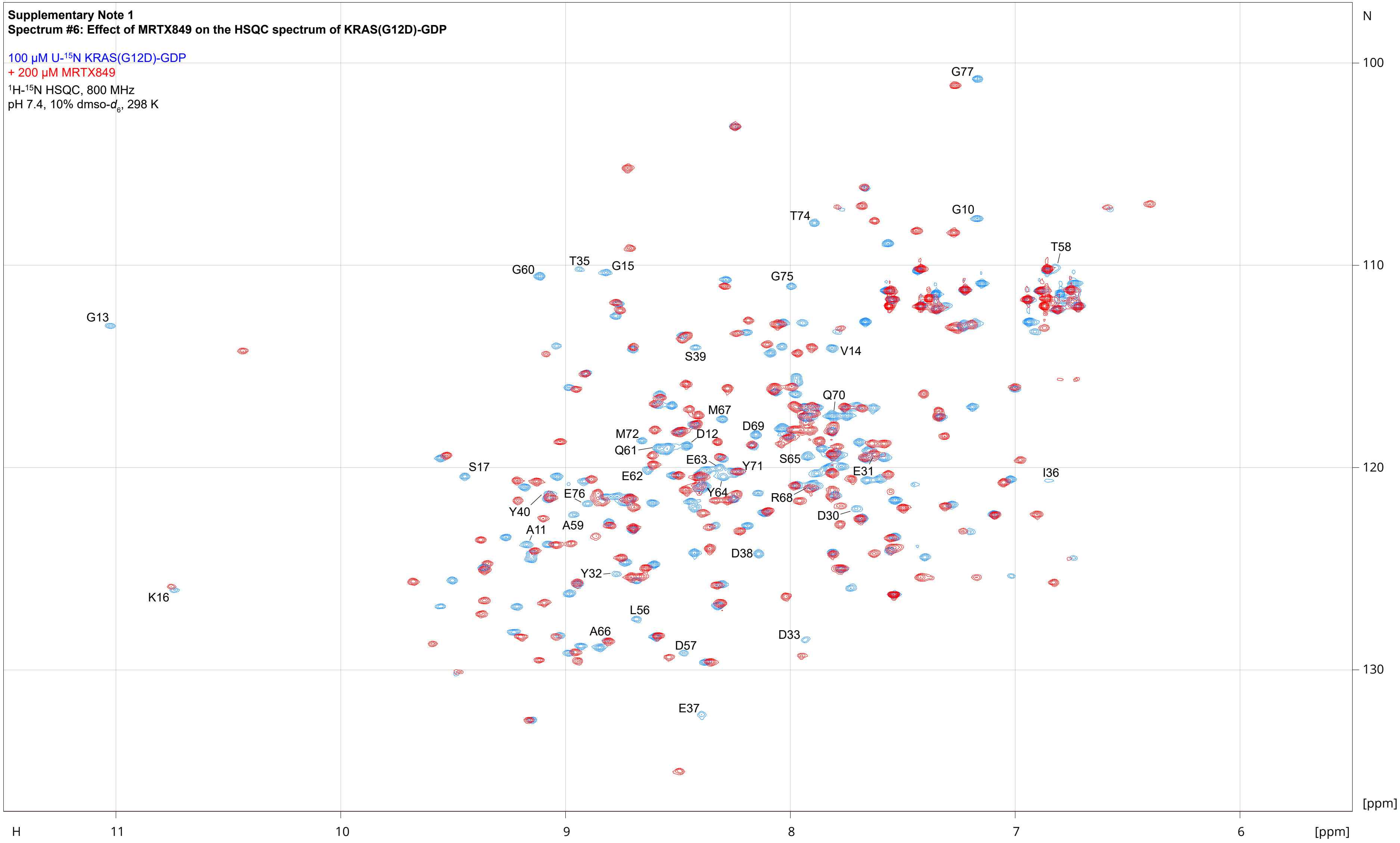
Supplementary Note 1
Spectrum #6: Effect of MRTX849 on the HSQC spectrum of KRAS(G12D)-GDP
100 µM U-^{15}N KRAS(G12D)-GDP
+ 200 µM MRTX849
^{1}H-^{15}N HSQC, 800 MHz
pH 7.4, 10% dmso-d_6, 298 K
G77
G10
T74
T58
T35
G15
G60
G75
G13
S39
V14
Q70
M67
D69
M72
D12
Q61
E63
Y71
S65
S17
E62
E31
I36
E76
Y64
R68
Y40
D30
A59
A11
D38
Y32
K16
L56
A66
D57
D33
E37
N
100
110
120
130
[ppm]
H
11
10
9
8
7
6
[ppm]

Supplementary Note 1

Spectrum #7: Effect of MRTX1257 on the HSQC spectrum of KRAS(G12D)-GDP

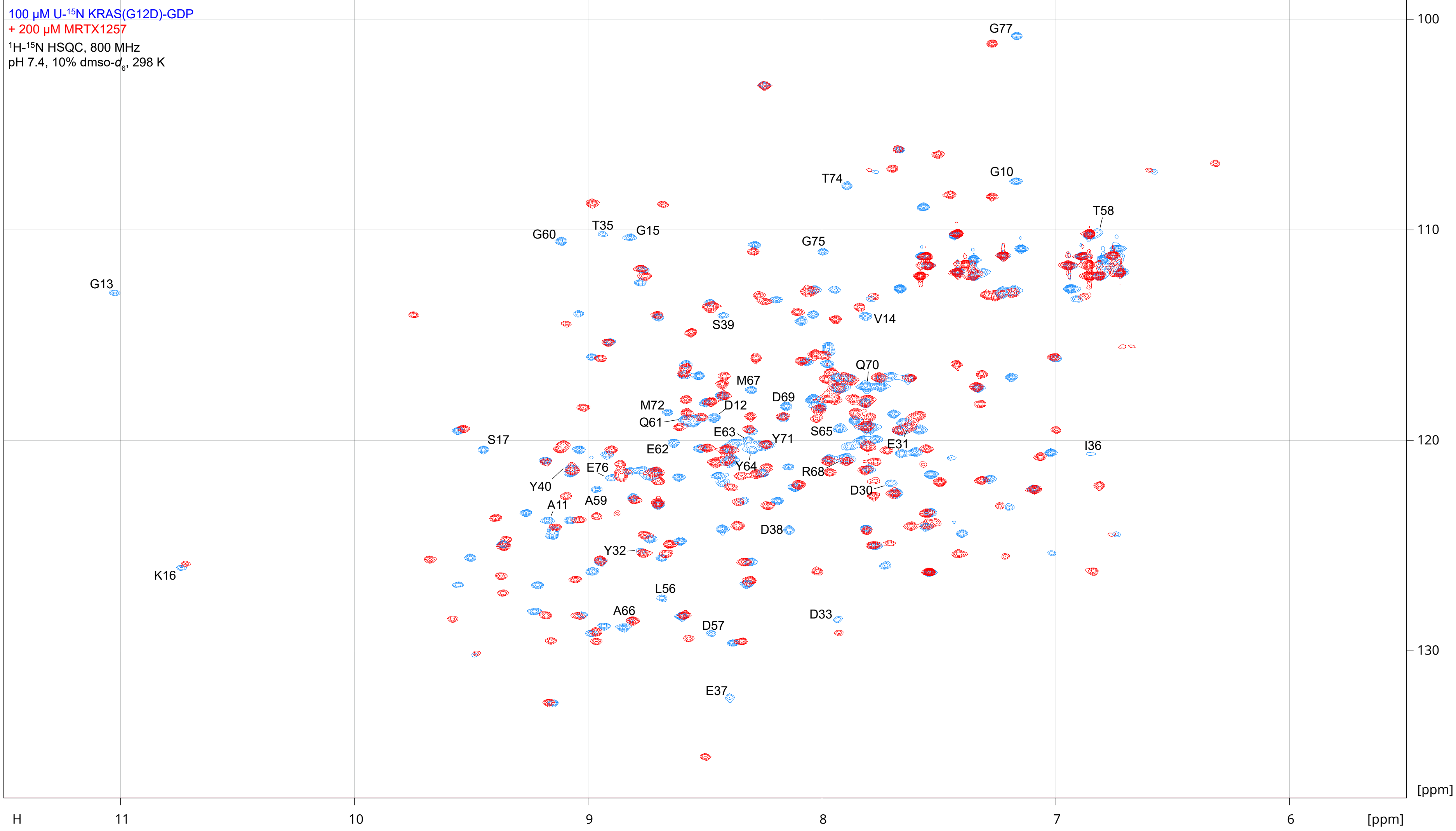

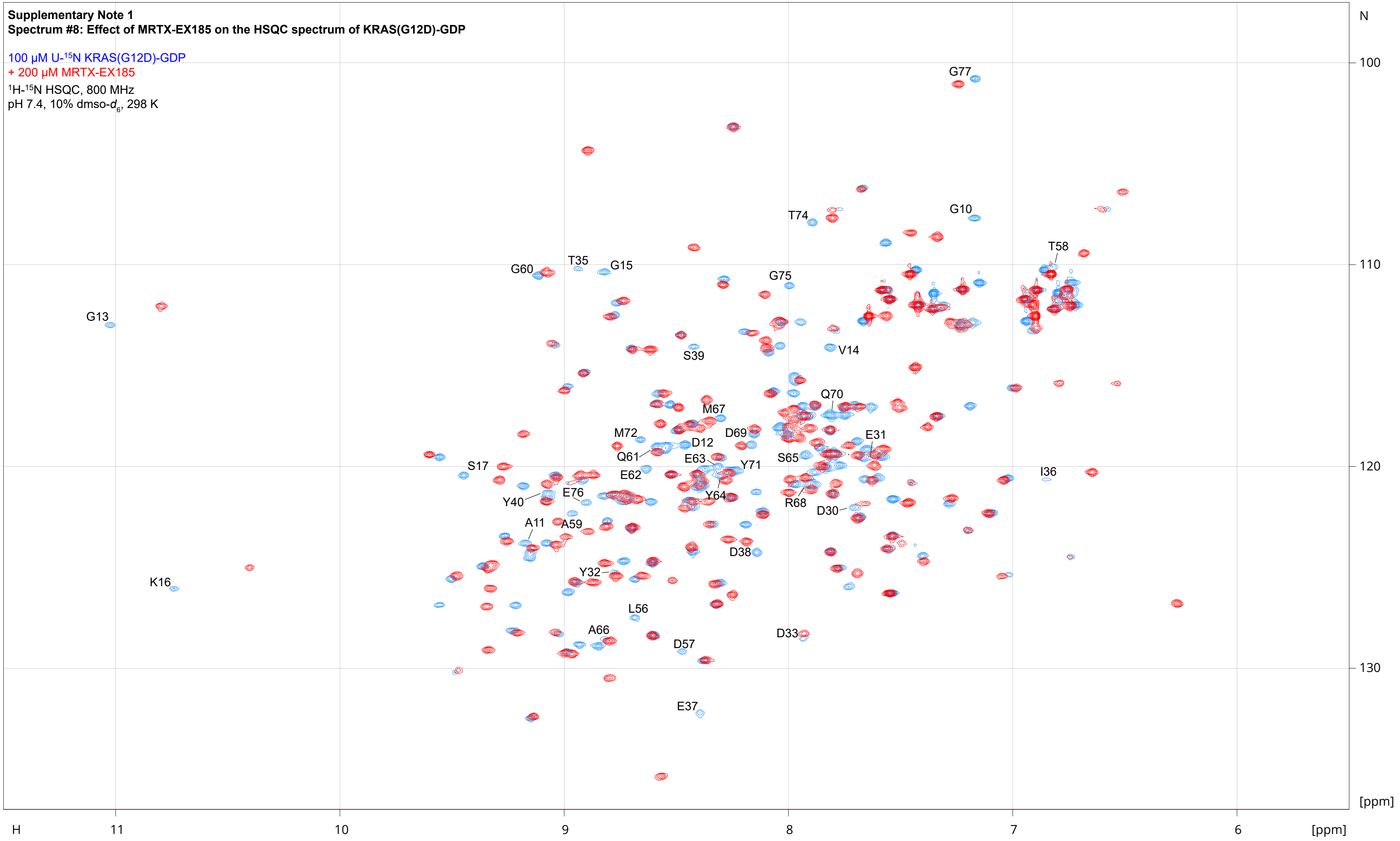

Supplementary Note 1
Spectrum #8: Effect of MRTX-EX185 on the HSQC spectrum of KRAS(G12D)-GDP
100 µM U-15N KRAS(G12D)-GDP
+ 200 µM MRTX-EX185
1H-15N HSQC, 800 MHz
pH 7.4, 10% dmso-d6, 298 K
N
100
110
120
130
[ppm]
H
11
10
9
8
7
6
[ppm]
G77
T74
G10
T58
G60
T35
G15
G75
G13
S39
V14
Q70
M67
M72
D12
D69
E31
Q61
E63
S65
S17
Y71
E62
I36
Y40
E76
Y64
R68
D30
A11
A59
D38
K16
Y32
L56
A66
D57
D33
E37

Supplementary Note 1
Spectrum #9: Effect of AMG510 on the HSQC spectrum of HRAS-GDP

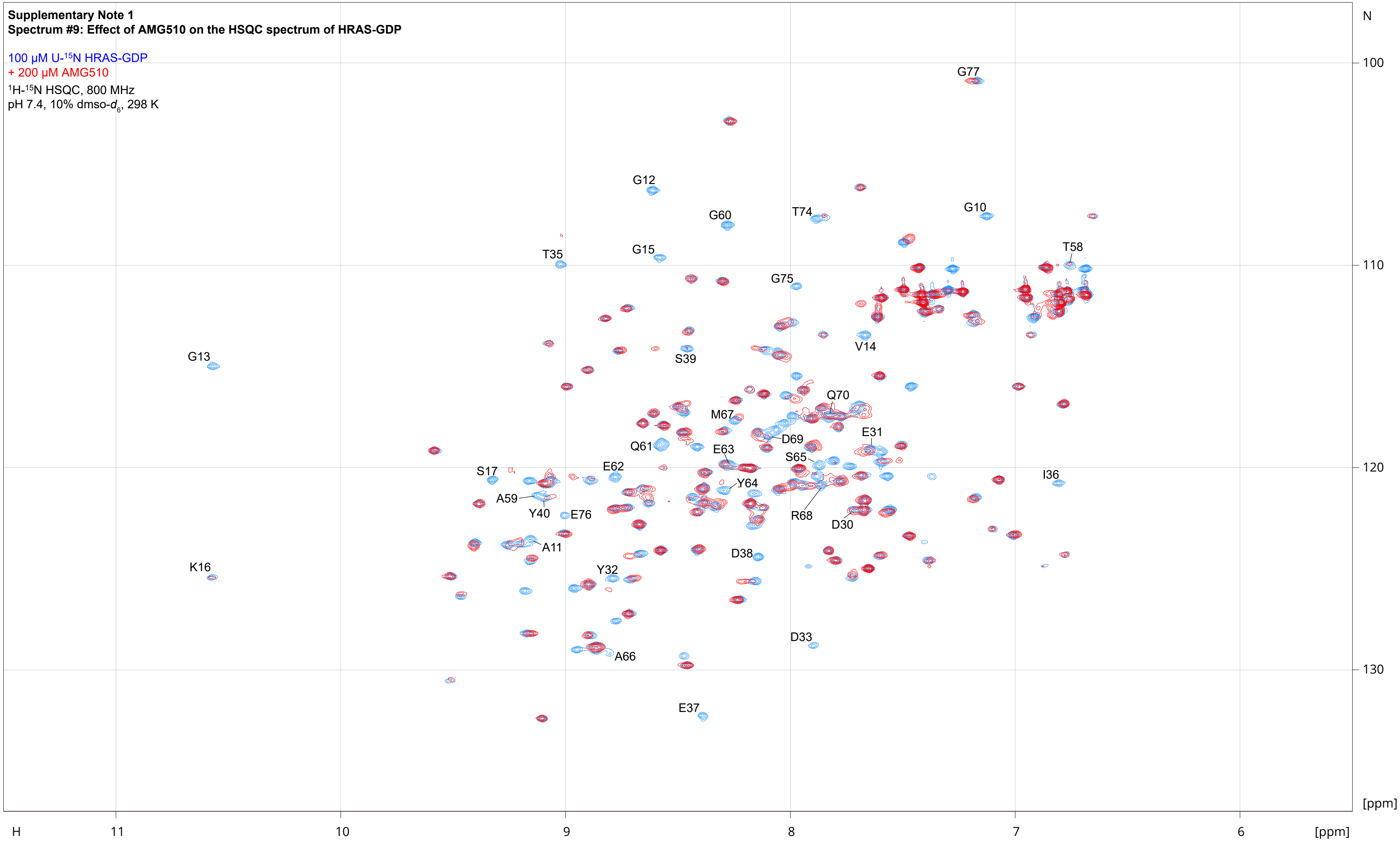

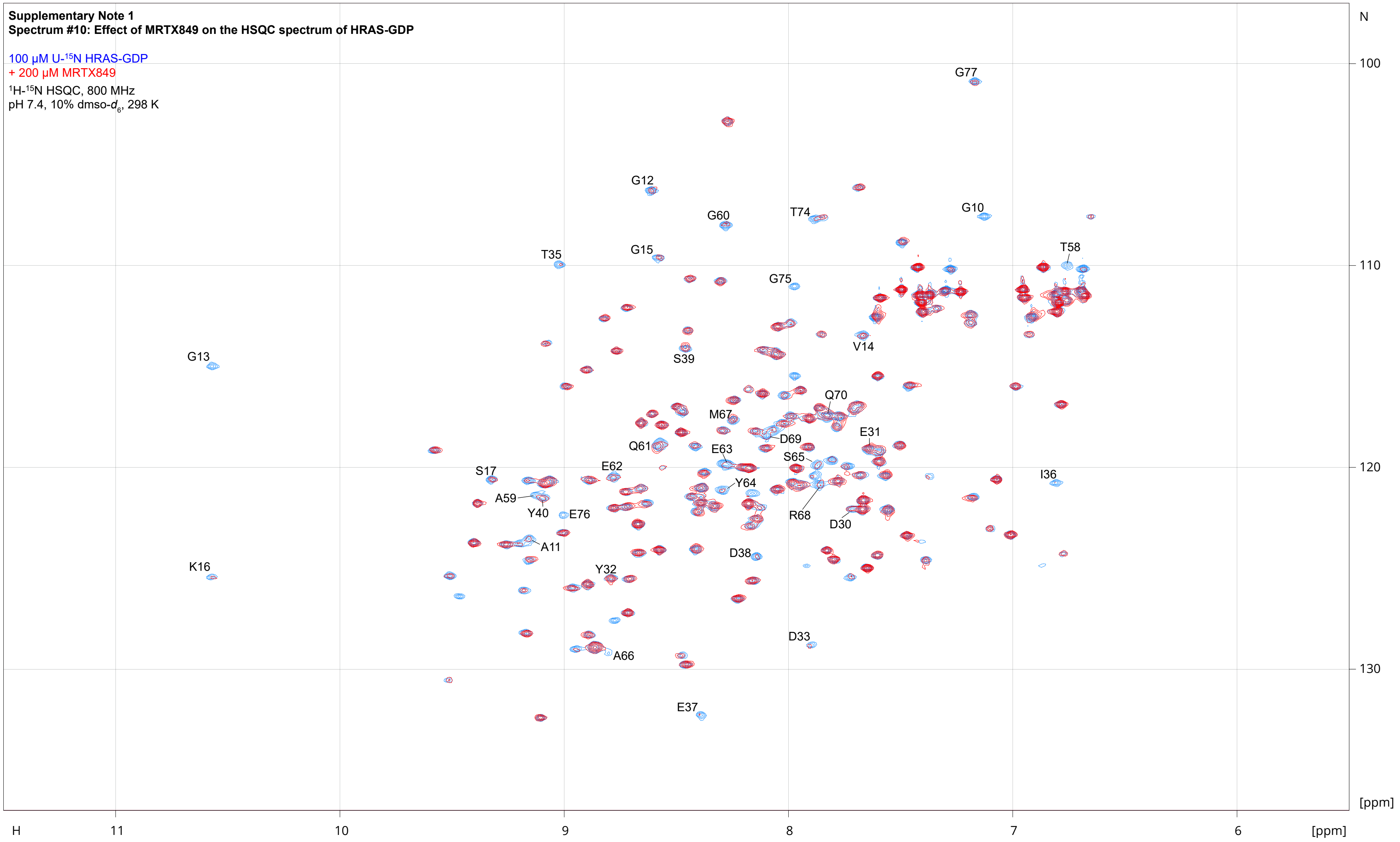
Supplementary Note 1
Spectrum #10: Effect of MRTX849 on the HSQC spectrum of HRAS-GDP
100 μM U-^{15}N HRAS-GDP
+ 200 μM MRTX849
^{1}H-^{15}N HSQC, 800 MHz
pH 7.4, 10% dmso-d_6, 298 K
G77
G12
G60
T74
G10
T35
G15
T58
G75
G13
S39
V14
Q70
M67
D69
E31
Q61
E63
S65
E62
S17
Y64
I36
A59
Y40
E76
R68
D30
A11
D38
K16
Y32
D33
A66
E37
N
100
110
120
130
[ppm]
H
11
10
9
8
7
6
[ppm]

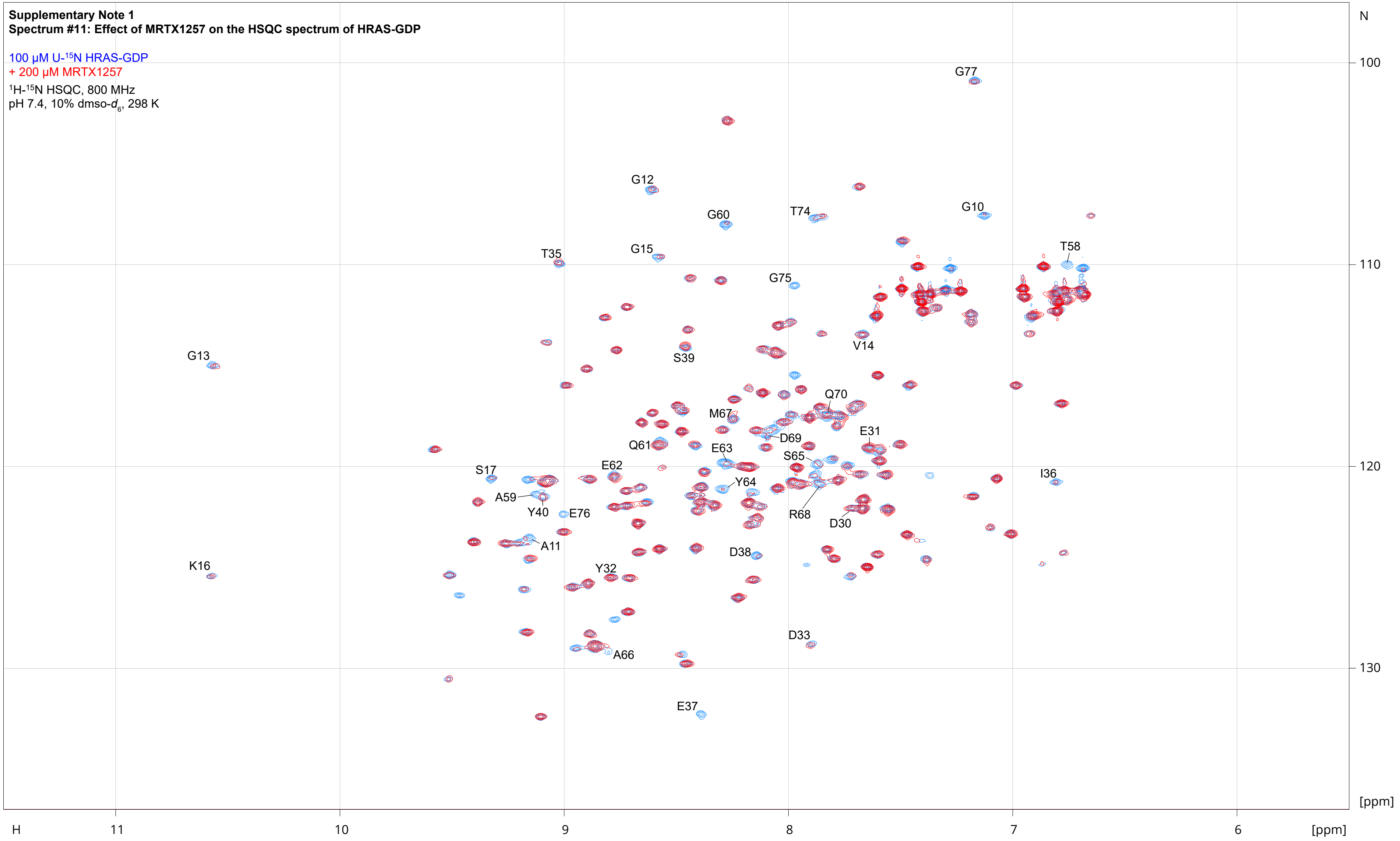
Supplementary Note 1
Spectrum #11: Effect of MRTX1257 on the HSQC spectrum of HRAS-GDP
100 µM U-15N HRAS-GDP
+ 200 µM MRTX1257
1H-15N HSQC, 800 MHz
pH 7.4, 10% dmso-d6, 298 K
N
100
110
120
130
[ppm]
H
11
10
9
8
7
6
[ppm]
G77
G12
G60
T74
G10
T35
G15
T58
G75
G13
S39
V14
Q70
M67
D69
E31
Q61
E63
S65
S17
E62
I36
Y64
A59
Y40
E76
R68
D30
A11
K16
D38
Y32
D33
A66
E37

Supplementary Note 1

Spectrum #12: Effect of MRTX-EX185 on the HSQC spectrum of HRAS-GDP

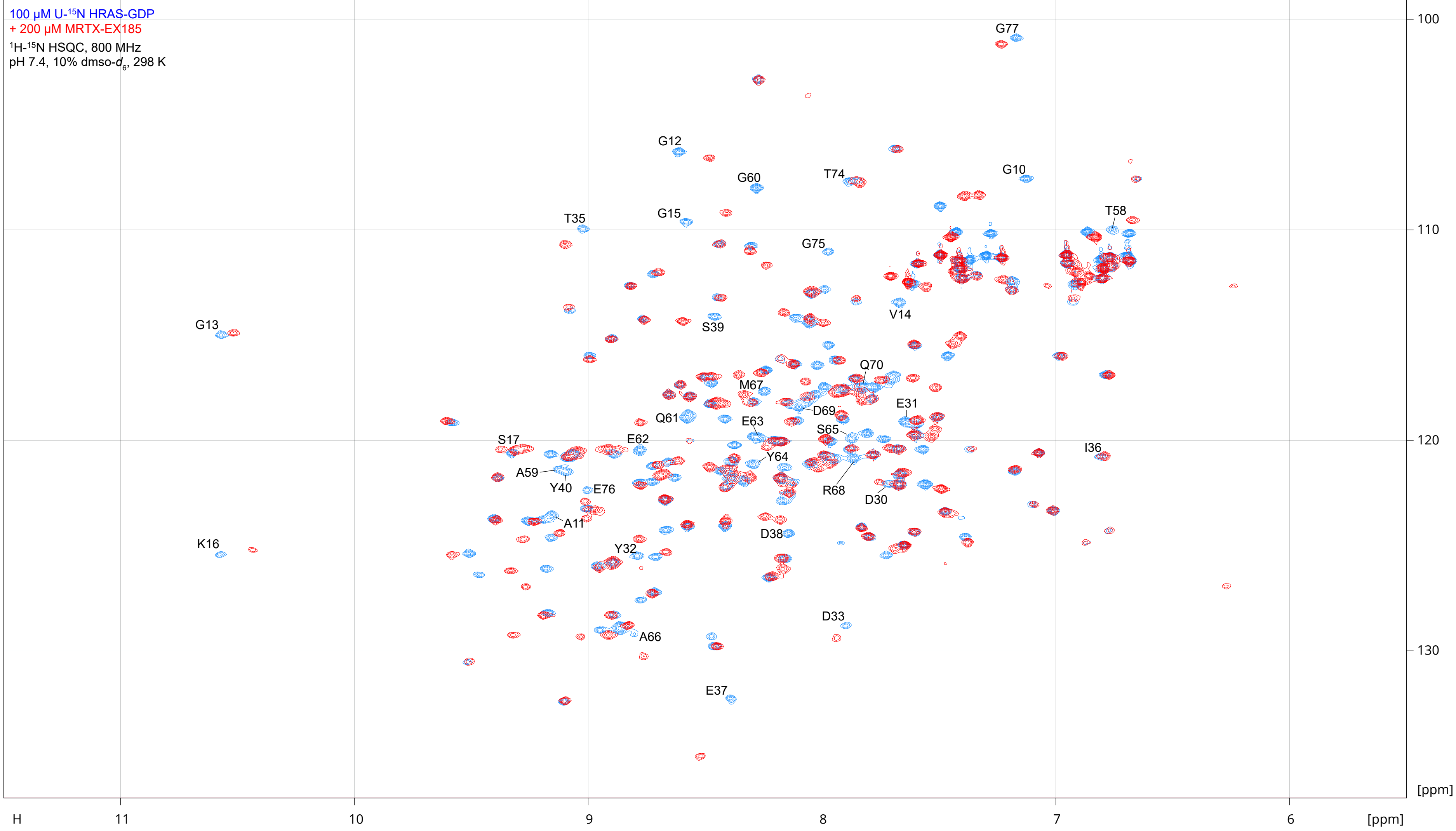

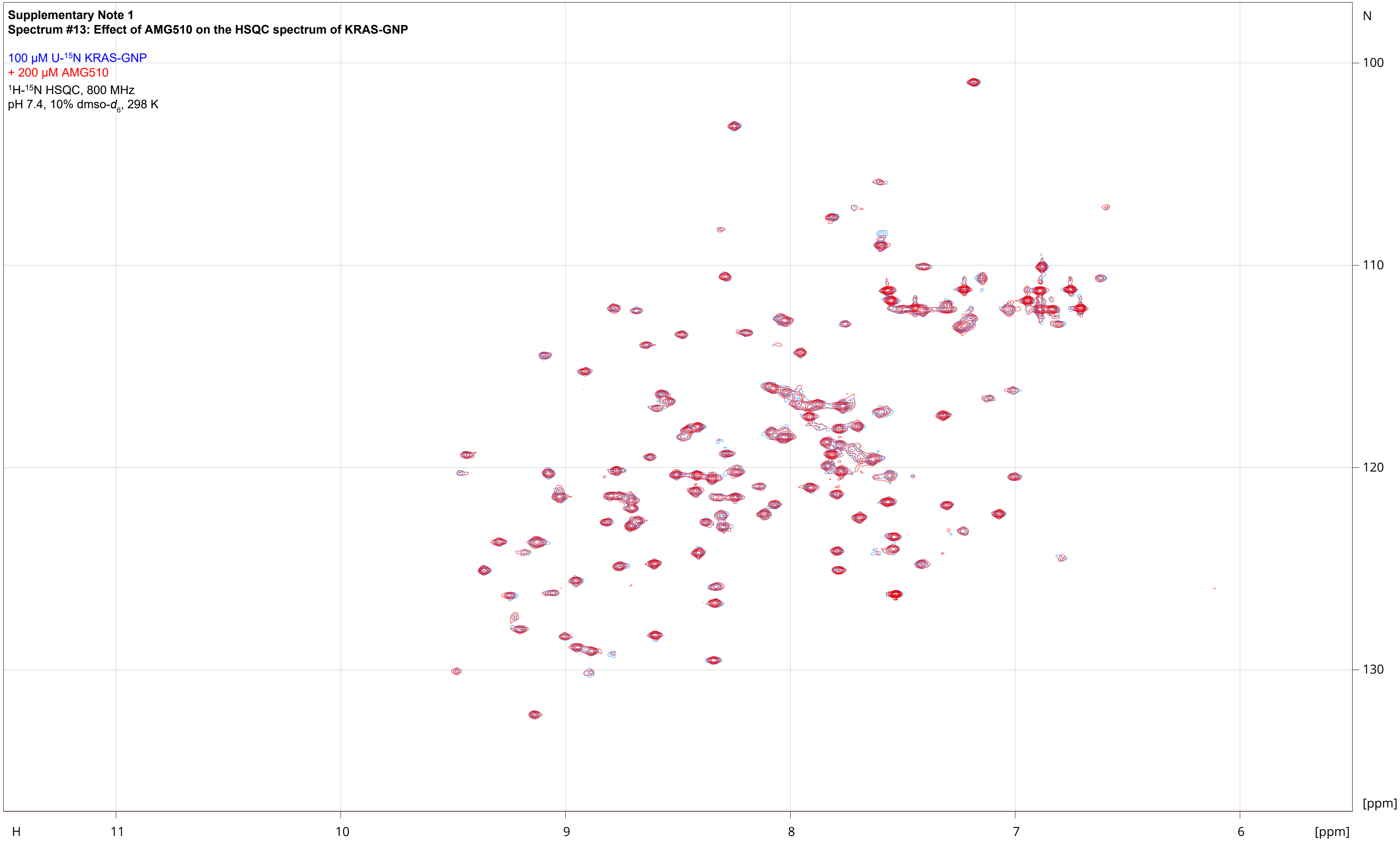
Supplementary Note 1
Spectrum #13: Effect of AMG510 on the HSQC spectrum of KRAS-GNP
100 µM U-15N KRAS-GNP
+ 200 µM AMG510
1H-15N HSQC, 800 MHz
pH 7.4, 10% dmso-d6, 298 K
N
100
110
120
130
[ppm]
H
11
10
9
8
7
6
[ppm]

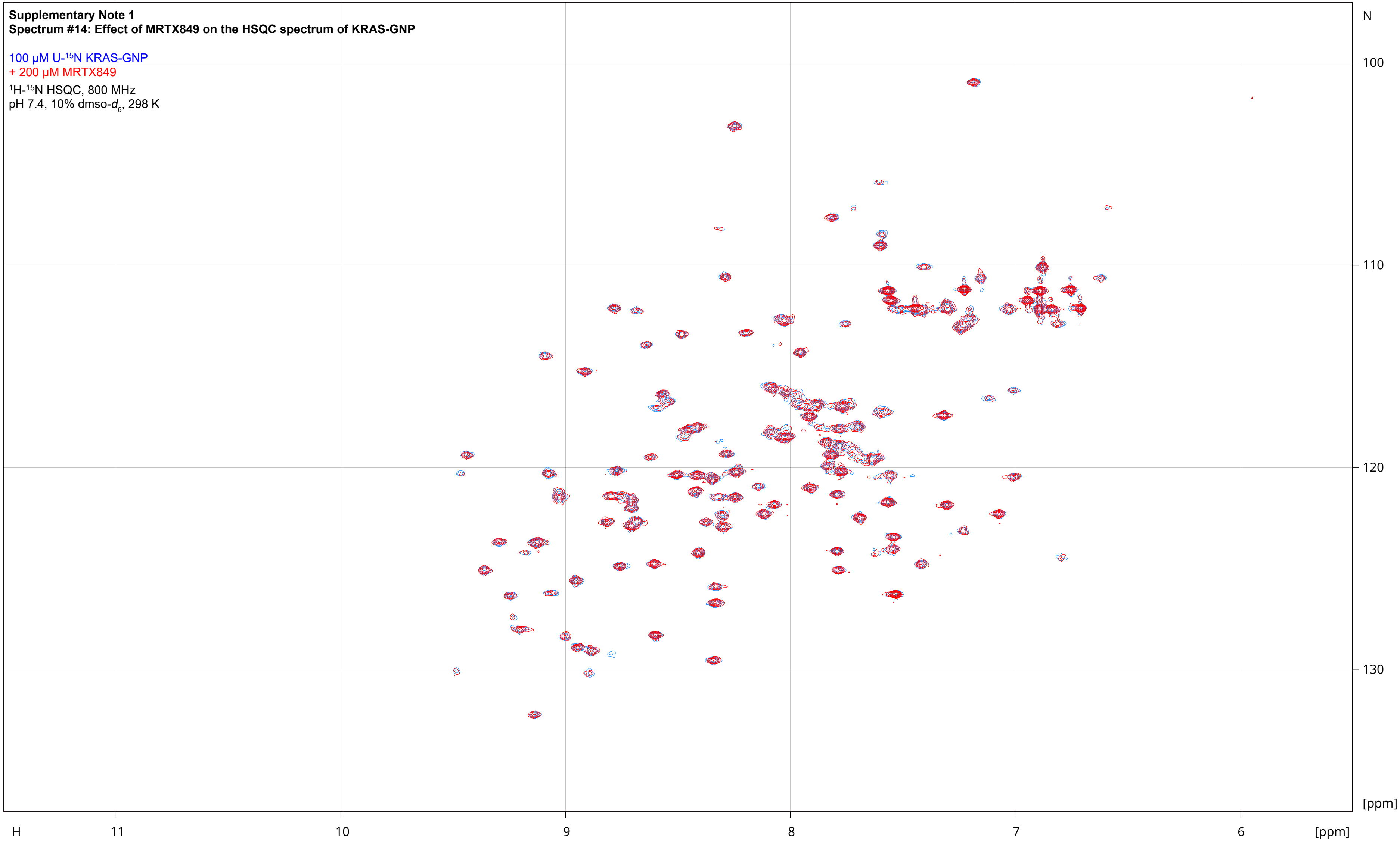
Supplementary Note 1
Spectrum #14: Effect of MRTX849 on the HSQC spectrum of KRAS-GNP
100 μM U-15N KRAS-GNP
+ 200 μM MRTX849
1H-15N HSQC, 800 MHz
pH 7.4, 10% dmso-d6, 298 K
N
100
110
120
130
[ppm]
H
11
10
9
8
7
6
[ppm]

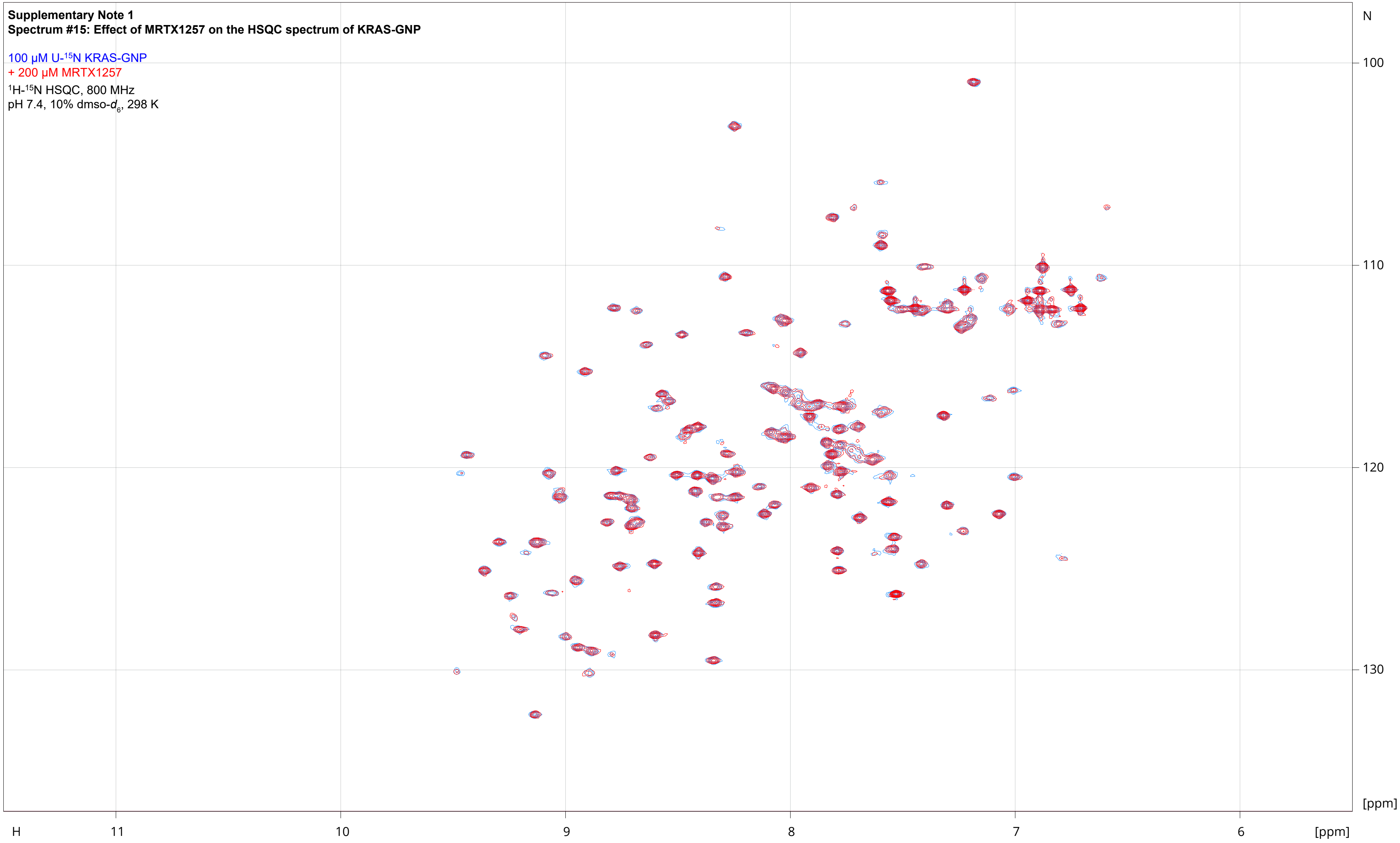
Supplementary Note 1
Spectrum #15: Effect of MRTX1257 on the HSQC spectrum of KRAS-GNP
100 μM U-15N KRAS-GNP
+ 200 μM MRTX1257
1H-15N HSQC, 800 MHz
pH 7.4, 10% dmso-d6, 298 K
N
100
110
120
130
[ppm]
H
11
10
9
8
7
6
[ppm]

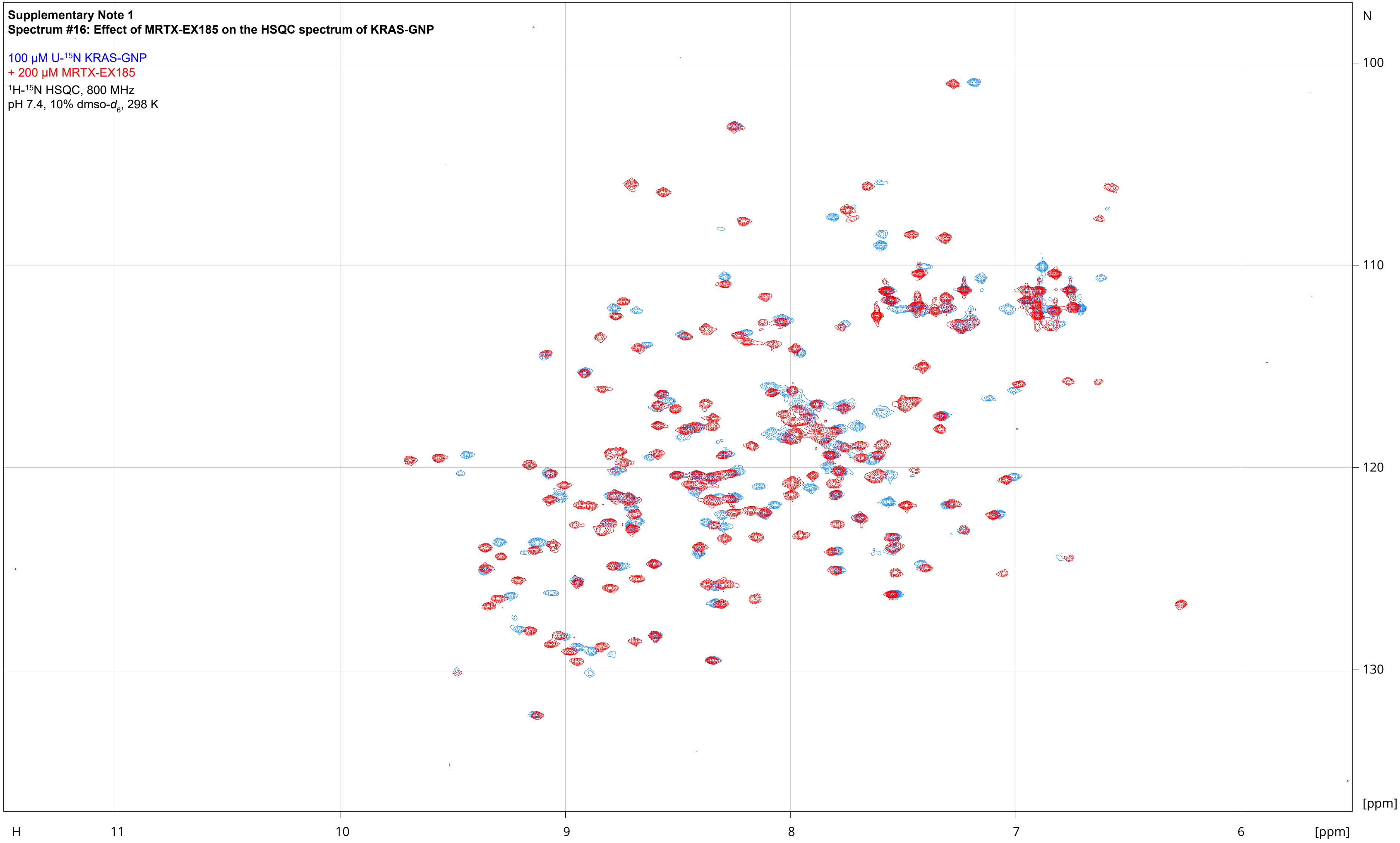

Supplementary Note 1
Spectrum #16: Effect of MRTX-EX185 on the HSQC spectrum of KRAS-GNP
100 µM U-15N KRAS-GNP
+ 200 µM MRTX-EX185
1H-15N HSQC, 800 MHz
pH 7.4, 10% dmso-d6, 298 K
N
100
110
120
130
[ppm]
H
11
10
9
8
7
6
[ppm]

Supplementary Note 1
Spectrum #17: Effect of AMG510 on the HSQC spectrum of KRAS(G12D)-GNP

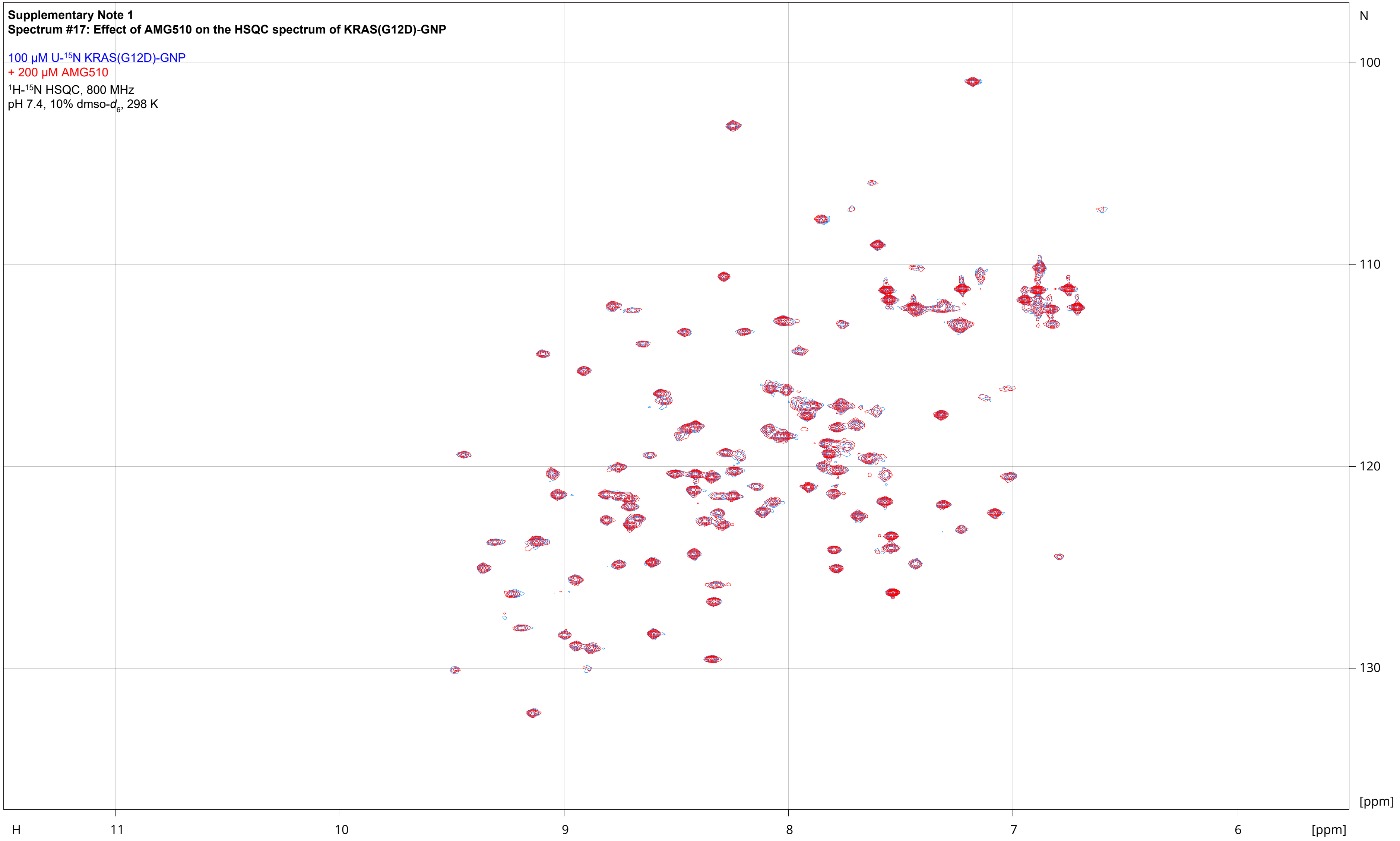

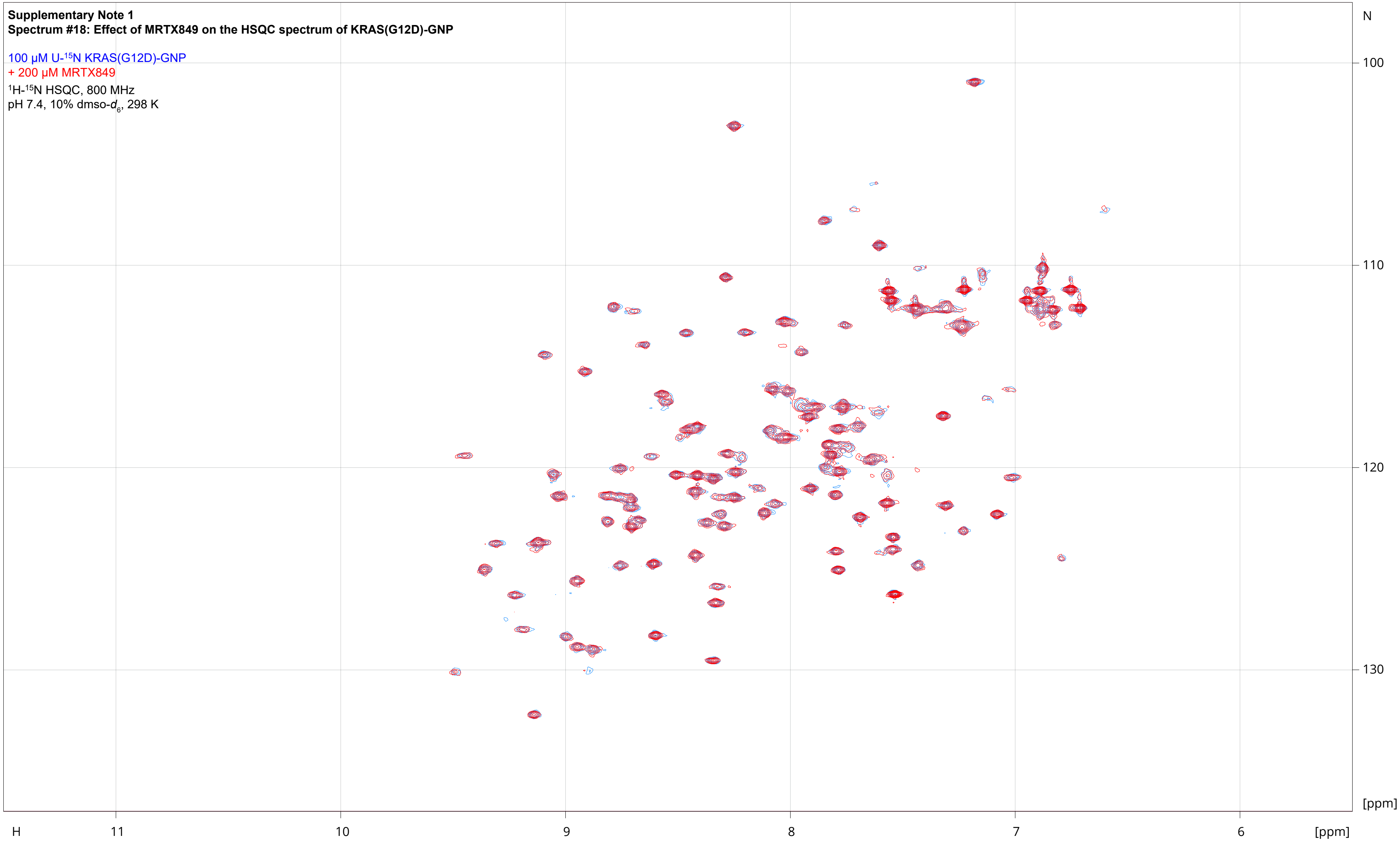
Supplementary Note 1
Spectrum #18: Effect of MRTX849 on the HSQC spectrum of KRAS(G12D)-GNP
100 µM U-15N KRAS(G12D)-GNP
+ 200 µM MRTX849
1H-15N HSQC, 800 MHz
pH 7.4, 10% dmso-d6, 298 K
N
100
110
120
130
[ppm]
H
11
10
9
8
7
6
[ppm]

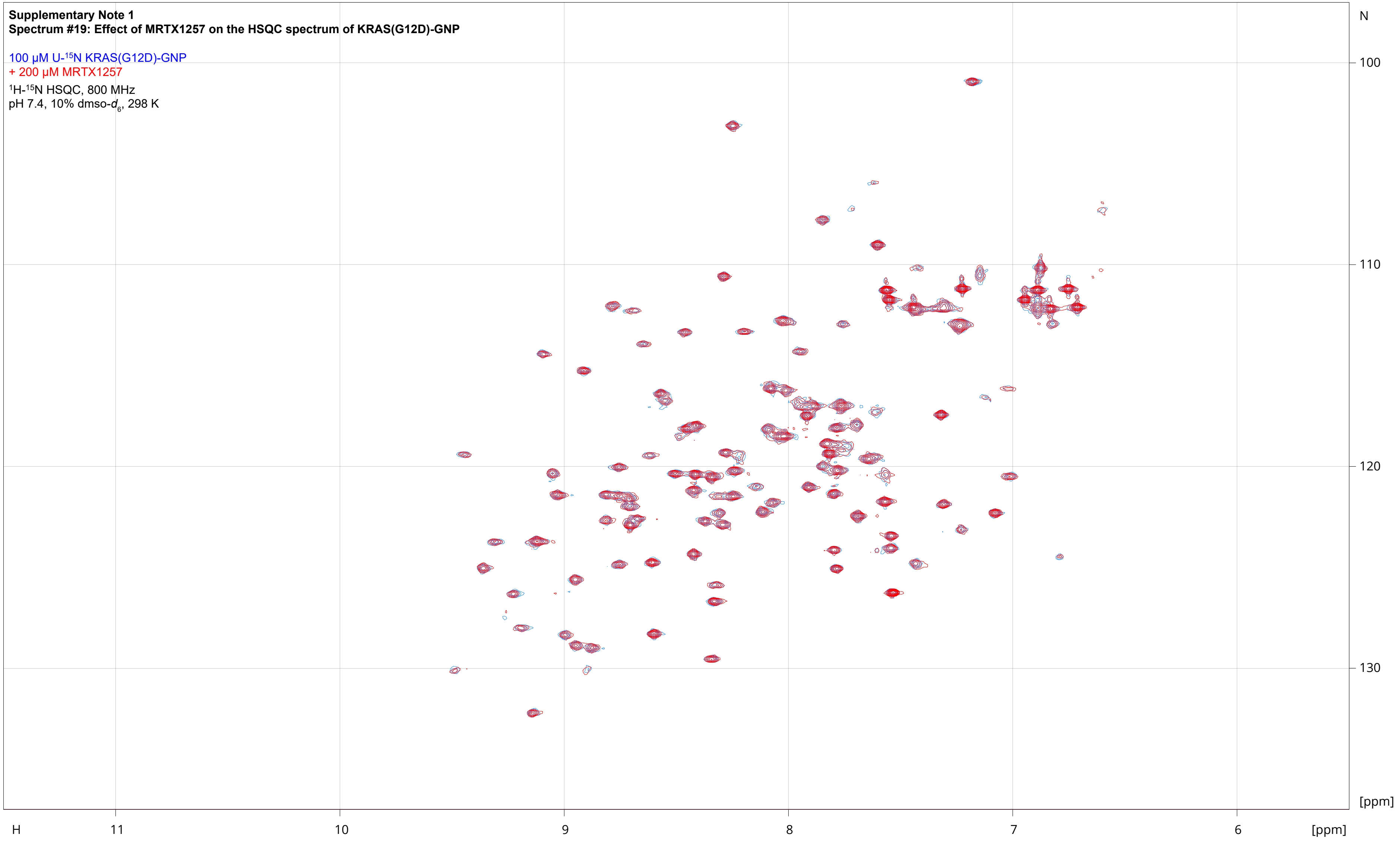
Supplementary Note 1
Spectrum #19: Effect of MRTX1257 on the HSQC spectrum of KRAS(G12D)-GNP
100 µM U-15N KRAS(G12D)-GNP
+ 200 µM MRTX1257
1H-15N HSQC, 800 MHz
pH 7.4, 10% dmso-d6, 298 K
N
100
110
120
130
[ppm]
H
11
10
9
8
7
6
[ppm]

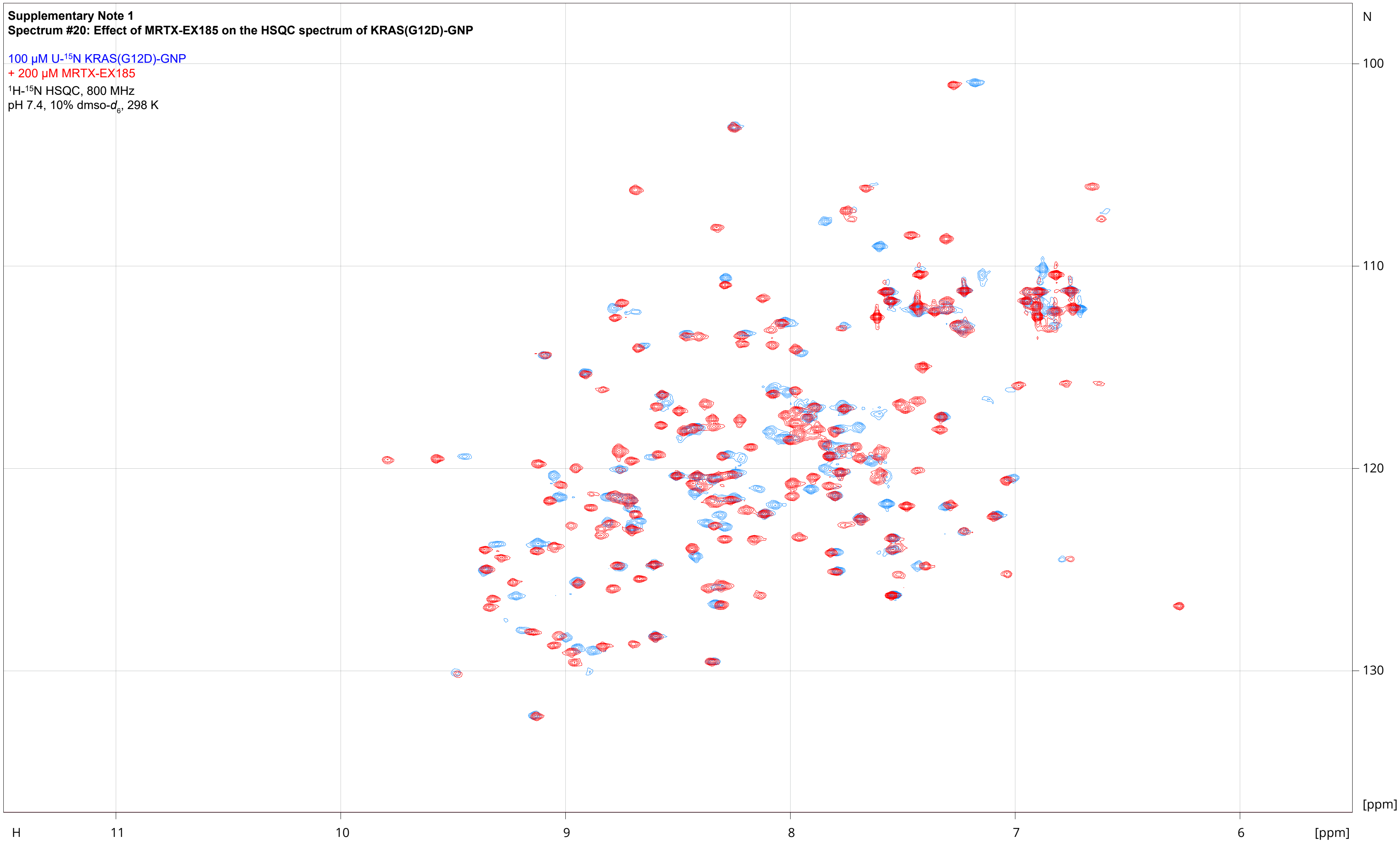

Supplementary Note 1
Spectrum #20: Effect of MRTX-EX185 on the HSQC spectrum of KRAS(G12D)-GNP
100 µM U-15N KRAS(G12D)-GNP
+ 200 µM MRTX-EX185
1H-15N HSQC, 800 MHz
pH 7.4, 10% dmso-d6, 298 K
N
100
110
120
130
[ppm]
H
11
10
9
8
7
6
[ppm]

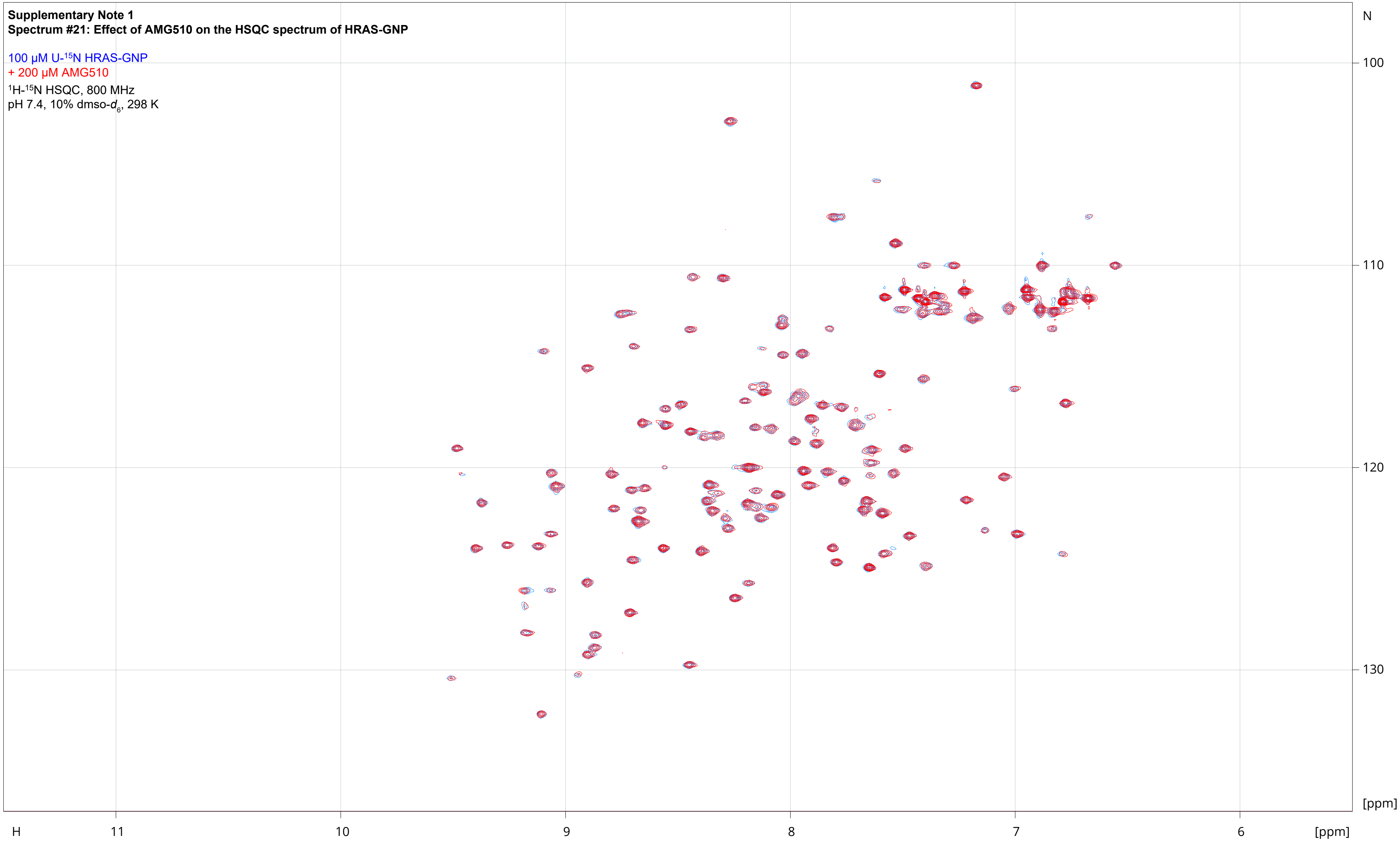
Supplementary Note 1
Spectrum #21: Effect of AMG510 on the HSQC spectrum of HRAS-GNP
100 µM U-15N HRAS-GNP
+ 200 µM AMG510
1H-15N HSQC, 800 MHz
pH 7.4, 10% dmso-d6, 298 K
N
100
110
120
130
[ppm]
H
11
10
9
8
7
6
[ppm]

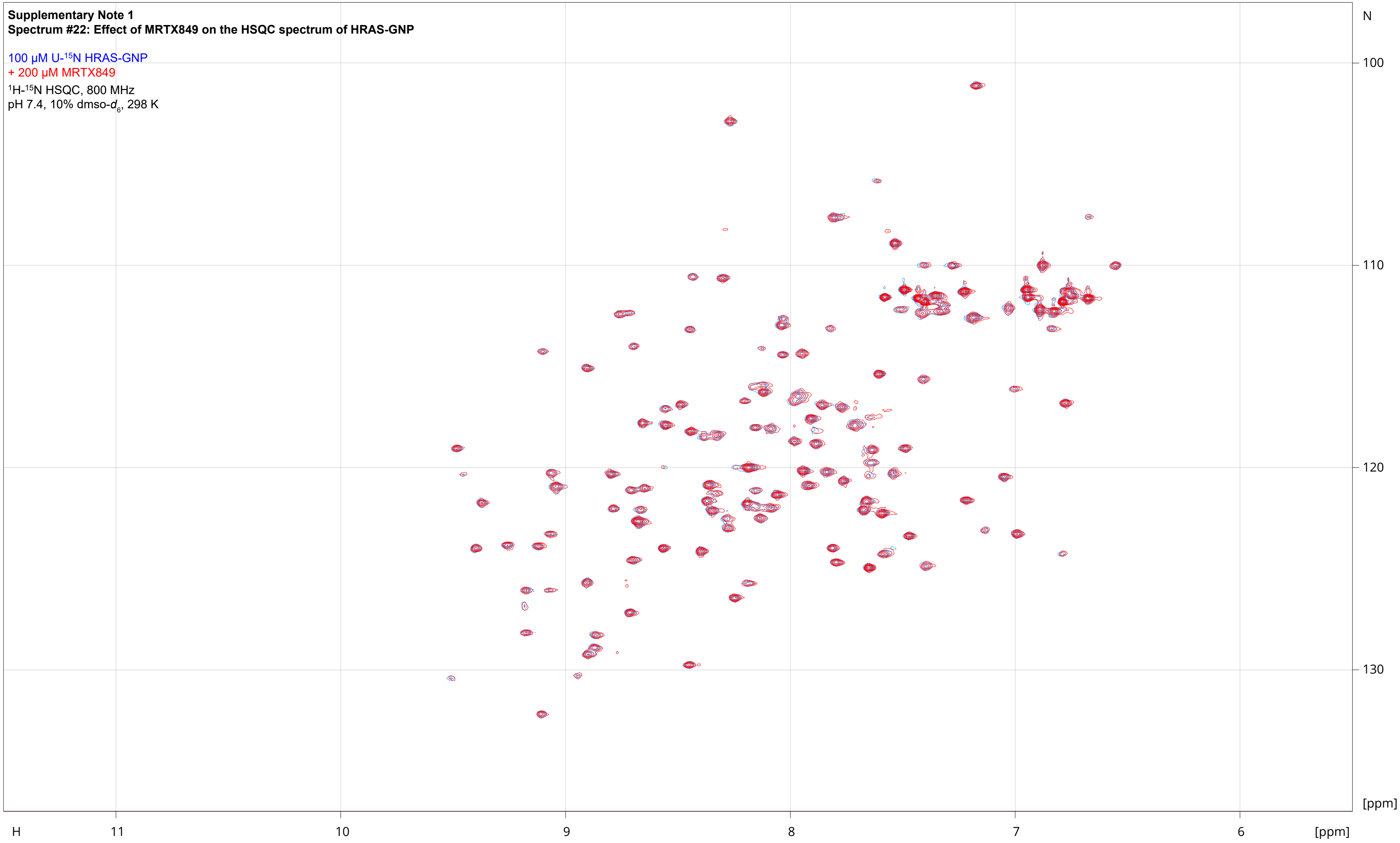
Supplementary Note 1
Spectrum #22: Effect of MRTX849 on the HSQC spectrum of HRAS-GNP
100 µM U-15N HRAS-GNP
+ 200 µM MRTX849
1H-15N HSQC, 800 MHz
pH 7.4, 10% dmso-d6, 298 K
N
100
110
120
130
[ppm]
H
11
10
9
8
7
6
[ppm]

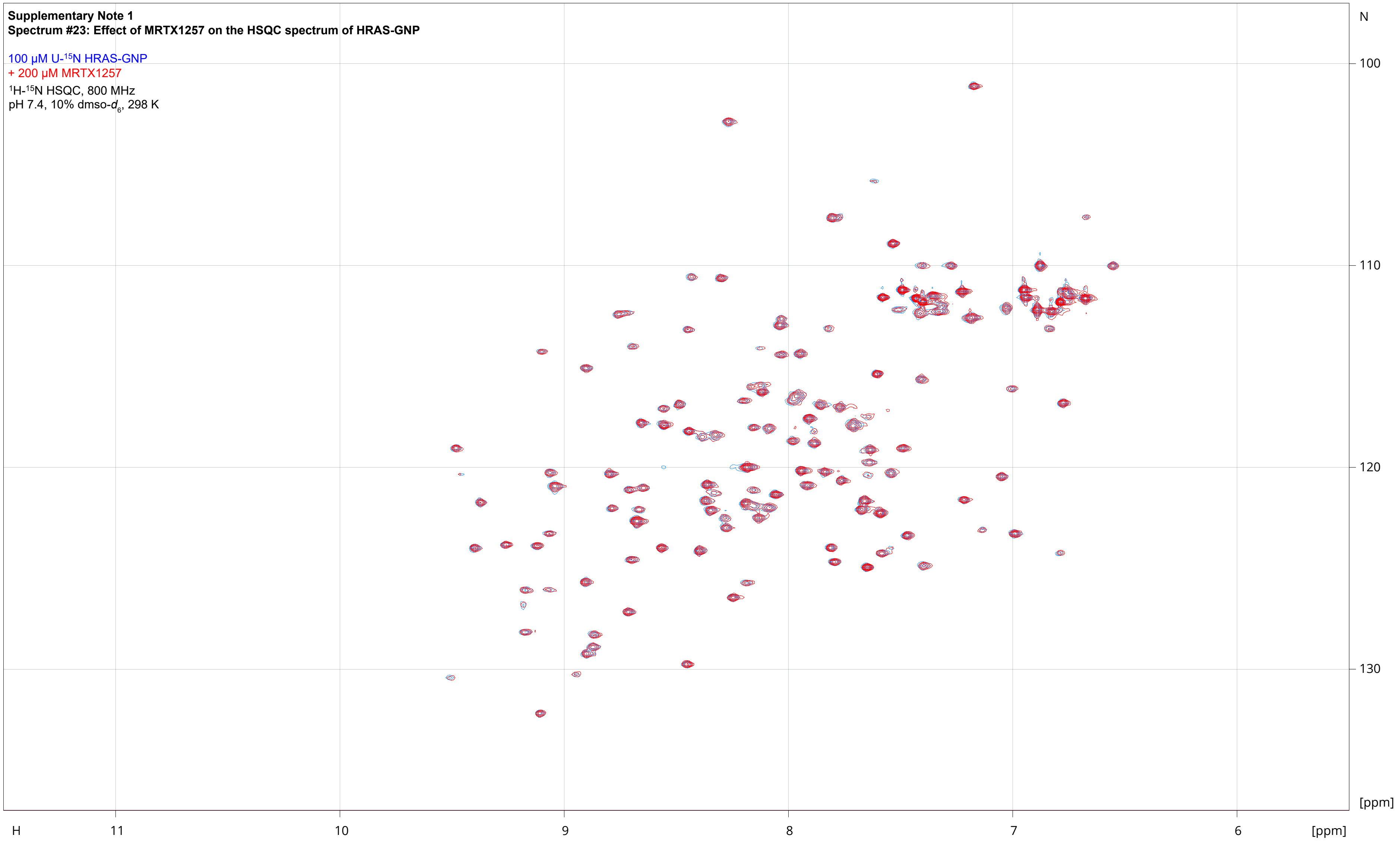
Supplementary Note 1
Spectrum #23: Effect of MRTX1257 on the HSQC spectrum of HRAS-GNP
100 µM U-15N HRAS-GNP
+ 200 µM MRTX1257
1H-15N HSQC, 800 MHz
pH 7.4, 10% dmso-d6, 298 K
N
100
110
120
130
[ppm]
H
11
10
9
8
7
6
[ppm]

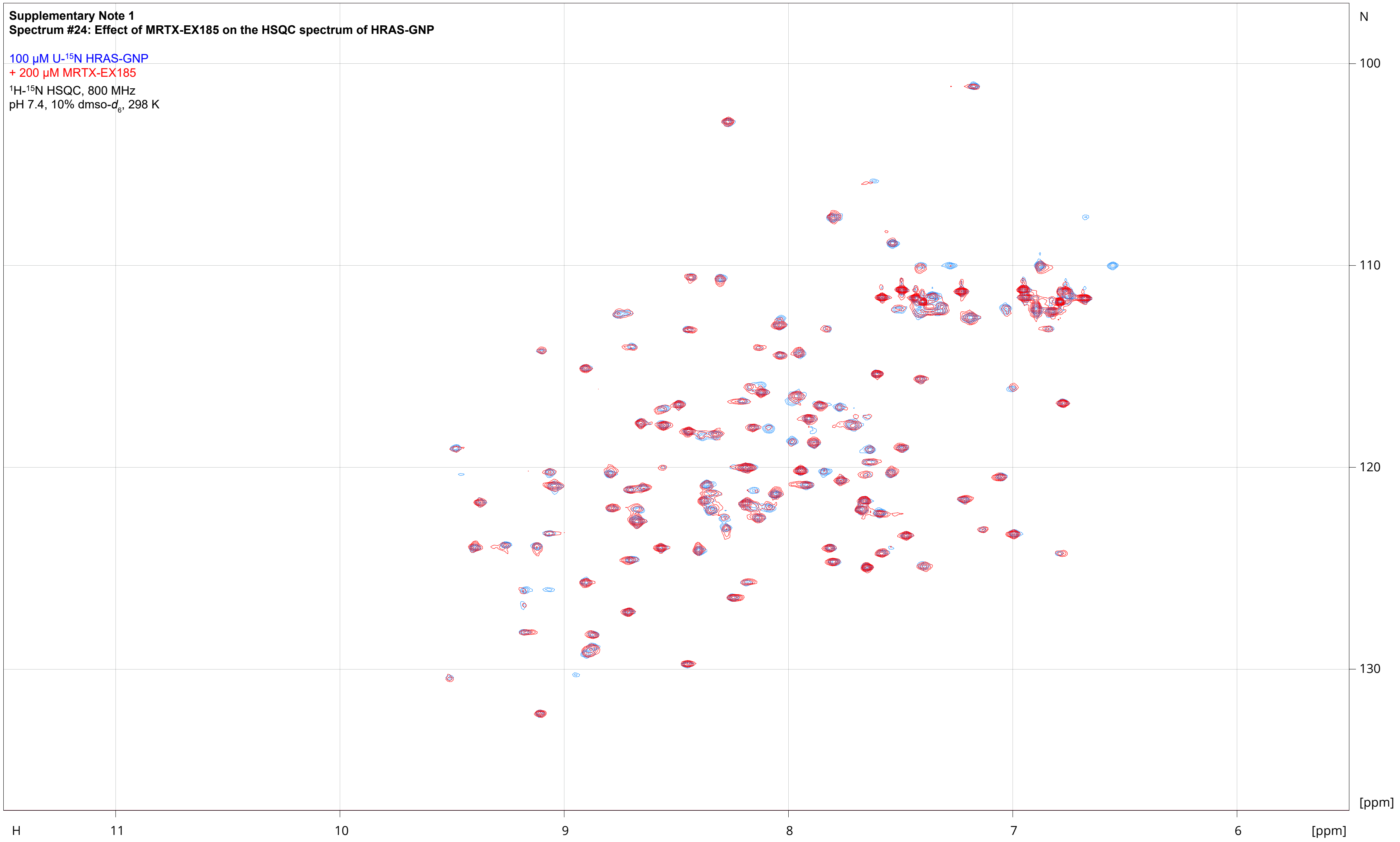
Supplementary Note 1
Spectrum #24: Effect of MRTX-EX185 on the HSQC spectrum of HRAS-GNP
100 µM U-15N HRAS-GNP
+ 200 µM MRTX-EX185
1H-15N HSQC, 800 MHz
pH 7.4, 10% dmso-d6, 298 K
N
100
110
120
130
[ppm]
H
11
10
9
8
7
6
[ppm]

Supplementary Note 1

Spectrum #25: Slow k_{off} of ligand binding determined from HSQC spectra of sub-stoichiometrically ligated proteins

^{1}H-^{15}N HSQC, 800 MHz

pH 7.4, 10% dmso-d_6, 298 K

Superimposed spectra showing G77

● dmso ● 2 equiv ligand ● 0.3 equiv ligand

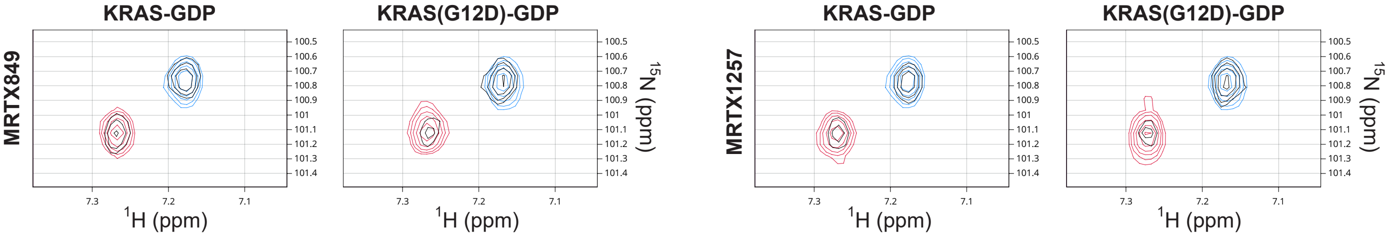

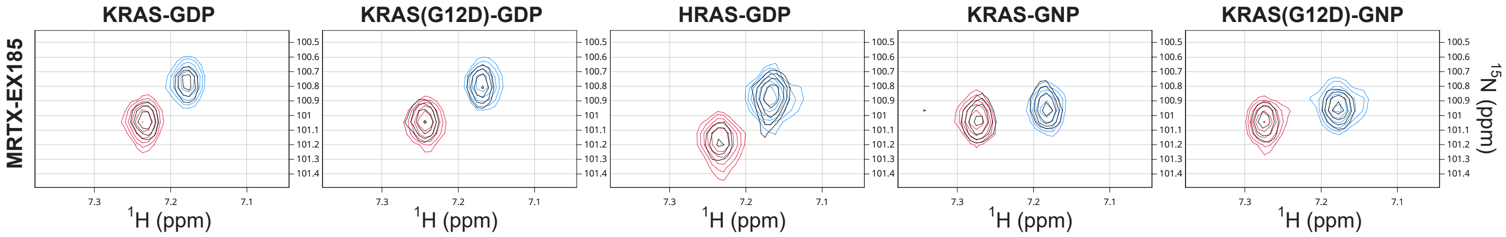

● dmso ● 2 equiv ligand ● 0.3 equiv ligand

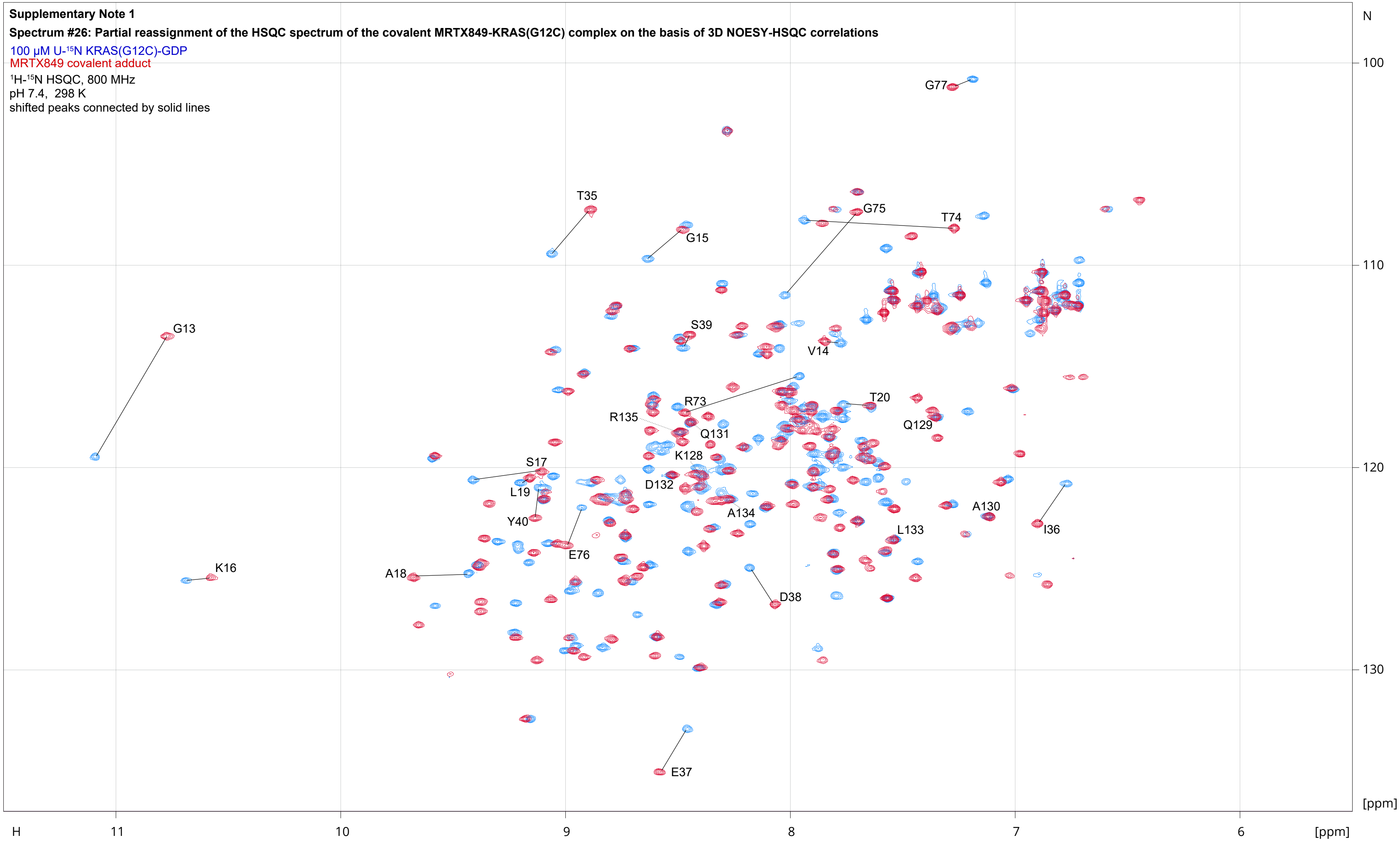

Supplementary Note 1
Spectrum #26: Partial reassignment of the HSQC spectrum of the covalent MRTX849-KRAS(G12C) complex on the basis of 3D NOESY-HSQC correlations
100 μM U-15N KRAS(G12C)-GDP
MRTX849 covalent adduct
1H-15N HSQC, 800 MHz
pH 7.4, 298 K
shifted peaks connected by solid lines
N
100
110
120
130
[ppm]
H
11
10
9
8
7
6
[ppm]
G77
T35
G75
T74
G15
G13
S39
V14
R73
T20
R135
Q131
Q129
K128
S17
D132
L19
A130
Y40
A134
L133
I36
E76
K16
A18
D38
E37

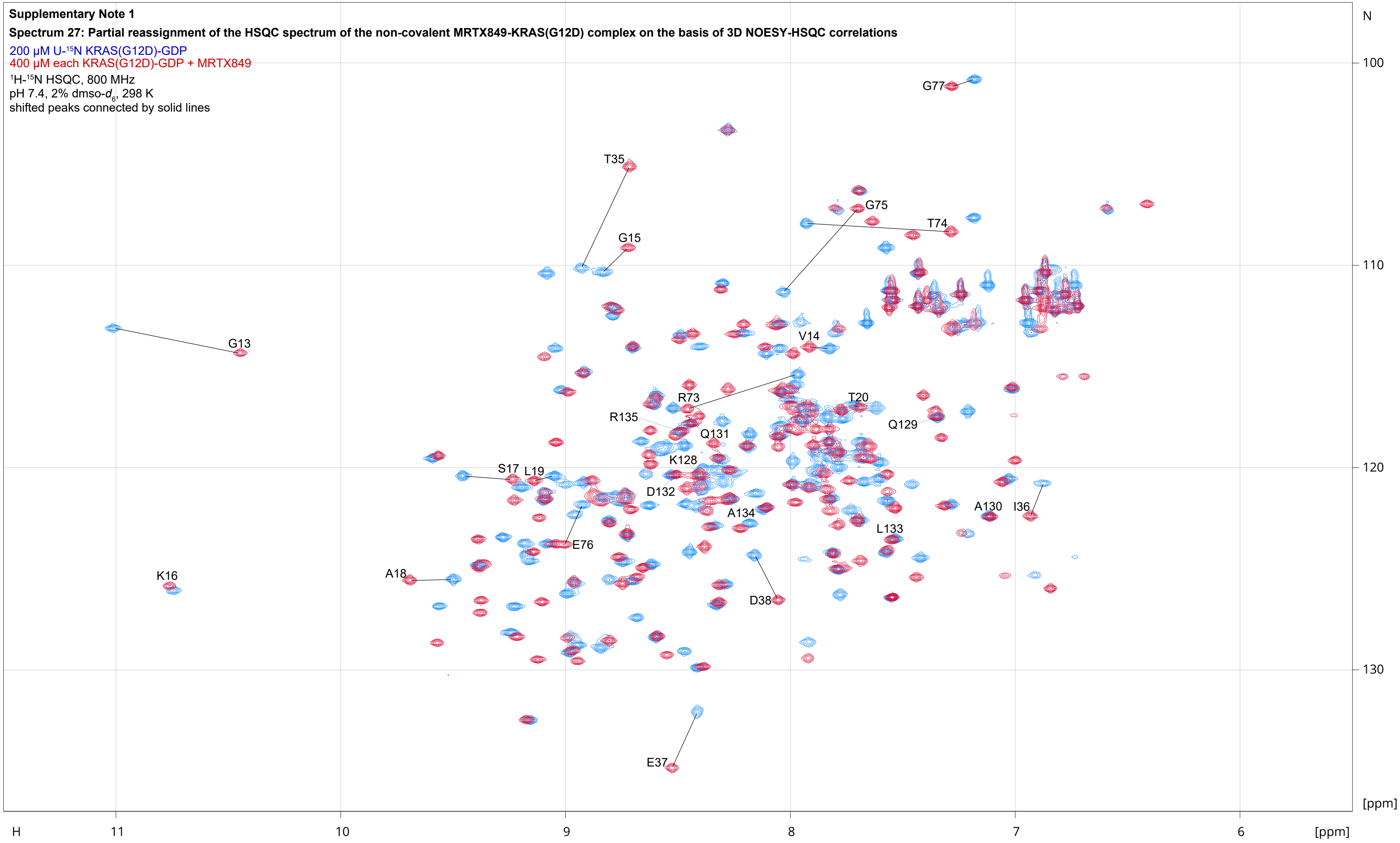
Supplementary Note 1
Spectrum 27: Partial reassignment of the HSQC spectrum of the non-covalent MRTX849-KRAS(G12D) complex on the basis of 3D NOESY-HSQC correlations
200 µM U-15N KRAS(G12D)-GDP
400 µM each KRAS(G12D)-GDP + MRTX849
1H-15N HSQC, 800 MHz
pH 7.4, 2% dmso-d6, 298 K
shifted peaks connected by solid lines
N
100
110
120
130
[ppm]
H
11
10
9
8
7
6
[ppm]
G77
T35
G75
T74
G15
G13
V14
R73
T20
R135
Q131
Q129
K128
S17
L19
D132
A134
A130
I36
L133
E76
K16
A18
D38
E37

Supplementary Note 1

Spectrum #28: Partial reassignment of the HSQC spectrum of the non-covalent MRTX-EX185-KRAS(G12D) complex on the basis of 3D NOESY-HSQC correlations

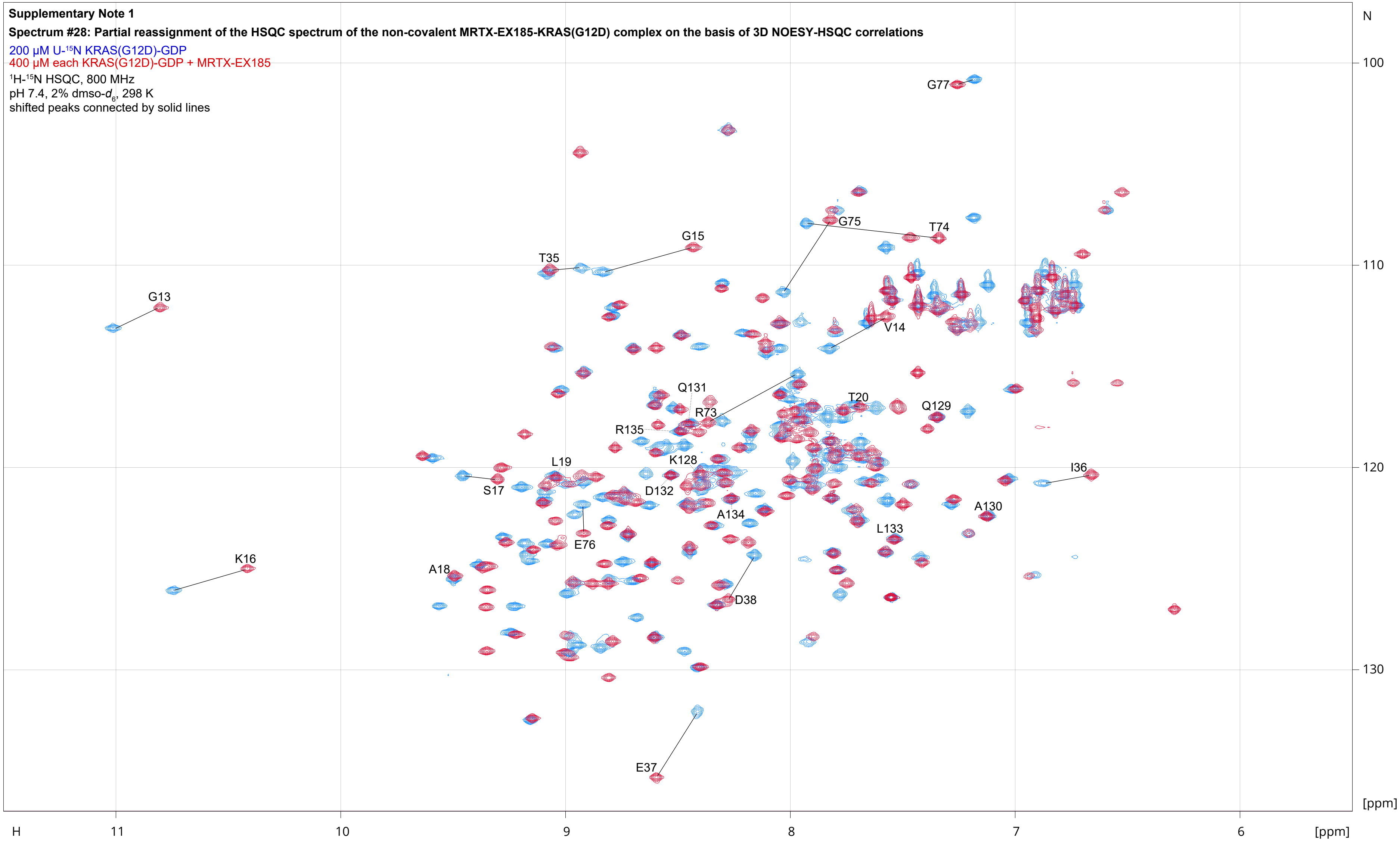

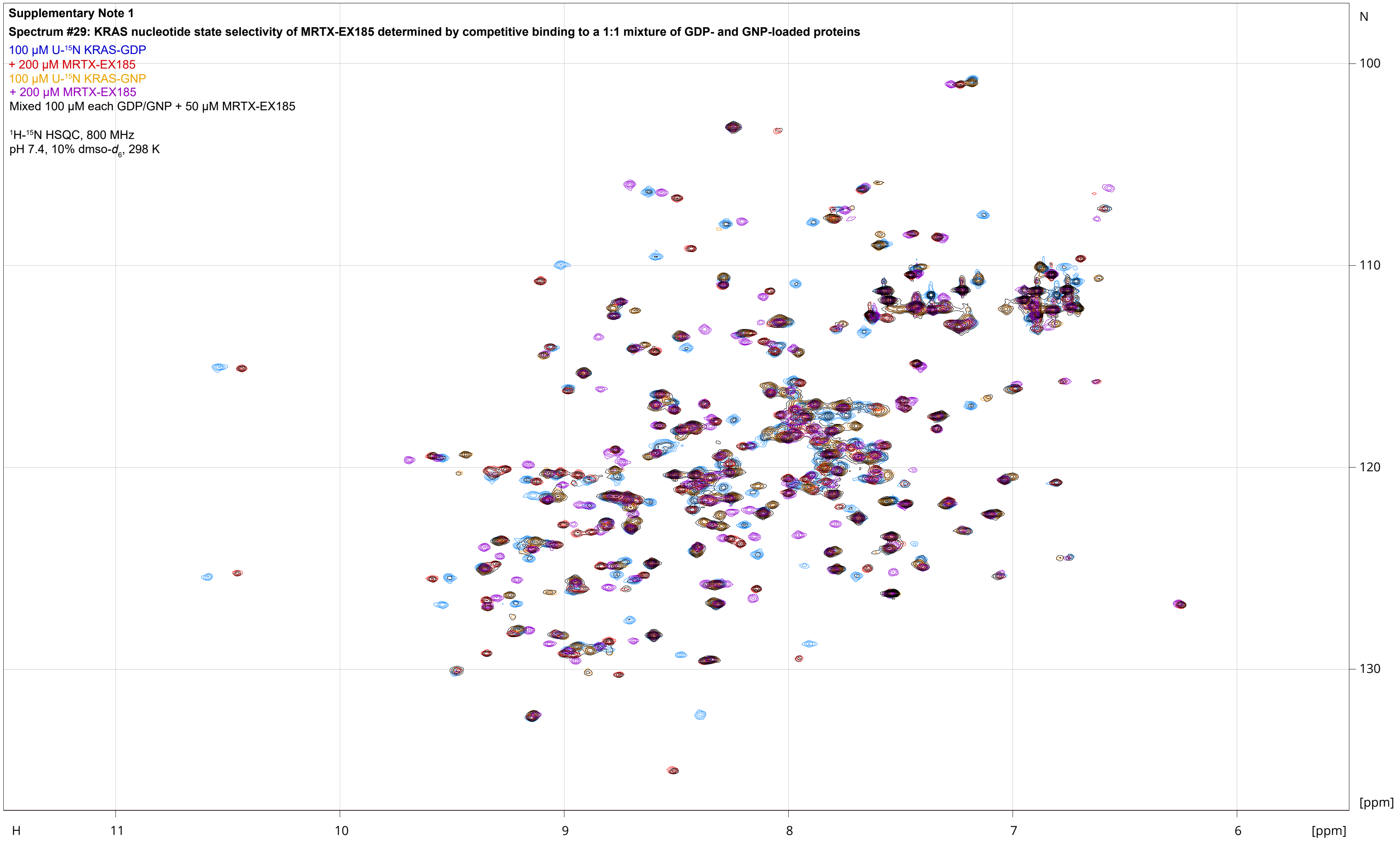

Supplementary Note 1
Spectrum #29: KRAS nucleotide state selectivity of MRTX-EX185 determined by competitive binding to a 1:1 mixture of GDP- and GNP-loaded proteins
100 µM U-15N KRAS-GDP
+ 200 µM MRTX-EX185
100 µM U-15N KRAS-GNP
+ 200 µM MRTX-EX185
Mixed 100 µM each GDP/GNP + 50 µM MRTX-EX185
1H-15N HSQC, 800 MHz
pH 7.4, 10% dmso-d6, 298 K
N
100
110
120
130
[ppm]
H
11
10
9
8
7
6
[ppm]

Supplementary Note 1

Spectrum #30: KRAS(G12D) nucleotide state selectivity of MRTX-EX185 determined by competitive binding to a 1:1 mixture of GDP- and GNP-loaded proteins

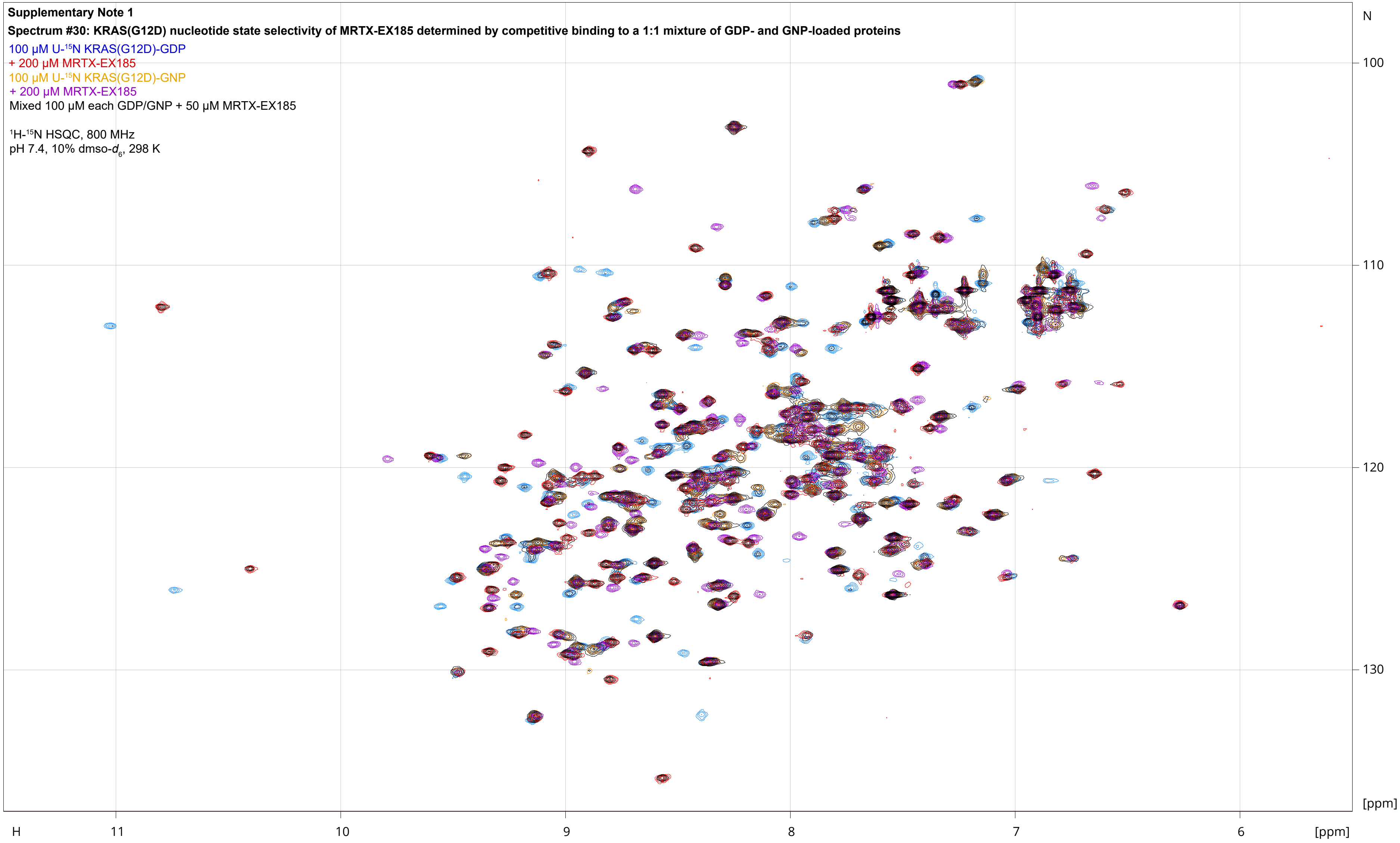

Supplementary Note 2 – Chemical synthesis and characterization

General information. Solvents were purchased from Sigma Aldrich or Fisher Scientific. Solvents referred to as “dry” were either collected from a solvent system containing mol sieves or purchased anhydrous from suppliers. NanoBRET 590 SE was obtained from Promega Corp. Madison, WI. Flash column chromatography was performed with a Combiflash Rf+ (Teledyne Isco) on silica. Preparative HPLC was performed with a 30x250 mm 5 µM C18 column (Waters Xbridge). High resolution ESI MS data was obtained from LC/MS with a C18 column (Waters Acquity UPLC and Xevo G2-XS QTof). ^{1}H and ^{19}F NMR spectra were recorded on a Bruker Avance 400 MHz spectrometer or a Bruker Ascend 400 MHz spectrometer. Chemical shifts (δ) are quoted in parts per million (ppm) and referenced to the residual solvent peak. Multiplicities are denoted as s-singlet, d-doublet, t-triplet, q-quartet and quin-quintet and derivatives thereof (br denotes a broad resonance peak). Coupling constants are given in Hz and round to the nearest 0.1 Hz.

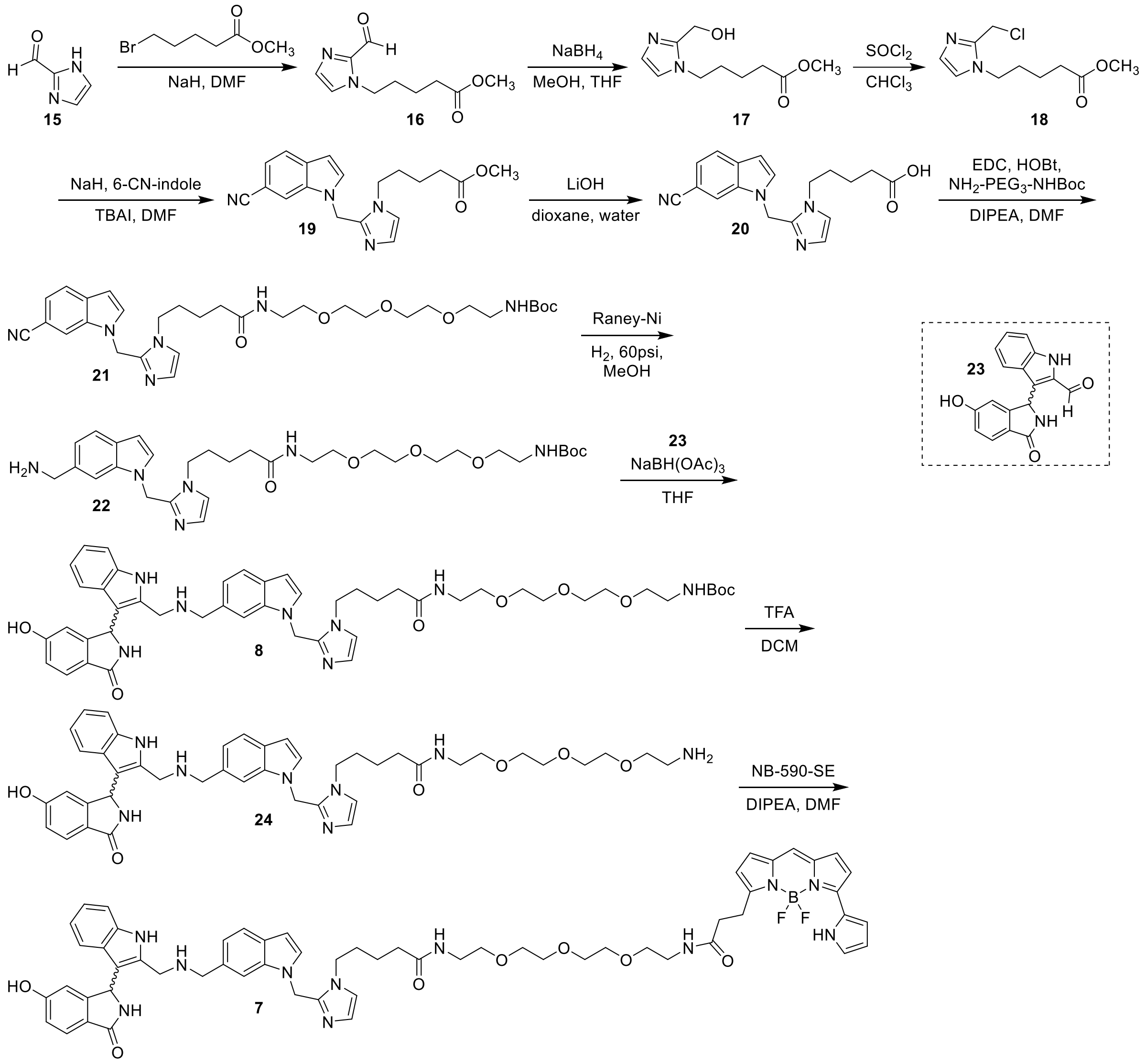

Synthesis of pan RAS BRET probe 7.

Methyl 5-(2-formyl-1H-imidazol-1-yl)pentanoate (16). To a suspension of 1H-imidazole-2-carbaldehyde (**15**, 1.00 g, 10.4 mmol) in DMF (20 mL) chilled with an ice bath was added sodium hydride (60% oil dispersion, 500 mg, 12.5 mmol). The mixture was warmed to ambient temperature and methyl 5-bromopentanoate (2.44 g, 12.5 mmol) was added in DMF (5mL). The mixture stirred for 4h at ambient temperature. The reaction was quenched with water, diluted with brine, and extracted with chloroform/isopropanol (3:1). The organic layers were combined,

dried with sodium sulfate, filtered, concentrated, and purified with silica gel chromatography to afford the desired product (0.92 g, 42%) as a colorless oil. ^{1}H NMR (400 MHz, Methanol-d4) δ 9.73 (s, 1H), 7.53 (s, 1H), 7.28 (s, 1H), 7.10 (s, rotamer), 6.88 (s, rotamer), 4.46 (t, *J* = 7.2 Hz, 2H), 4.18 (t, rotamer), 3.66 (s, 3H), 2.38 (t, *J* = 7.2, 2H), 1.90 – 1.77 (m, 2H), 1.70 – 1.55 (m, 2H); ^{13}C NMR (101 MHz, MeOD) δ 182.15, 175.30, 144.39, 131.84, 128.49, 126.99 (rotamer), 122.23 (rotamer), 52.04, 34.15 (rotamer), 33.98, 31.36, 31.27 (rotamer), 22.95 (rotamer), 22.66. ESI MS m/z 211 [M + 1]+.

Methyl 5-(2-(hydroxymethyl)-1H-imidazol-1-yl)pentanoate (17). To a solution of methyl 5-(2-formyl-1H-imidazol-1-yl)pentanoate (0.92 g, 4.4 mmol) in methanol/tetrahydrofuran (1:1, 50 mL) chilled with an ice bath was added sodium borohydride (0.20 g, 5.3 mmol). The mixture stirred for 30 min. The reaction was quenched with HCl (3 mL, 2M) and then the pH was adjusted to 8. The mixture was concentrated with celite and purified with silica gel chromatography to afford the desired product (0.53 g, 57%) as a colorless oil. ^{1}H NMR (400 MHz, Methanol-d4) δ 7.28 (s, 1H), 7.08 (s, 1H), 4.73 (s, 2H), 4.19 – 4.09 (m, 2H), 3.67 (s, 3H), 2.40 (td, *J* = 7.5, 2.2 Hz, 2H), 1.95 – 1.80 (m, 2H), 1.73 – 1.58 (m, 2H); ^{13}C NMR (101 MHz, MeOD) δ 174.01, 146.67, 123.57, 121.14, 54.84, 50.68, 45.90, 32.66, 29.58, 21.49; HRMS (ESI+) calcd for $C_{10}H_{16}N_2O_3$ [M + H]+ m/z 213.1239, found 213.1223.

Methyl 5-(2-(chloromethyl)-1H-imidazol-1-yl)pentanoate (18) . To a solution of methyl 5-(2-(hydroxymethyl)-1H-imidazol-1-yl)pentanoate (0.53 g, 2.5 mmol) in chloroform was added thionyl chloride (3.0 g, 25 mmol). The mixture stirred for 1 h at ambient temperature and heated

to 75°C for 1 h. The reaction was concentrated, and the residue was suspended in ether. Filtration afforded the crude product (0.64 g) as a white solid. ESI MS m/z 231 [M + 1]+.

Methyl 5-(2-((6-cyano-1H-indol-1-yl)methyl)-1H-imidazol-1-yl)pentanoate (19). To a solution of 1H-indole-6-carbonitrile (200 mg, 1.41 mmol) in DMF (20 mL) chilled with an ice bath was added sodium hydride (84 mg, 2.1 mmol, 60%), methyl 5-(2-(chloromethyl)-1H-imidazol-1-yl)pentanoate (0.38 g, 1.4 mmol), and tetrabutylammonium iodide (52 mg, 0.14 mmol). The mixture stirred at 0 °C for 15 min and ambient temperature for 2 h. The reaction was diluted with ethyl acetate and washed with water. The organic layers were combined, dried with sodium sulfate, filtered, concentrated, and purified with silica gel chromatography to afford the desired product (370 mg, 78%) as an orange oil. ^{1}H NMR (400 MHz, Methanol-d4) δ 7.95 (s, 1H), 7.71 (d, *J* = 8.2 Hz, 1H), 7.52 – 7.46 (m, 1H), 7.33 (d, *J* = 8.0, 1H), 7.15 (s, 1H), 7.03 (s, 1H), 6.65 (s, 1H), 5.56 (s, 2H), 3.88 (t, *J* = 7.0 Hz, 2H), 3.61 (s, 3H), 2.06 (t, *J* = 7.0 Hz, 2H), 1.38 – 1.20 (m, 4H); ^{13}C NMR (101 MHz, MeOD) δ 173.72, 142.41, 135.11, 132.19, 131.64, 126.70, 122.12, 121.61, 121.56, 119.99, 114.64, 103.76, 102.71, 50.65, 45.61, 42.16, 32.54, 29.58, 21.30; HRMS (ESI+) calcd for $C_{19}H_{20}N_4O_2$ [M + H]+ m/z 337.1664, found 337.1643; HPLC 97.8% (AUC at 286 nm) 3.10 min (Synergi Max-RP, water/ACN, 0.02%TFA).

5-(2-((6-Cyano-1H-indol-1-yl)methyl)-1H-imidazol-1-yl)pentanoic acid (20). To a solution of methyl 5-(2-((6-cyano-1H-indol-1-yl)methyl)-1H-imidazol-1-yl)pentanoate (0.37 g, 1.1 mmol) in dioxane (10 mL) was added lithium hydroxide (0.13 g, 5.5 mmol) and water (1 mL). The reaction was heated to 40 °C for 2 h. The mixture diluted with water, the pH was adjusted to 3

with HCl, and extracted with chloroform/isopropanol (3:1). Concentration of the organic layer afforded the crude product (0.30 g) as a white solid. ESI MS m/z 323 [M + 1]+.

***tert*-Butyl (17-(2-((6-cyano-1H-indol-1-yl)methyl)-1H-imidazol-1-yl)-13-oxo-3,6,9-trioxa-12-azaheptadecyl)carbamate (21).** To a solution of 5-(2-((6-cyano-1H-indol-1-yl)methyl)-1H-imidazol-1-yl)pentanoic acid (0.30 g, 0.93 mmol) in DMF (10 mL) was added tert-butyl (2-(2-(2-(2-aminoethoxy)ethoxy)ethoxy)ethyl)carbamate (0.33 g, 1.1 mmol), hydroxybenzotriazole (0.28 g, 1.9 mmol), 1-ethyl-3-(3′-dimethylaminopropyl)carbodiimide, HCl (0.36 g, 1.9 mmol), and diisopropylamine (0.36 g, 2.8 mmol). The reaction was heated to 60 °C for 1 h. The reaction was diluted with ethyl acetate and washed with water. The organic layers were combined, dried with sodium sulfate, filtered, concentrated, and purified with silica gel chromatography to afford the desired product (525 mg, 94%) as a light brown oil. ^{1}H NMR (400 MHz, Methanol-d4) δ 7.96 (s, 1H), 7.89 (s, 1H), 7.73 (d, *J* = 8.5, 1H), 7.54 – 7.48 (m, 1H), 7.35 (d, *J* = 8.2 Hz, 1H), 7.17 (s, 1H), 7.03 (s, 1H), 6.67 (s, 1H), 6.60 (s, 1H), 5.58 (s, 2H), 3.90 (t, *J* = 7.3 Hz, 2H), 3.67 – 3.56 (m, 8H), 3.56 – 3.45 (m, 4H), 3.36 (s, 3H), 3.26 – 3.17 (m, 2H), 2.02 (t, *J* = 6.6 Hz, 2H), 1.43 (s, 9H), 1.40 – 1.24 (m, 4H); ^{13}C NMR (101 MHz, MeOD) δ 173.94, 157.07, 142.37, 135.14, 132.24, 131.74, 126.72, 122.12, 121.65, 121.55, 120.02, 114.69, 103.70, 102.71, 78.69, 70.17, 70.14, 69.80, 69.76, 69.66, 69.13, 48.47, 45.65, 42.20, 39.99, 39.86, 38.93, 34.70, 29.78, 27.38, 22.25; HRMS (ESI+) calcd for $C_{31}H_{44}N_6O_6$ [M + H]+ m/z 597.3400, found 597.3384; HPLC 99.0% (AUC at 286 nm) 3.53 min (Synergi Max-RP, water/ACN, 0.02%TFA).

***tert*-Butyl (17-(2-((6-(aminomethyl)-1H-indol-1-yl)methyl)-1H-imidazol-1-yl)-13-oxo-3,6,9-trioxa-12-azaheptadecyl)carbamate (22).** To a solution of tert-butyl (17-(2-((6-cyano-1H-

indol-1-yl)methyl)-1H-imidazol-1-yl)-13-oxo-3,6,9-trioxa-12-azaheptadecyl)carbamate (0.52 g, 0.88 mmol) in ammonia in methanol (7 N, 20 mL) was added a scoop of Rainey Nickel suspended in water. The reaction was charged with hydrogen (60 psi) and stirred at ambient temperature for 18 h. After degassing with nitrogen, the mixture was filtered over celite. The filtrate was concentrated to afford the crude product (0.49 g) as a colorless oil. ESI MS m/z 601 [M + 1]+.

***tert*-Butyl (17-(2-((6-((((3-(6-hydroxy-3-oxoisoindolin-1-yl)-1H-indol-2-yl)methyl)amino)methyl)-1H-indol-1-yl)methyl)-1H-imidazol-1-yl)-13-oxo-3,6,9-trioxa-12-azaheptadecyl)carbamate (8).** To a suspension of tert-butyl (17-(2-((6-(aminomethyl)-1H-indol-1-yl)methyl)-1H-imidazol-1-yl)-13-oxo-3,6,9-trioxa-12-azaheptadecyl)carbamate (0.45 g, 0.75 mmol) in THF (10 mL) was added 3-(6-hydroxy-3-oxoisoindolin-1-yl)-1H-indole-2-carbaldehyde (**23**, 0.22 g, 0.75 mmol)[1]. The reaction stirred at ambient temperature for 1 h. Sodium triacetoxyborohydride (0.48 g, 2.3 mmol) was added and stirred at ambient temperature for 3 h. The reaction was diluted with methanol, celite was added, concentrated, and purified with silica gel chromatography to afford the desired product (0.53 g, 80%) as a brown foam. ^{1}H NMR (400 MHz, Methanol-d4) δ 7.71 (d, *J* = 8.3 Hz, 1H), 7.62 (d, *J* = 8.3 Hz, 2H), 7.38 (d, *J* = 8.2 Hz, 1H), 7.27 (s, 1H), 7.18 (d, *J* = 8.2 Hz, 1H), 7.14 – 7.04 (s, 2H), 7.00 – 6.87 (m, 2H), 6.87 – 6.77 (m, 1H), 6.77 – 6.73 (m, 2H), 6.54 (s, 1H), 5.95 (s, 1H), 5.48 (s, 2H), 4.55 – 4.32 (m, 2H), 4.24 (s, 2H), 3.92 – 3.79 (m, 2H), 3.63 – 3.49 (m, 8H), 3.46 (t, *J* = 5.6 Hz, 2H), 3.40 (t, *J* = 5.6 Hz, 2H), 3.27 – 3.14 (m, 4H), 2.22 – 1.72 (m, 6H), 1.42 (s, 9H), 1.32 – 1.08 (m, 4H); ^{13}C NMR (101 MHz, MeOD) δ 175.62, 174.06, 171.66, 161.97, 157.02, 150.94, 142.98, 136.69, 136.20, 129.32, 128.99, 128.73, 126.49, 126.38, 125.44, 124.47, 122.76, 122.55, 121.41, 121.29, 120.77,

119.39, 118.80, 115.88, 111.29, 111.04, 110.77, 109.77, 102.09, 78.70, 70.10, 69.76, 69.72, 69.62, 69.04, 52.85, 51.80, 45.65, 41.80, 41.33, 39.84, 38.93, 34.65, 29.76, 27.38, 22.21, 20.62; HRMS (ESI+) calcd for $C_{48}H_{60}N_8O_8$ [M + H]+ m/z 877.4612, found 877.4607; HPLC 96.7% (AUC at 266 nm) 2.79 min (Synergi Max-RP, water/ACN, 0.02%TFA).

N-(2-(2-(2-(2-Aminoethoxy)ethoxy)ethoxy)ethyl)-5-(2-((6-((((3-(6-hydroxy-3-oxoisoindolin-1-yl)-1H-indol-2-yl)methyl)amino)methyl)-1H-indol-1-yl)methyl)-1H-imidazol-1-yl)pentanamide (24). To a solution of tert-butyl (17-(2-((6-((((3-(6-hydroxy-3-oxoisoindolin-1-yl)-1H-indol-2-yl)methyl)amino)methyl)-1H-indol-1-yl)methyl)-1H-imidazol-1-yl)-13-oxo-3,6,9-trioxa-12-azaheptadecyl)carbamate (0.030 g, 0.034 mmol) in dichloromethane (10 mL) was added trifluoroacetic acid (1 mL). The reaction stirred at ambient temperature for 1.5 h. The mixture was concentrated to give crude product as a colorless oil. ESI MS m/z 777 [M + 1]+.

N-(15-(5,5-Difluoro-7-(1H-pyrrol-2-yl)-5H-5l4,6l4-dipyrrolo[1,2-c:2',1'-f][1,3,2]diazaborinin-3-yl)-13-oxo-3,6,9-trioxa-12-azapentadecyl)-5-(2-((6-((((3-(6-hydroxy-3-oxoisoindolin-1-yl)-1H-indol-2-yl)methyl)amino)methyl)-1H-indol-1-yl)methyl)-1H-imidazol-1-yl)pentanamide (7). To a solution of N-(2-(2-(2-(2-aminoethoxy)ethoxy)ethoxy)ethyl)-5-(2-((6-((((3-(6-hydroxy-3-oxoisoindolin-1-yl)-1H-indol-2-yl)methyl)amino)methyl)-1H-indol-1-yl)methyl)-1H-imidazol-1-yl)pentanamide (26 mg, 0.033 mmol) in DMF (2 mL) was added 2,5-dioxopyrrolidin-1-yl 3-(5,5-difluoro-7-(1H-pyrrol-2-yl)-5H-5l4,6l4-dipyrrolo[1,2-c:2',1'-f][1,3,2]diazaborinin-3-yl)propanoate (14 mg, 0.033 mmol) and diisopropylethylamine (34 mg, 0.27 mmol). The reaction was stirred at ambient temperature for 30 min. The mixture was diluted with methanol and purified by reverse phase preparative HPLC

to afford the desired product (38 mg, quant) as a purple solid. ^{1}H NMR (400 MHz, Methanol-d4) δ 7.71 (d, *J* = 8.2 Hz, 1H), 7.54 (d, *J* = 8.2 Hz, 1H), 7.46 (s, 1H), 7.32 (d, *J* = 8.6 Hz, 1H), 7.22 – 7.07 (m, 6H), 7.07 – 6.95 (m, 3H), 6.95 – 6.85 (m, 3H), 6.79 (t, *J* = 7.6 Hz, 1H), 6.75 – 6.64 (m, 2H), 6.47 (s, 1H), 6.37 – 6.27 (m, 2H), 5.92 (s, 1H), 5.41 (s, 2H), 4.00 (s, 2H), 3.89 (s, 2H), 3.72 (t, *J* = 7.5 Hz, 2H), 3.62 – 3.45 (m, 10H), 3.43-3.34 (m, 4H), 3.30 – 3.24 (d, 2H), 3.21 (t, *J* = 5.6 Hz, 2H), 2.63 (t, *J* = 7.8 Hz, 2H), 1.80 (t, *J* = 7.3 Hz, 2H), 1.25 – 1.13 (m, 2H), 1.13 – 1.01 (m, 2H); HRMS (ESI+) calcd for $C_{59}H_{64}BF_2N_{11}O_7$ [M + H]+ m/z 1088.5129, found 1088.5124; HPLC 95.9% (AUC at 580 nm) 3.36 min (Synergi Max-RP, water/ACN, 0.02%TFA).

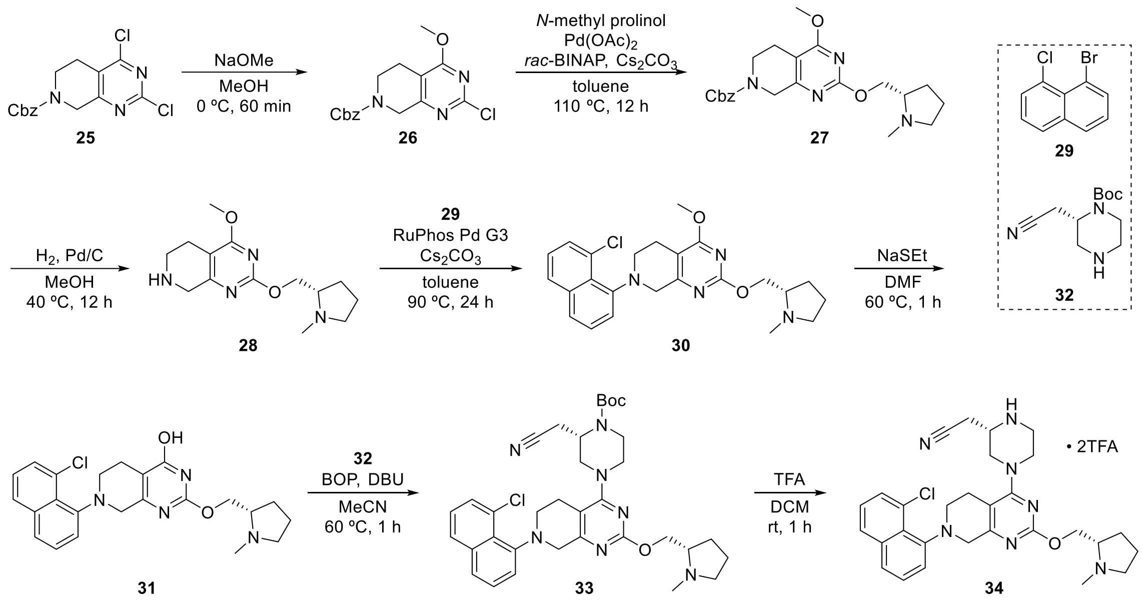

Preparation of piperazine precursor of MRTX849.

Synthesis of MRTX849 precursor was adapted from the published syntheses.[2,3]

Benzyl 2-chloro-4-methoxy-5,8-dihydropyrido[3,4-*d*]pyrimidine-7(6*H*)-carboxylate (26). A 250-mL round bottom flask equipped with a stir bar was charged with benzyl 2,4-dichloro-5,8-dihydropyrido[3,4-*d*]pyrimidine-7(6*H*)-carboxylate (**25**, 4.78 g, 14.1 mmol). Methanol (100 mL) was added. The resulting yellow suspension was cooled to 0 °C. Sodium methoxide (25 wt% in MeOH, 3.87 mL, 17.0 mmol) was added dropwise. The resulting mixture was stirred at 0 °C for 60 min, at which point the full conversion of **25** was complete as judged by TLC. The reaction mixture was neutralized by 2 N HCl and then concentrated. The residue was partitioned between EA (100 mL) and water (100 mL). The phases were separated, and the aqueous phase was extracted by EA (100 mL x 2). The organic solution was combined, washed by brine, and dried on anhydrous sodium sulfate. Upon concentration, the solid crude was triturated in ether, filtered, and dried in vacuum (50 mTorr) overnight. The title compound was obtained as a white powder (4.39 g, 13.1 mmol, 93% yield). ^{1}H NMR (400 MHz, Chloroform-*d*) δ 7.44 – 7.28 (m, 5H), 5.16

(s, 2H), 4.59 (s, 2H), 4.02 (s, 3H), 3.73 (t, *J* = 5.8 Hz, 2H), 2.65 (s, 2H). ^{13}C NMR (100 MHz, Chloroform-*d*) δ 168.7, 157.4, 155.2, 136.2, 128.6, 128.3, 128.1, 113.5, 67.7, 55.0, 47.6, 40.1, 21.2. TLC Rf 0.69 (50% EA-hexanes, UV). Accurate MS (ESI-TOF) calculated for $C_{16}H_{17}ClN_3O_3$ $[M + H]^+$ 334.0958, found 334.1016.

Benzyl (*S*)-4-methoxy-2-((1-methylpyrrolidin-2-yl)methoxy)-5,8-dihydropyrido[3,4-*d*]pyrimidine-7(6*H*)-carboxylate (27). A 100-mL round bottom flask equipped with a stir bar was charged with benzyl 2-chloro-4-methoxy-5,8-dihydropyrido[3,4-*d*]pyrimidine-7(6*H*)-carboxylate (**16**, 1.67 g, 5.00 mmol), (*S*)-(1-methylpyrrolidin-2-yl)methanol (1.15 g, 10.0 mmol, 2.0 equiv), palladium acetate (112 mg, 0.500 mmol, 0.10 equiv), *rac*-BINAP (623 mg, 1.00 mmol, 0.20 equiv), cesium carbonate (4.89 g, 15.0 mmol, 3.0 equiv). Sealed with a rubber septum, the flask was repeatedly deaerated and backfilled with argon three times. Toluene (25 mL) was added. The resulting mixture was stirred at 110 °C under argon atmosphere for 12 h, at which point the conversion of the limiting starting material, pyrimidine chloride, was complete as judged by LC-MS. The reaction mixture was directly partitioned between EA (100 mL) and water (100 mL). The two phases were separated and the aqueous was further extracted with EA (100 mL x 2). The combined organic solution was washed with brine, dried over anhydrous sodium sulfate, and concentrated. The crude was purified by flash column chromatography (0–20% MeOH-DCM gradient with 0.2% ammonia, 80 g RediSep(R) Rf column, Teledyne ISCO, Lincoln, NE). The title compound was obtained as a yellow solid (1.5230 g, 3.693 mmol, 74% yield). TLC Rf 0.45 (17% MeOH-DCM, UV). ^{1}H NMR (400 MHz, $CDCl_3$) δ 7.40 – 7.22 (m, 5H), 5.14 (s, 2H), 4.49 (s, 2H), 4.38 (dd, *J* = 10.8, 4.8 Hz, 1H), 4.16 (dd, *J* = 10.7, 6.6 Hz, 1H), 3.95 (s, 3H), 3.68 (t, *J* = 5.9 Hz, 2H), 3.06 (ddd, *J* = 9.4, 7.6, 1.7 Hz, 1H), 2.70 – 2.50 (m, 3H),

2.44 (s, 3H), 2.31 – 2.18 (m, 1H), 2.08 – 1.94 (m, 1H), 1.86 – 1.65 (m, 3H). ^{13}C NMR (100 MHz, $CDCl_3$) δ 169.1, 163.0, 162.0, 155.2, 136.4, 128.5, 128.0, 127.9, 107.4, 69. 9, 67.3, 63.9, 57.6, 54.0, 47.7, 41.6, 40.6, 29.0, 22.8, 21.0. Accurate MS (ESI-TOF) calculated for $C_{22}H_{29}N_4O_4$ $[M + H]^+$ 413.2188, found 413.2235.

(*S*)-4-Methoxy-2-((1-methylpyrrolidin-2-yl)methoxy)-5,6,7,8-tetrahydropyrido[3,4-*d*]pyrimidine (28). A 100-mL round bottom flask equipped with a stir bar was charged with benzyl (*S*)-4-methoxy-2-((1-methylpyrrolidin-2-yl)methoxy)-5,8-dihydropyrido[3,4-*d*]pyrimidine-7(6*H*)-carboxylate (**27**, 1.24 g, 3.00 mmol), palladium on carbon (1.12 g, 10 wt% Pd, wet basis, 1.1 mmol, 0.36 equiv). Sealed with a rubber septum, the flask was repeatedly deaerated and backfilled with argon three times. Methanol (30 mL) was added. The resulting suspension was bubbled with hydrogen gas for 10 min, and then stirred under hydrogen atmosphere at 40 °C for 12 h, at which point the conversion of the starting material was complete as judged by LC-MS. The reaction mixture was cooled to room temperature, bubbled with argon, and, filtered through a tightly packed Celite column. The filter cake was washed with methanol (20 mL x 2). The combined filtrate was concentrated to give the title compound as a yellow solid (752 mg, 2.70 mmol, 90% yield). Proton NMR was consistent with the earlier report.[3] Accurate MS (ESI-TOF) calculated for $C_{14}H_{23}N_4O_2$ $[M + H]^+$ 279.1821, found 279.1824.

(*S*)-7-(8-Chloronaphthalen-1-yl)-4-methoxy-2-((1-methylpyrrolidin-2-yl)methoxy)-5,6,7,8-tetrahydropyrido[3,4-*d*]pyrimidine (30). A 100-mL round bottom flask equipped with a stir bar was charged with (*S*)-4-methoxy-2-((1-methylpyrrolidin-2-yl)methoxy)-5,6,7,8-tetrahydropyrido[3,4-*d*]pyrimidine (**28**, 752 mg, 2.70 mmol), 1-bromo-8-chloronaphthalene (**29**,

848 mg, 3.51 mmol, 1.3 equiv), RuPhos Pd G3 precatalyst (226 mg, 0.270 mmol, 0.10 equiv), and cesium carbonate (3.08 g, 9.45 mmol, 3.5 equiv). Sealed with a rubber septum, the flask was repeatedly deaerated and backfilled with argon three times. Degassed toluene (13.5 mL) was added. The resulting solution was stirred at 90 °C for 24 h, at which point the conversion of secondary amine starting material was complete as judged by LC-MS. The black reaction mixture was concentrated. The residue was partitioned between EA (50 mL) and water (100 mL). The two phases were separated, and the aqueous was extracted by EA (50 mL x 2). The combined organic solution was washed by half-saturated brine, brine, sequentially, dried over anhydrous sodium sulfate, and concentrated. The crude product was purified by flash column chromatography (0–20% MeOH-DCM gradient with 0.2% ammonia, 80-g RediSep(R) Rf column, Teledyne ISCO, Lincoln, NE). The title compound was obtained as a yellow solid (851.0 mg, 1.939 mmol, 72% yield). Proton NMR was consistent with the earlier report.[3] TLC Rf 0.56 (17% MeOH-DCM, UV). Accurate MS (ESI-TOF) calculated for $C_{24}H_{28}ClN_4O_2$ $[M + H]^+$ 439.1901, found 439.1918.

(*S*)-7-(8-Chloronaphthalen-1-yl)-2-((1-methylpyrrolidin-2-yl)methoxy)-5,6,7,8-tetrahydropyrido[3,4-*d*]pyrimidin-4-ol (31). A 20-mL scintillation vial equipped with a stir bar was charged with (*S*)-7-(8-Chloronaphthalen-1-yl)-4-methoxy-2-((1-methylpyrrolidin-2-yl)methoxy)-5,6,7,8-tetrahydropyrido[3,4-*d*]pyrimidine (**30**, 413 mg, 0.940 mmol), sodium ethanethiolate (158 mg, 1.88 mmol, 2.0 equiv), and DMF (5 mL) were added. The resulting mixture was stirred at 60 °C for 3 h. The reaction mixture was concentrated before partitioned between DCM (40 mL) and 10% aqueous sodium bicarbonate (40 mL). The phases were separated, and the aqueous was extracted with DCM (40 mL x 2). The combined organic phase

was dried on anhydrous sodium sulfate, filtered, and concentrated. The residue was purified by flash column chromatography (0–20% MeOH-DCM gradient with 0.2% ammonia, 24-g RediSep(R) Rf column, Teledyne ISCO, Lincoln, NE). The title compound was obtained as a yellow solid (348.2 mg, 0.819 mmol, 87% yield). Proton NMR was consistent with the earlier report.[3] TLC Rf 0.32 (17% MeOH-DCM, UV). Accurate MS (ESI-TOF) calculated for $C_{23}H_{26}ClN_4O_2$ $[M + H]^+$ 425.1744, found 425.1774.

***tert*-Butyl (*S*)-4-(7-(8-chloronaphthalen-1-yl)-2-(((*S*)-1-methylpyrrolidin-2-yl)methoxy)-5,6,7,8-tetrahydropyrido[3,4-*d*]pyrimidin-4-yl)-2-(cyanomethyl)piperazine-1-carboxylate (33).** A 4-mL vial equipped with a stir bar was charged with (*S*)-7-(8-chloronaphthalen-1-yl)-2-((1-methylpyrrolidin-2-yl)methoxy)-5,6,7,8-tetrahydropyrido[3,4-*d*]pyrimidin-4-ol (**31**, 187 mg, 0.440 mmol) and BOP (253 mg, 0.572 mmol, 1.3 equiv). Sealed by a screw cap with a PEFE septum, the vial was deaerated and backfilled with argon three times. Anhydrous MeCN (1.1 mL) and DBU (100 µL, 0.66 mmol, 1.5 equiv) were added. The resulting solution was stirred at ambient temperature for 10 min before a stock solution of *tert*-butyl (*S*)-2-(cyanomethyl)piperazine-1-carboxylate (**32**, 149 mg, 0.660 mmol, 1.5 equiv) in MeCN (1.1 mL) was added via syringe. The mixture was stirred at 60 °C for 12 h, at which point the conversion of **31** was complete. The reaction mixture was concentrated. The brown crude was purified by flash column chromatography (0–15% MeOH-DCM gradient, 24-g RediSep(R) Rf column, Teledyne ISCO, Lincoln, NE). The title compound was obtained as a yellow solid (213 mg, 0.338 mmol, 77% yield). Proton NMR is consistent to the earlier report by Blake, J. F. et al.[4] Accurate MS (ESI-TOF) calculated for $C_{34}H_{43}ClN_7O_3$ $[M + H]^+$ 632.3116, found 632.3157.

2-((*S*)-4-(7-(8-Chloronaphthalen-1-yl)-2-(((*S*)-1-methylpyrrolidin-2-yl)methoxy)-5,6,7,8-tetrahydropyrido[3,4-*d*]pyrimidin-4-yl)piperazin-2-yl)acetonitrile (34). A 20-mL scintillation vial equipped with a stir bar was charged with *tert*-butyl (*S*)-4-(7-(8-chloronaphthalen-1-yl)-2-(((*S*)-1-methylpyrrolidin-2-yl)methoxy)-5,6,7,8-tetrahydropyrido[3,4-*d*]pyrimidin-4-yl)-2-(cyanomethyl)piperazine-1-carboxylate (**33**, 213 mg, 0.338 mmol). DCM (2 mL) and trifluoroacetic acid (TFA, 2 mL) were added. The resulting mixture was stirred at ambient temperature for 1 h, at which point the conversion of the starting material was complete. The reaction mixture was concentrated in vacuo. The crude was triturated in ether, centrifugated, and the supernatant was decanted. The title compound as a di-TFA salt (254 mg, 0.335 mmol, 99% yield). The compound was used in the next step without further purification. Accurate MS (ESI-TOF) calculated for $C_{29}H_{35}ClN_7O$ $[M + H]^+$ 532.2592, found 532.2592.

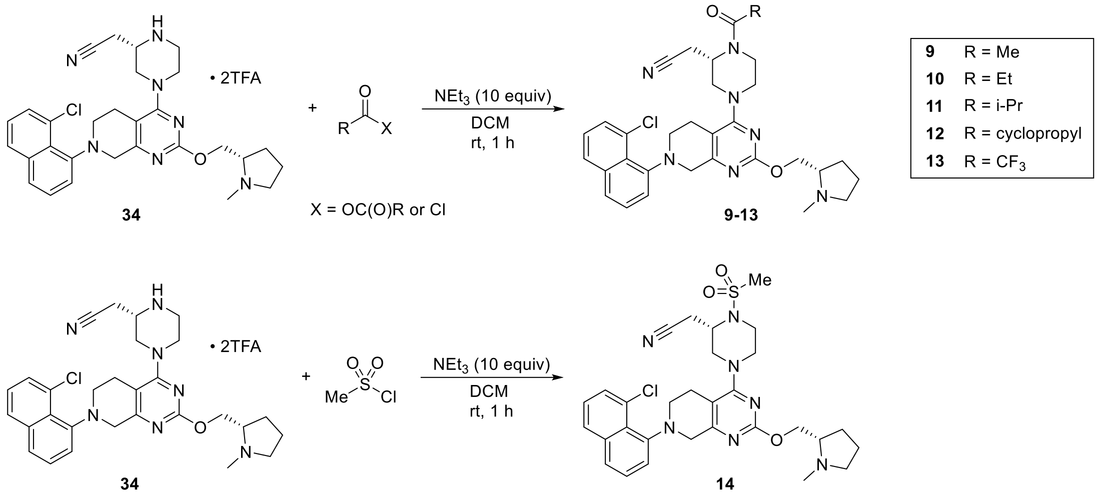

Preparation of saturated amide analogs of MRTX849 (9-14).

2-((*S*)-1-Acetyl-4-(7-(8-chloronaphthalen-1-yl)-2-(((*S*)-1-methylpyrrolidin-2-yl)methoxy)-5,6,7,8-tetrahydropyrido[3,4-*d*]pyrimidin-4-yl)piperazin-2-yl)acetonitrile (9). In a typical procedure for the synthesis of saturated amide analogs of MRTX849, A 4-mL vial equipped with a stir bar was charged with 2-((*S*)-4-(7-(8-chloronaphthalen-1-yl)-2-(((*S*)-1-methylpyrrolidin-2-yl)methoxy)-5,6,7,8-tetrahydropyrido[3,4-*d*]pyrimidin-4-yl)piperazin-2-yl)acetonitrile (**34**, 5.0 mg, 0.0094 mmol) and DCM (1 mL). Triethylamine (13 µL, 0.094 mmol, 10 equiv) and acetic anhydride (4.4 µL, 0.047 mmol, 5 equiv) were added, sequentially. The mixture was stirred at 23 °C for 1 h, at which point the conversion of the starting material was complete. The reaction mixture was concentrated. The residue was dissolved in 0.1 mL DMSO and 0.9 mL (50% MeCN-water). The resulting solution was filtered through a 0.45 µM PTFE syringe filter. The filtrate was purified by reverse-phase HPLC (Waters XBridge C18 column 5 µm particle size 30 x 250 mm, 5–95% acetonitrile–water + 0.1% formic acid, 40 min, 20 mL/min) to afford the title compound in its 1:1 formic acid salt form as a white fluffy solid (1.7 mg, 0.0030 mmol, 29% yield). ^{1}H NMR (400 MHz, DMSO-d_6) δ 7.92 (d, *J* = 8.1 Hz, 1H), 7.78 – 7.70 (m, 1H), 7.62 –

7.50 (m, 2H), 7.45 (t, *J* = 7.8 Hz, 1H), 7.34 (dd, *J* = 14.8, 6.9 Hz, 1H), 4.94 – 4.79 (m, 1H), 4.60 – 4.28 (m, 1H), 4.24 (ddt, *J* = 9.7, 4.8, 2.2 Hz, 1H), 4.16 (d, *J* = 16.1 Hz, 1H), 4.12 – 3.65 (m, 5H), 3.64 – 3.44 (m, 2H, inaccurate integrate due to HOD contamination), 3.26 – 2.62 (m, 10H), 2.55 (q, *J* = 6.8 Hz, 1H), 2.34 (d, *J* = 4.1 Hz, 3H), 2.25 – 2.12 (m, 3H), 2.07 (d, *J* = 1.6 Hz, 2H), 2.01 – 1.86 (m, 1H), 1.73 – 1.53 (m, 3H). ^{13}C NMR (151 MHz, DMSO-d6, major rotamer) δ 169.2, 168.6, 166.1, 164.2, 162.0, 148.1, 137.0, 129.5, 128.9, 128.6, 126.9, 126.0, 125.0, 118.8, 118.5, 109.0, 108.4, 68.9, 63.4, 58.7, 57.0, 50.1, 49.1 47.5, 45.4, 41.3, 35.7, 28.6, 25.5, 22.5, 21.2, 17.8. Accurate MS (ESI-TOF) calculated for $C_{31}H_{37}ClN_7O_2$ $[M + H]^+$ 574.2697, found 574.2703.

2-((*S*)-4-(7-(8-Chloronaphthalen-1-yl)-2-(((*S*)-1-methylpyrrolidin-2-yl)methoxy)-5,6,7,8-tetrahydropyrido[3,4-*d*]pyrimidin-4-yl)-1-propionylpiperazin-2-yl)acetonitrile (10). The title compound was synthesized via the general MRTX849 analog preparation procedure. 2-((*S*)-4-(7-(8-chloronaphthalen-1-yl)-2-(((*S*)-1-methylpyrrolidin-2-yl)methoxy)-5,6,7,8-tetrahydropyrido[3,4-*d*]pyrimidin-4-yl)piperazin-2-yl)acetonitrile (5.0 mg, 0.0094 mmol), triethylamine (13 µL, 0.094 mmol, 10 equiv) and propionyl chloride (4.1 µL, 0.047 mmol, 5 equiv) afforded the title compound in its 1:1 formic acid salt form (4.6 mg, 0.007 mmol, 77% yield) as a white fluffy solid. ^{1}H NMR (400 MHz, DMSO-d_6) δ 7.93 (ddt, *J* = 8.1, 1.3, 0.6 Hz, 1H), 7.75 (ddd, *J* = 8.6, 4.5, 1.1 Hz, 1H), 7.61 – 7.50 (m, 2H), 7.45 (dd, *J* = 8.1, 7.4 Hz, 1H), 7.39 – 7.29 (m, 1H), 5.00 – 4.75 (m, 1H), 4.65 – 4.32 (m, 1H), 4.24 (ddd, *J* = 9.5, 4.8, 2.8 Hz, 1H), 4.16 (d, *J* = 15.5 Hz, 1H), 4.11 – 3.88 (m, 3H), 3.78 (dd, *J* = 36.6, 14.4 Hz, 3H), 3.64 – 2.62 (m, 15H, inaccurate integrate due to HOD contamination), 2.56 (h, *J* = 5.3 Hz, 1H), 2.35 (d, *J* = 4.2 Hz, 3H), 2.19 (qd, *J* = 8.7, 3.1 Hz, 1H), 2.00 – 1.86 (m, 1H), 1.73 – 1.55 (m, 3H), 1.02 (td, *J*

= 11.3, 9.8, 6.6 Hz, 3H). ^{13}C NMR (151 MHz, DMSO-d6, major rotamer) δ 172.2, 171.7, 165.9, 164.1, 162.0, 148.1, 137.0, 129.5, 128.9, 128.6, 126.9, 126.0, 125.0, 124.8, 118.8, 118.5, 109.0, 108.5, 68.6, 63.6, 58.8, 57.0, 50.1, 49.1, 47.5, 45.6, 41.2, 35.9, 28.4, 25.9, 22.5, 18.4, 17.8, 9.3. Accurate MS (ESI-TOF) calculated for $C_{32}H_{39}ClN_7O_2$ [M + H]$^+$ 588.2854, found 588.2875.

2-((*S*)-4-(7-(8-Chloronaphthalen-1-yl)-2-(((*S*)-1-methylpyrrolidin-2-yl)methoxy)-5,6,7,8-tetrahydropyrido[3,4-*d*]pyrimidin-4-yl)-1-isobutyrylpiperazin-2-yl)acetonitrile (11). The title compound was synthesized via the general MRTX849 analog preparation procedure. 2-((*S*)-4-(7-(8-chloronaphthalen-1-yl)-2-(((*S*)-1-methylpyrrolidin-2-yl)methoxy)-5,6,7,8-tetrahydropyrido[3,4-*d*]pyrimidin-4-yl)piperazin-2-yl)acetonitrile (5.0 mg, 0.009 mmol), triethylamine (13 µL, 0.094 mmol, 10 equiv) and isobutyryl chloride (4.9 µL, 0.047 mmol, 5 equiv) afforded the title compound in its 1:1 formic acid salt form (3.3 mg, 0.005 mmol, 54% yield) as a white fluffy solid. ^{1}H NMR (400 MHz, DMSO-d_6) δ 7.93 (dt, *J* = 8.4, 1.0 Hz, 1H), 7.75 (ddd, *J* = 8.4, 4.0, 1.1 Hz, 1H), 7.62 – 7.50 (m, 2H), 7.46 (dd, *J* = 8.1, 7.4 Hz, 1H), 7.40 – 7.30 (m, 1H), 5.01 – 4.87 (m, 1H), 4.29 – 4.12 (m, 2H), 4.10 – 3.89 (m, 3H), 3.89 – 3.68 (m, 2H), 3.64 – 2.78 (m, 20H), 2.71 (t, *J* = 14.5 Hz, 1H), 2.34 (d, *J* = 3.8 Hz, 3H), 2.17 (qd, *J* = 8.7, 2.5 Hz, 1H), 1.92 (dq, *J* = 12.1, 8.2 Hz, 1H), 1.73 – 1.52 (m, 3H), 1.11 – 0.97 (m, 6H). ^{13}C NMR (151 MHz, DMSO-6, TFA salt instead of formic acid salt, major rotamer) δ 175.3, 165.8, 163.8, 161.0, 147.8, 137.1, 129.6, 128.8, 128.6, 126.9, 126.0, 125.0, 118.9, 118.4, 109.0, 66.6, 64.21, 58.5, 56.5, 50.0, 49.1, 47.9, 47.7, 45.4, 40.4, 38.4, 35.8, 29.4, 26.3, 25.6, 22.7, 21.8, 20.3, 19.2, 17.8. Accurate MS (ESI-TOF) calculated for $C_{33}H_{41}ClN_7O_2$ [M + H]$^+$ 602.3010, found 602.3030.

2-((*S*)-4-(7-(8-Chloronaphthalen-1-yl)-2-(((*S*)-1-methylpyrrolidin-2-yl)methoxy)-5,6,7,8-tetrahydropyrido[3,4-*d*]pyrimidin-4-yl)-1-(cyclopropanecarbonyl)piperazin-2-yl)acetonitrile (12). The title compound was synthesized via the general MRTX849 analog preparation procedure. 2-((*S*)-4-(7-(8-chloronaphthalen-1-yl)-2-(((*S*)-1-methylpyrrolidin-2-yl)methoxy)-5,6,7,8-tetrahydropyrido[3,4-*d*]pyrimidin-4-yl)piperazin-2-yl)acetonitrile (5.0 mg, 0.009 mmol), triethylamine (13 µL, 0.094 mmol, 10 equiv) and cyclopropanecarbonyl chloride (4.3 µL, 0.047 mmol, 5 equiv) afforded the title compound in its 1:1 formic acid salt form (2.4 mg, 0.004 mmol, 40% yield) as a white fluffy solid. ^{1}H NMR (400 MHz, DMSO-d_6) δ 7.93 (dt, *J* = 8.5, 1.2 Hz, 1H), 7.75 (ddd, *J* = 8.6, 4.5, 1.1 Hz, 1H), 7.62 – 7.50 (m, 2H), 7.46 (dd, *J* = 8.1, 7.4 Hz, 1H), 7.40 – 7.30 (m, 1H), 4.93 (s, 1H), 4.45 – 4.11 (m, 3H), 4.13 – 3.91 (m, 3H), 3.79 (dd, *J* = 43.3, 17.3 Hz, 3H), 3.58 – 2.99 (m, 16H, inaccurate integrate due to HOD contamination), 2.99 – 2.79 (m, 3H), 2.71 (s, 1H), 2.35 (d, *J* = 4.1 Hz, 3H), 2.18 (qd, *J* = 8.7, 2.8 Hz, 1H), 2.13 – 1.98 (m, 1H), 1.93 (dq, *J* = 12.1, 8.1 Hz, 1H), 1.75 – 1.54 (m, 3H), 0.90 – 0.70 (m, 5H). ^{13}C NMR (151 MHz, DMSO-d6, TFA salt instead of formic acid salt, major rotamer) δ 172.0, 165.8, 164.2, 161.1, 148.0, 137.1, 129.6, 128.9, 128.6, 126.9, 126.0, 125.0, 118.9, 118.6, 109.0, 66.6, 64.1, 58.7, 56.4, 50.0, 49.4, 47.4, 46.1, 40.4, 26.3, 25.6, 21.8, 18.9, 17.7, 10.7, 7.6. Accurate MS (ESI-TOF) calculated for $C_{33}H_{39}ClN_7O_2$ [M + H]$^+$ 600.2853, found 600.2853.

2-((*S*)-4-(7-(8-Chloronaphthalen-1-yl)-2-(((*S*)-1-methylpyrrolidin-2-yl)methoxy)-5,6,7,8-tetrahydropyrido[3,4-*d*]pyrimidin-4-yl)-1-(2,2,2-trifluoroacetyl)piperazin-2-yl)acetonitrile (13). The title compound was synthesized via the general MRTX849 analog preparation procedure. 2-((*S*)-4-(7-(8-chloronaphthalen-1-yl)-2-(((*S*)-1-methylpyrrolidin-2-yl)methoxy)-5,6,7,8-tetrahydropyrido[3,4-*d*]pyrimidin-4-yl)piperazin-2-yl)acetonitrile (5.0 mg, 0.009 mmol),

triethylamine (13 µL, 0.094 mmol, 10 equiv) and trifluoroacetic anhydride (8.6 µL, 0.047 mmol, 5 equiv) afforded the title compound in its 1:1 formic acid form (1.9 mg, 0.003 mmol, 30% yield) as a white fluffy solid. ^{1}H NMR (400 MHz, DMSO-d_6) δ 7.96 – 7.90 (m, 1H), 7.75 (ddd, *J* = 8.4, 3.4, 1.1 Hz, 1H), 7.61 – 7.50 (m, 2H), 7.46 (dd, *J* = 8.1, 7.5 Hz, 1H), 7.40 – 7.31 (m, 1H), 4.92 (s, 1H), 4.36 – 3.57 (m, 8H), 3.50 (q, *J* = 7.2 Hz, 2H), 3.20 – 2.88 (m, 6H), 2.71 (d, *J* = 14.4 Hz, 1H), 2.56 (d, *J* = 6.1 Hz, 1H), 2.36 (d, *J* = 4.1 Hz, 3H), 2.20 (td, *J* = 8.6, 3.6 Hz, 1H), 2.01 – 1.87 (m, 1H), 1.75 – 1.53 (m, 3H), 1.15 (dt, *J* = 23.3, 7.2 Hz, 1H). ^{13}C NMR (151 MHz, DMSO, TFA salt instead of formic acid salt, major rotamer) δ 165.7, 164.2, 161.0, 155.0, 147.8, 137.1, 129.6, 128.8, 128.6, 126.9, 126.0, 124.9, 119.0, 117.9, 117.0, 115.0, 109.4, 66.6, 64.2, 58.5, 56.4, 50.0, 49.0, 48.1, 47.6, 47.1, 40.5, 26.3, 25.2, 21.8, 18.6, 17.7. ^{19}F NMR (564 MHz, DMSO-d6) δ -67.0 (minor rotamer), -68.3 (major rotamer), -74.5 (TFA). Accurate MS (ESI-TOF) calculated for $C_{31}H_{34}ClF_3N_7O_2$ $[M + H]^+$ 628.2414, found 628.2401.

2-((*S*)-4-(7-(8-chloronaphthalen-1-yl)-2-(((*S*)-1-methylpyrrolidin-2-yl)methoxy)-5,6,7,8-tetrahydropyrido[3,4-*d*]pyrimidin-4-yl)-1-(methylsulfonyl)piperazin-2-yl)acetonitrile (14). The title compound was synthesized via the general MRTX849 analog preparation procedure. 2-((*S*)-4-(7-(8-chloronaphthalen-1-yl)-2-(((*S*)-1-methylpyrrolidin-2-yl)methoxy)-5,6,7,8-tetrahydropyrido[3,4-*d*]pyrimidin-4-yl)piperazin-2-yl)acetonitrile (5.0 mg, 0.009 mmol), triethylamine (13 µL, 0.094 mmol, 10 equiv) and methanesulfonyl chloride (3.6 µL, 0.047 mmol, 5 equiv) afforded the title compound in its 1:1 formic acid salt form (1.2 mg, 0.002 mmol, 19% yield) as a white fluffy solid. ^{1}H NMR (400 MHz, DMSO-d_6) δ 7.93 (dt, *J* = 8.5, 1.2 Hz, 1H), 7.75 (ddd, *J* = 8.4, 4.8, 1.1 Hz, 1H), 7.62 – 7.50 (m, 2H), 7.46 (dd, *J* = 8.1, 7.4 Hz, 1H), 7.35 (ddd, *J* = 20.4, 7.6, 1.2 Hz, 1H), 4.36 (s, 1H), 4.32 – 4.25 (m, 1H), 4.23 – 4.08 (m, 2H), 4.02 –

3.90 (m, 2H), 3.86 – 3.64 (m, 3H), 3.51 (q, *J* = 10.6 Hz, 3H), 3.26 – 2.90 (m, 12H), 2.68 (d, *J* = 14.4 Hz, 2H), 2.42 (d, *J* = 5.8 Hz, 3H), 2.36 – 2.25 (m, 1H), 1.97 (dq, *J* = 12.1, 8.4, 8.0 Hz, 1H), 1.78 – 1.56 (m, 3H). ^{13}C NMR (151 MHz, DMSO-d6, TFA salt instead of formic acid salt, major rotamer) δ 165.96, 164.07, 161.03, 147.96, 137.03, 129.59, 128.83, 128.63, 126.91, 126.01, 124.91, 118.95, 109.34, 66.61, 64.19, 58.61, 56.42, 49.90, 49.69, 48.44, 47.18, 40.41, 40.06, 26.25, 25.53, 25.16, 21.85, 19.17. Accurate MS (ESI-TOF) calculated for $C_{30}H_{37}ClN_7O_3S$ $[M + H]^+$ 610.2367, found 610.2371.

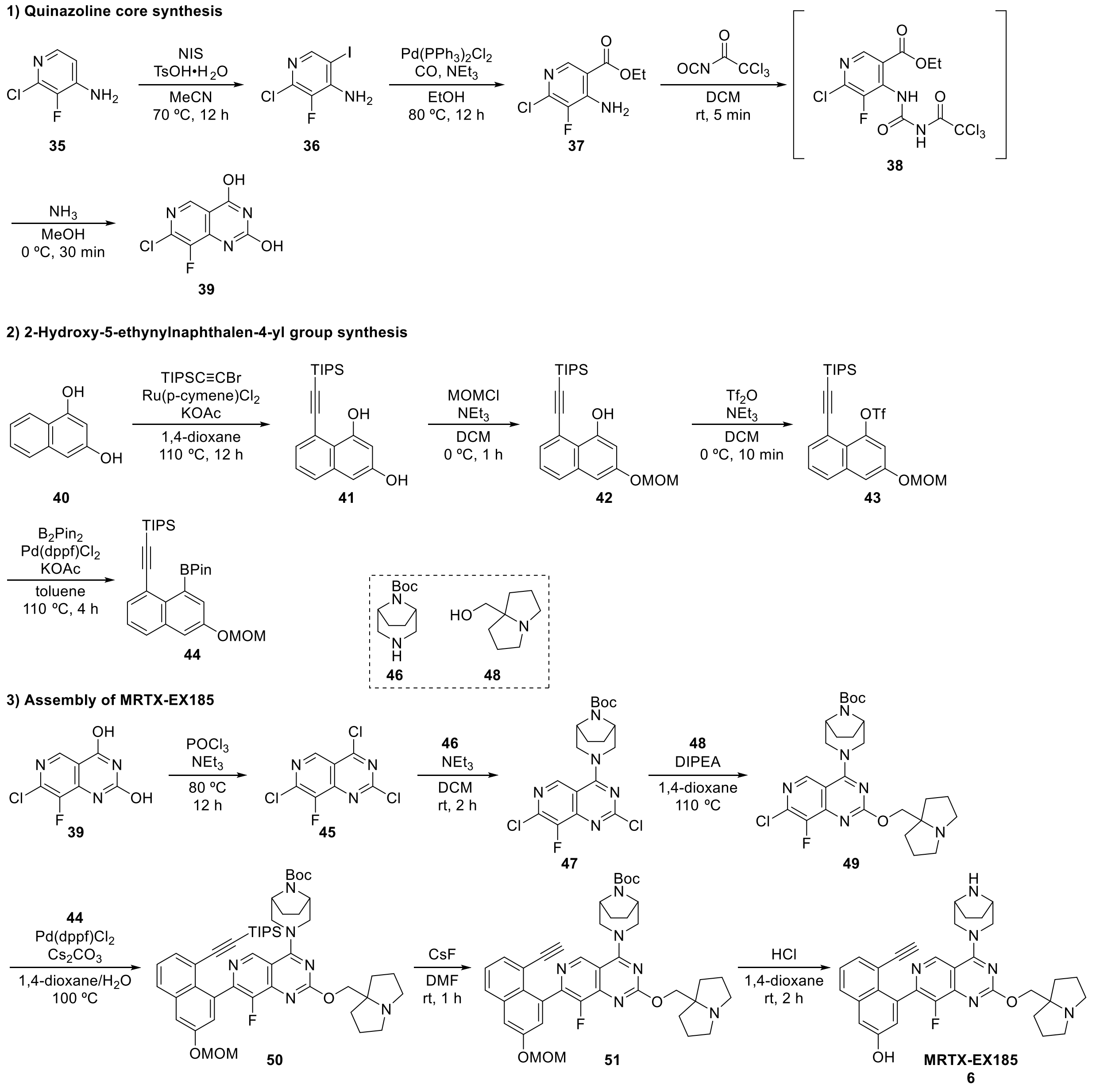

Preparation of MRTX-EX185 (6).

Synthesis of MRTX-EX185 was adapted from the patent application US2020/048194[5] with modifications.

2-Chloro-3-fluoro-5-iodopyridin-4-amine (36). A 20-mL scintillation vial equipped with a stir bar was charged with commercially available 2-chloro-3-fluoro-pyridin-4-amine (**35**, 1.00 g,

6.82 mmol), *N*-Iodosuccinimide (1.84 g, 8.19 mmol), *p*-toluenesulfonic acid monohydrate (64.9 mg, 0.341 mmol) and MeCN (5 mL). The mixture was stirred vigorously at 70 °C for 12 h, at which point the conversion of the starting material was complete as judged by LC-MS. The reaction mixture was concentrated. The residue was purified by flash column chromatography to give the title compound (1.82 g, 6.67 mmol, 98% yield) as an orange crystalline solid. ^{1}H NMR (400 MHz, $CDCl_3$) δ 8.17 (d, *J* = 0.7 Hz, 1H), 4.85 (s, 2H). Accurate MS (ESI-TOF) calculated for $C_5H_4ClFIN_2$ $[M + H]^+$ 272.9092, found 272.9138.

Ethyl 4-amino-6-chloro-5-fluoronicotinate (37). A 150-mL round bottom flask equipped with a stir bar was charged with **36** (1.80 g, 6.60 mmol), bis(triphenylphosphine)palladium(II) dichloride (463 mg, 0.660 mmol, 0.10 equiv), and ethanol (33 mL). The flask sealed with a rubber septum was deaerated and backfilled with Argon three times. Carbon monoxide (CO) gas in a balloon was introduced into the reaction flask via a long needle by submerging the needle tip in the stirred suspension. The CO flow-through was maintained for 10 min before the hydrogen gas needle retracted into the headspace above the suspension, and the exit needle removed from the septum. The mixture was stirred at 80 °C for 4 h, at which point the conversion of **36** was complete as judged by LC-MS. The reaction mixture was concentrated. The residue was partitioned between EA (100 mL) and water (100 mL). The phases were separated, and the aqueous phase was further extracted with EA (50 mL x 3). The combined organic phase was washed with brine, dried on anhydrous sodium sulfate, and concentrated. The crude was purified by flash column chromatography (0–50% EA-hexanes gradient, 40-g RediSep(R) Rf column, Teledyne ISCO, Lincoln, NE) to give the title compound (1.07 g, 4.91 mmol, 74% yield) as a yellow crystalline solid. ^{1}H NMR (400 MHz, $CDCl_3$) δ 8.53 (s, 1H), 4.38 (q, *J* = 7.1 Hz, 2H),

1.40 (t, J = 7.1 Hz, 3H). ^{19}F NMR (376 MHz, $CDCl_3$) δ -145.50. Accurate MS (ESI-TOF) calculated for $C_8H_9ClFIN_2O_2$ $[M + H]^+$ 219.0336, found 219.0383.

Ethyl 6-chloro-5-fluoro-4-(3-(2,2,2-trichloroacetyl)ureido)nicotinate (38). A 20-mL scintillation vial equipped with a stir bar was charged with **37** (655.8 mg, 3.000 mmol) and THF (5 mL). The vial sealed with a screw cap with silicone septum was deaerated and backfilled with Argon three times. 2,2,2-Trichloroacetyl isocyanate (0.43 mL, 3.6 mmol, 1.2 equiv) was added. The mixture was stirred at ambient temperature for 10 min, at which point the conversion of **37** was complete as judged by LC-MS. The reaction mixture was concentrated. The residue was triturated in diethyl ether. The title compound (1.1210 g, 2.7543 mmol, 92% yield) was obtained by filtration as a grey powder. Accurate MS (ESI-TOF) calculated for $C_{11}H_9Cl_4FN_3O_4$ $[M + H]^+$ 405.9331, found 405.9284.

7-Chloro-8-fluoropyrido[4,3-d]pyrimidine-2,4-diol (39). A 20-mL scintillation vial equipped with a stir bar was charged with **38** (1098.9 mg, 2.7000 mmol). Sealed with a rubber septum, the vial was deaerated and backfilled with Argon three times. Methanol (13.5 mL) and 7N ammonia (1.54 mL, 10.8 mmol, 4.0 equiv) were added, sequentially. The resulting suspension was stirred at ambient temperature for 1 h, at which point the conversion of **38** was complete as judged by LC-MS. The reaction mixture was concentrated *in vacuo*. The solid residue was triturated in MTBE (10 mL). The title compound (643.8 mg, 2.987 mmol, 110% yield) was obtained by filtration as an off-white solid. ^{1}H NMR (400 MHz, DMSO-d_6) δ 8.33 (s, 1H), 3.17 (s, 2H). Accurate MS (ESI-TOF) calculated for $C_7H_4ClFN_3O_2$ $[M + H]^+$ 215.9976, found 215.9794.

8-((Triisopropylsilyl)ethynyl)naphthalene-1,3-diol (41). A Schlenk tube equipped with a stir bar was charged with naphthalene-1,3-diol (**40**, 1000.0 mg, 6.2434 mmol), 2-bromoethynyl(triisopropyl)silane (1957.4 mg, 7.4916 mmol, 1.2 equiv), dichloro(*p*-cymene)ruthenium(II) dimer (382.3 mg, 0.6243 mmol, 0.10 equiv), and potassium acetate (1.23 g, 12.5 mmol, 2.5 equiv). The tube was deaerated and backfilled with Argon three times. Degassed 1,4-dioxane (12.5 mL) was added. The reaction mixture was stirred at 110 °C for 12 h, at which point the conversion of the starting material was complete as judged by LC-MS. The reaction mixture was partitioned between EA (100 mL) and water (100 mL). The phases were separated. The aqueous phase was further extracted with EA (50 mL x 2). The combined organic phase was washed with brine, dried on anhydrous sodium sulfate, and concentrated. The crude was purified by flash column chromatography (0–25% EA-hexanes, 24-g RediSep(R) Rf column, Teledyne ISCO, Lincoln, NE) to give the title compound as a grey solid (939.0 mg, 2.7574 mmol, 44% yield). ^{1}H NMR (400 MHz, $CDCl_3$) δ 9.29 (s, 1H), 7.62 (dd, *J* = 8.3, 1.2 Hz, 1H), 7.46 (dd, *J* = 7.2, 1.2 Hz, 1H), 7.29 (dd, *J* = 8.3, 7.1 Hz, 1H), 6.74 (d, *J* = 2.5 Hz, 1H), 6.62 (d, *J* = 2.5 Hz, 1H), 1.22 – 1.14 (m, 21H). Accurate MS calculated for $C_{21}H_{29}O_2Si$ $[M + H]^+$ 341.1937, found 341.1924.

3-(Methoxymethoxy)-8-((triisopropylsilyl)ethynyl)naphthalen-1-ol (42). A 25-mL round bottom flask equipped with a stir bar was charged with **41** (900.1 mg, 2.643 mmol). Sealed with a rubber septum, the flask was deaerated and backfilled with Argon three times. DCM (8.8 mL) and *N*,*N*-diisopropylethylamine (1.38 mL, 7.93 mmol, 3.0 equiv) were added. The resulting solution was cooled to 0 °C. Chloromethyl methyl ether (3.5 M in toluene, 1.13 mL, 4.0 mmol, 1.5 equiv) was added. The reaction mixture was stirred at 0 °C for 1 h, at which point the

conversion of the starting material plateaued at about 75% as judged by TLC. The reaction mixture was concentrated. The crude was purified by flash column chromatography (0–20% EA-hexanes gradient over 15 min then hold for 10 min, 40-g RediSep(R) Rf column, Teledyne ISCO, Lincoln, NE, 40 mL/min flow rate) to give the title compound as a yellow oil (673.1 mg, 1.750 mmol, 66% yield). ^{1}H NMR (400 MHz, $CDCl_3$) δ 9.25 (s, 1H), 7.72 – 7.65 (m, 1H), 7.49 (dd, *J* = 7.1, 1.2 Hz, 1H), 7.30 (dd, *J* = 8.3, 7.1 Hz, 1H), 6.97 (d, *J* = 2.4 Hz, 1H), 6.76 (d, *J* = 2.4 Hz, 1H), 5.26 (s, 2H), 3.50 (s, 3H), 1.24 – 1.14 (m, 21H).

3-(Methoxymethoxy)-8-((triisopropylsilyl)ethynyl)naphthalen-1-yl trifluoromethanesulfonate (43). A 20-mL scintillation vial equipped with a stir bar was charged with **42** (660.2 mg, 1.717 mmol). The vial sealed with a rubber septum was deaerated and backfilled with Argon three times. DCM (7.8 mL) and *N*,*N*-diisopropylethylamine (0.90 mL, 5.1 mmol, 3.0 equiv) were added. The resulting mixture was cooled to –40 °C with an external acetonitrile-dry ice bath. Trifluoromethanesulfonic anhydride (0.43 mL, 2.6 mmol, 1.5 equiv) was added slowly via a syringe with the syringe needle immersed under the solution surface. The reaction solution was stirred at –40 °C for 30 min, at which point the conversion of the starting material was complete as judged by TLC. The reaction mixture was quenched with water (5 mL). The phases were separated, and the aqueous phase was extracted with DCM (10 mL). The combined organic solution was dried over anhydrous sodium sulfate, filtered, and concentrated. The crude was purified by flash column chromatography (0–15% EA-hexanes, 24-g RediSep(R) Rf column, Teledyne ISCO, Lincoln, NE) to give the title compound (676.8 mg, 1.310 mmol, 76% yield) as a yellow syrup. ^{1}H NMR (400 MHz, $CDCl_3$) δ 7.76 – 7.71 (m, 2H), 7.46 – 7.40 (m, 2H), 7.31 (d, *J* = 2.4 Hz, 1H), 5.29 (s, 2H), 3.52 (s, 3H), 1.36 – 1.09 (m, 21H).

Triisopropyl((6-(methoxymethoxy)-8-(4,4,5,5-tetramethyl-1,3,2-dioxaborolan-2-yl)naphthalen-1-yl)ethynyl)silane (44). A 4-mL screw vial equipped with a stir bar was charged with **43** (48.0 mg, 0.0929 mmol), bis(pinacolato)diboron (47.2 mg, 0.186 mmol, 2.0 equiv), [1,1'-bis(diphenylphosphino)ferrocene]palladium(II) dichloride (6.8 mg, 0.0093 mmol, 0.10 equiv), and potassium acetate (31.9 mg, 0.325 mmol, 3.5 equiv). Sealed with a screw cap with PTFE septum, the vial was deaerated and backfilled with Argon three times. Toluene (0.37 mL) was added. The mixture was stirred to 110 °C for 4 h, at which point the conversion of the starting material was complete. The reaction mixture was cooled to room temperature, and partitioned between EA (20 mL) and water (20 mL). The phases were separated, and the aqueous solution was further extracted with EA (20 mL x 3). The combined organic solution was washed with brine, dried over anhydrous sodium sulfate, and concentrated. The crude was purified by flash column chromatography (0–10% EA-Hexanes gradient, 4-g RediSep(R) Rf column, Teledyne ISCO, Lincoln, NE) to give the title compound (24.9 mg, 0.0503 mmol, 54% yield) as a yellow solid. ^{1}H NMR (400 MHz, $CDCl_3$) δ 7.71 – 7.66 (m, 2H), 7.47 (d, *J* = 2.6 Hz, 1H), 7.37 (d, *J* = 2.6 Hz, 1H), 7.34 (dd, *J* = 8.2, 7.2 Hz, 1H), 5.28 (s, 2H), 3.50 (s, 3H), 1.43 (s, 12H), 1.15 (d, *J* = 2.1 Hz, 21H).

***tert*-Butyl (1R,5S)-3-(2,7-dichloro-8-fluoropyrido[4,3-d]pyrimidin-4-yl)-3,8-diazabicyclo[3.2.1]octane-8-carboxylate (47).** A 25-mL round bottom flask equipped with a stir bar was charged with **39** (215.6 mg, 1.000 mmol). The flask sealed with a rubber septum was deaerated and backfilled with Argon three times. Phosphoryl chloride (4.7 mL, 50 mmol, 50 equiv) was added. The resulting mixture was cooled to 0 ºC. *N*,*N*-diisopropylethylamine (0.9

mL, 5 mmol) was added drop wise. The mixture was stirred at 110 °C for 6 h. Cooled to room temperature, the reaction mixture was concentrated. The residual $POCl_3$ was azeotropically removed with chloroform to give crude 2,4,7-trichloro-8-fluoro-pyrido[4,3-d]pyrimidine (**45**) as a black syrup. This crude sample was used in the following step without purification or characterization.

Crude 2,4,7-trichloro-8-fluoro-pyrido[4,3-d]pyrimidine (**45**) was dissolved in DCM (10 mL) in a 100-mL round bottom flask. *tert*-Butyl 3,8-diazabicyclo[3.2.1]octane-8-carboxylate (**46**, 212.3 mg, 1.000 mmol) and *N*,*N*-diisopropylethylamine (0.50 mL, 3.0 mmol, 3 equiv) were added. The resulting solution was stirred at ambient temperature for 5 min, at which point conversion of the starting material was complete as judged by LC-MS. The reaction mixture was concentrated. The crude was purified by flash column chromatography (0–100% EA-hexanes, 12-g RediSep(R) Rf column, Teledyne ISCO, Lincoln, NE) to give the title compound (278.1 mg, 0.6493 mmol, 65% yield for two steps) as a white crystalline solid. ^{1}H NMR (400 MHz, $CDCl_3$) δ 8.84 (d, J = 0.6 Hz, 1H), 4.70 – 4.22 (m, 4H), 3.72 (s, 2H), 1.98 (dd, J = 8.7, 4.3 Hz, 2H), 1.67 (d, J = 7.7 Hz, 2H), 1.51 (s, 9H). Accurate MS (ESI-TOF) calculated for $C_{18}H_{21}Cl_2FN_5O_2$ $[M + H]^+$ 428.1056, found 428.1121.

***tert*-Butyl (1R,5S)-3-(7-chloro-8-fluoro-2-((tetrahydro-1H-pyrrolizin-7a(5H)-yl)methoxy)pyrido[4,3-d]pyrimidin-4-yl)-3,8-diazabicyclo[3.2.1]octane-8-carboxylate (49).**

A 20-mL vial equipped with a stir bar was charged with **47** (364.0 mg, 0.8499 mmol). Sealed with a screw cap with a PTFE septum, the vial was deaerated and backfilled with Argon three times. 1,2,3,5,6,7-Hexahydropyrrolizin-8-ylmethanol (**48**, 240.1 mg, 1.700 mmol, 2 equiv) in 1,4-dioxane (4.3 mL) and *N*,*N*-diisopropylethylamine (0.44 mL, 2.6 mmol, 3 equiv) were added

sequentially. The mixture was stirred at 80 °C for 6 h, at which point the conversion of the starting material was complete as judged by LC-MS. The reaction mixture was concentrated. The residue was partitioned between water (10 mL) and EA (10 mL). The phases were separated, and the aqueous phase was further extracted with EA (10 mL x 2). The combined organic solution was washed with brine, dried over anhydrous sodium sulfate, and concentrated. The crude was purified by flash column chromatography (0–20% MeOH-DCM containing 2% ammonia, 12-g RediSep(R) Rf column, Teledyne ISCO, Lincoln, NE) to give the title compound (325.1 mg, 0.6099 mmol, 72% yield) as a white foam. ^{1}H NMR (400 MHz, $CDCl_3$) δ 8.74 (d, *J* = 0.5 Hz, 1H), 5.30 (s, 1H), 4.80 (s, 2H), 4.40 (s, 3H), 3.92 (dq, *J* = 12.9, 6.6 Hz, 2H), 3.81 (d, *J* = 7.7 Hz, 1H), 3.70 (dq, *J* = 12.8, 6.7 Hz, 2H), 3.04 – 2.88 (m, 3H), 2.47 – 1.91 (m, 15H), 1.86 – 1.66 (m, 3H), 1.63 (s, 9H). Accurate MS (ESI-TOF) calculated for $C_{26}H_{35}ClFN_6O_3$ $[M + H]^+$ 533.2443, found 533.2467.

***tert*-Butyl (1R,5S)-3-(8-fluoro-7-(3-(methoxymethoxy)-8-((triisopropylsilyl)ethynyl)naphthalen-1-yl)-2-((tetrahydro-1H-pyrrolizin-7a(5H)-yl)methoxy)pyrido[4,3-d]pyrimidin-4-yl)-3,8-diazabicyclo[3.2.1]octane-8-carboxylate (50).** A 20-mL scintillation vial equipped with a stir bar was charged with **49** (53.3 mg, 0.100 mmol), **44** (64.3 mg, 0.130 mmol, 1.3 equiv), [1,1’-bis(diphenylphosphino)ferrocene]palladium(II) dichloride (14.6 mg, 0.0200 mmol, 0.2 equiv), and cesium carbonate (97.8 mg, 0.300 mmol, 3 equiv). Sealed with a screw cap with a silicone septum, the vial was deaerated and backfilled with Argon three times. 1,4-Dioxane (3 mL) and degassed water (1 mL) were added. The resulting mixture was stirred at 100 °C for 2 h, at which point the conversion of the starting material was almost complete (~98%) as judged by LC-MS. The reaction crude was purified by

reverse phase prep-HPLC (5–95% MeCN-H_2O gradient with 0.1% formic acid, 40 min method) to give the title compound in its 1:1 formic acid salt form (15.5 mg, 0.0179 mmol, 18% yield) as a white fluffy solid. ^{1}H NMR (400 MHz, DMSO) δ 9.15 (s, 1H), 8.01 (dd, *J* = 8.4, 1.4 Hz, 1H), 7.69 (d, *J* = 2.6 Hz, 1H), 7.61 (dd, *J* = 7.2, 1.4 Hz, 1H), 7.53 (dd, *J* = 8.2, 7.2 Hz, 1H), 7.28 (d, *J* = 2.6 Hz, 1H), 5.37 (s, 2H), 4.74 (d, *J* = 12.8 Hz, 1H), 4.38 – 4.15 (m, 3H), 4.00 (s, 2H), 3.74 (d, *J* = 12.4 Hz, 1H), 3.43 (s, 3H), 2.93 (dt, *J* = 10.1, 5.4 Hz, 2H), 2.58 – 2.53 (m, 1H), 1.95 – 1.52 (m, 12H), 1.46 (s, 8H), 0.81 (dd, *J* = 7.5, 6.3 Hz, 16H), 0.47 (p, *J* = 7.5 Hz, 3H). Accurate MS (ESI-TOF) calculated for $C_{49}H_{66}FN_6O_5Si$ $[M + H]^+$ 865.4848, found 865.4904.

***tert*-Butyl (1R,5S)-3-(7-(8-ethynyl-3-(methoxymethoxy)naphthalen-1-yl)-8-fluoro-2-((tetrahydro-1H-pyrrolizin-7a(5H)-yl)methoxy)pyrido[4,3-d]pyrimidin-4-yl)-3,8-diazabicyclo[3.2.1]octane-8-carboxylate (51).** A 4-mL vial equipped with a stir bar was charged with **50** (15.5 mg, 0.0179 mmol) and cesium fluoride (27.2 mg, 0.179 mmol, 1 equiv). DMF (0.5 mL) was added. The reaction mixture was stirred at ambient temperature for 30 min, at which point the conversion of the starting material was complete as judged by LC-MS. The reaction mixture was concentrated. The residue was dissolved in 70% MeCN-H_2O (4 mL), and was purified by reverse phase prep-HPLC (5–95% MeCN-H_2O gradient with 0.1% formic acid, 40 min method) to give the title compound in its 1:1 formic acid salt form (6.6 mg, 0.0087 mmol, 49% yield) as a white solid. ^{1}H NMR (400 MHz, $CDCl_3$) δ 9.00 (s, 1H), 7.83 (dd, *J* = 8.3, 1.3 Hz, 1H), 7.60 (dd, *J* = 7.2, 1.3 Hz, 1H), 7.53 (d, *J* = 2.6 Hz, 1H), 7.39 (dd, *J* = 8.2, 7.2 Hz, 1H), 7.33 (d, *J* = 2.6 Hz, 1H), 5.37 – 5.28 (m, 2H), 4.86 – 4.33 (m, 7H), 3.97 – 3.58 (m, 4H), 3.52 (s, 3H), 3.03 – 2.82 (m, 2H), 2.67 (s, 1H), 2.46 – 2.32 (m, 2H), 2.29 – 2.16 (m, 2H), 2.09 (dt, *J* =

15.0, 7.5 Hz, 2H), 2.03 – 1.89 (m, 5H), 1.76 – 1.55 (m, 11H), 1.52 (s, 9H). Accurate MS calculated for $C_{40}H_{46}FN_6O_5$ $[M + H]^+$ 709.3514, found 709.3590.

4-(4-((1R,5S)-3,8-Diazabicyclo[3.2.1]octan-3-yl)-8-fluoro-2-((tetrahydro-1H-pyrrolizin-7a(5H)-yl)methoxy)pyrido[4,3-d]pyrimidin-7-yl)-5-ethynylnaphthalen-2-ol (MRTX-EX185, 6). A 4-mL vial equipped with a stir bar was charged with **51** (2.2 mg, 0.0029 mmol). The vial sealed with a screw cap and a PTFE septum was deaerated and backfilled with Argon three times. 1,4-Dioxane (0.5 mL) was added. The resulting solution was cooled to 0 °C. Into the reaction vial, concentrated HCl solution (12 N, 0.5 mL) was added. The mixture was stirred at 0 °C for 1 h, at which point the conversion of the starting material was complete as judged by LC-MS. The mixture was concentrated. The crude was purified by reverse phase prep-HPLC (5–95% MeCN-H_2O gradient with 0.1% formic acid, 40 min method) to give the title compound in its 1:2 formic acid salt form (1.6 mg, 0.0024 mmol, 84% yield) as a white solid. ^{1}H NMR (400 MHz, MeOD) δ 9.10 (d, *J* = 4.9 Hz, 1H), 8.29 (s, 1H), 7.85 (dd, *J* = 8.4, 1.3 Hz, 1H), 7.53 (dd, *J* = 7.2, 1.3 Hz, 1H), 7.42 (dd, *J* = 8.3, 7.1 Hz, 1H), 7.36 (dd, *J* = 2.6, 1.4 Hz, 1H), 7.18 (d, *J* = 2.6 Hz, 1H), 4.81 (s, 2H), 4.69 (t, *J* = 4.3 Hz, 2H), 4.63 – 4.39 (m, 2H), 4.12 (s, 2H), 3.93 (t, *J* = 14.5 Hz, 2H), 3.71 (dt, *J* = 12.3, 6.9 Hz, 2H), 3.05 (s, 1H), 2.41 – 2.06 (m, 12H). Accurate MS (ESI-TOF) calculated for $C_{33}H_{34}FN_6O_2$ $[M + H]^+$ 565.2727, found 565.2740.

REFERENCES

1 Kessler, D. *et al.* Drugging an undruggable pocket on KRAS. *Proc Natl Acad Sci U S A* **116**, 15823, doi:10.1073/pnas.1904529116 (2019).

2 Bond, M. J., Chu, L., Nalawansha, D. A., Li, K. & Crews, C. M. Targeted Degradation of Oncogenic KRASG12C by VHL-Recruiting PROTACs. *ACS Central Science* **6**, 1367-1375, doi:10.1021/acscentsci.0c00411 (2020).

3 Fell, J. B. *et al.* Identification of the Clinical Development Candidate MRTX849, a Covalent KRASG12C Inhibitor for the Treatment of Cancer. *Journal of Medicinal Chemistry* **63**, 6679-6693, doi:10.1021/acs.jmedchem.9b02052 (2020).

4 Blake, J. F. *et al.* 5,6,7,8-Tetrahydropyrido[3,4-d]pyrimidine derivatives as KRas G12C inhibitors and their preparation. US20190144444 (2019).

5 Wang, X. *et al.* 4-(3,8-Diazabicyclo[3.2.1]octan-3-yl)pyrido[4,3-d]pyrimidines as KRas G12D inhibitors and their preparation. WO2021041671A1 (2021).
