# Supplementary material for: KRAS is vulnerable to reversible switch-II pocket engagement in cells
Source: Nat Chem Biol. 2022 Mar 21;18(6):596–604. doi: 10.1038/s41589-022-00985-w (PMC9135634; doi:10.1038/s41589-022-00985-w)
Supplement: Supplementary file 2 — Reporting Summary [file 41589_2022_985_MOESM2_ESM.pdf]

nature research

Corresponding author(s): Matthew B. Robers
Kevan M. Shokat

Last updated by author(s): Jan 12, 2022

Reporting Summary

Nature Research wishes to improve the reproducibility of the work that we publish. This form provides structure for consistency and transparency in reporting. For further information on Nature Research policies, see our Editorial Policies and the Editorial Policy Checklist.

Statistics

For all statistical analyses, confirm that the following items are present in the figure legend, table legend, main text, or Methods section.

n/a	Confirmed	
☐	☒	The exact sample size (n) for each experimental group/condition, given as a discrete number and unit of measurement
☐	☒	A statement on whether measurements were taken from distinct samples or whether the same sample was measured repeatedly
☒	☐	The statistical test(s) used AND whether they are one- or two-sided<br>*Only common tests should be described solely by name; describe more complex techniques in the Methods section.*
☒	☐	A description of all covariates tested
☒	☐	A description of any assumptions or corrections, such as tests of normality and adjustment for multiple comparisons
☐	☒	A full description of the statistical parameters including central tendency (e.g. means) or other basic estimates (e.g. regression coefficient) AND variation (e.g. standard deviation) or associated estimates of uncertainty (e.g. confidence intervals)
☒	☐	For null hypothesis testing, the test statistic (e.g. F, t, r) with confidence intervals, effect sizes, degrees of freedom and P value noted<br>*Give P values as exact values whenever suitable.*
☒	☐	For Bayesian analysis, information on the choice of priors and Markov chain Monte Carlo settings
☒	☐	For hierarchical and complex designs, identification of the appropriate level for tests and full reporting of outcomes
☒	☐	Estimates of effect sizes (e.g. Cohen's d, Pearson's r), indicating how they were calculated

Our web collection on statistics for biologists contains articles on many of the points above.

Software and code

Policy information about availability of computer code

Data collection	No software was used for data collection
Data analysis	Graphpad Prism v8 and v9 were used for the creation of plots, fitting of curves, and calculations of S.E.M. CCPNMR Analysis v3 was used to analyze 2D NMR spectra and create contour plots. Image J was used to process bioluminescence images.

For manuscripts utilizing custom algorithms or software that are central to the research but not yet described in published literature, software must be made available to editors and reviewers. We strongly encourage code deposition in a community repository (e.g. GitHub). See the Nature Research guidelines for submitting code & software for further information.

Data

Policy information about availability of data

All manuscripts must include a data availability statement. This statement should provide the following information, where applicable:

- Accession codes, unique identifiers, or web links for publicly available datasets
- A list of figures that have associated raw data
- A description of any restrictions on data availability

The authors declare that the data supporting the findings of this study are available within the article, the accompanying Source Data, the Supplementary Information, and the Supplementary Data. Additional information, resources, and reagents will be made available upon reasonable request; requests should be directed to and will be fulfilled by the Lead Contact Matthew B. Robers. Matt.Robers@promega.com

Field-specific reporting

Please select the one below that is the best fit for your research. If you are not sure, read the appropriate sections before making your selection.

☒ Life sciences ☐ Behavioural & social sciences ☐ Ecological, evolutionary & environmental sciences

For a reference copy of the document with all sections, see nature.com/documents/nr-reporting-summary-flat.pdf

Life sciences study design

All studies must disclose on these points even when the disclosure is negative.

Sample size	No sample size calculation was performed. Sample sizes were chosen on the basis of prior studies in NMR and BRET/Lumninescence experiments that showed significant effects with similar sample sizes.
Data exclusions	No data were excluded from these analyses
Replication	Replicated experiments were succesful and support the conclusions drawn in this report. In the case of inhibitor potency information, the data generated in this study were reproducible by 2-3 independent scientists. All data in main figures were reproduced by 2-3 independent scientists. Bioluminescence imaging was replicated in 2 independent experiments by the same scientist. The data in supplementary figures 3A, 5F, 9(a, b, and e), 10 (a-h), 11a, and 12 (a) were reproduced by 2 independent scientists in 2-3 independent experiments. Experiments in supplementary figures 3, 5(b,c,d,e, and g), 6(B-I), 7b, 8(b-e), 9 (c and d), 10 (i and j), 11(b and c), and 12 (b-d) were only performed once by single scientists. All attempts at replication were successful.
Randomization	No formal randomization method was used in this study to avoid mislabeling during inhibitor testing.
Blinding	Blinding was not relevant to this study because no bias could be made by the subject or the tester in the experiments performed.

Reporting for specific materials, systems and methods

We require information from authors about some types of materials, experimental systems and methods used in many studies. Here, indicate whether each material, system or method listed is relevant to your study. If you are not sure if a list item applies to your research, read the appropriate section before selecting a response.

Materials & experimental systems

n/a	Involved in the study	
☒	☐	Antibodies
☐	☒	Eukaryotic cell lines
☒	☐	Palaeontology and archaeology
☒	☐	Animals and other organisms
☒	☐	Human research participants
☒	☐	Clinical data
☒	☐	Dual use research of concern

Methods

n/a	Involved in the study	
☒	☐	ChIP-seq
☒	☐	Flow cytometry
☒	☐	MRI-based neuroimaging

Eukaryotic cell lines

Policy information about cell lines

Cell line source(s)	HEK-293 cells (ATCC, CAT# CRL-1573), HeLa cells (ATCC, CAT# CCL-2), A-375 cells (ATCC, CAT# CRL-1619), HCT-116 cells (ATCC, CAT# CCL-247), NCI-H358 cells (ATCC, CAT# CRL-5807), NCI-H647 cells (ATCC, CAT# CRL-5834), Mia PaCa-2 Cells (ATCC, CAT# CRL-1420), and SW-1990 cells (ATCC, CAT# CRL-2172) were cultured in DMEM (Gibco) + 10% FBS (Seradigm), with incubation in a humidified, 37°C/5% CO2 incubator. H1975 cells (ATCC, CAT# CRL-5908) were cultured in RPMI 1640 (GIBCO) + 10% FBS, with incubation in a humidified, 37°C/5% CO2 incubator. Cells were passed for at least two generations after cryorecovery before they were used for assays.
Authentication	We did not perform cell line authentication.
Mycoplasma contamination	All cell lines were tested mycoplasma negative using MycoAlert™ Mycoplasma Detection Kit (Lonza).
Commonly misidentified lines (See ICLAC register)	HEK293 and HeLa cells were used in this study.
